# Enterovirus 71 Protease 2A^pro^ Targets MAVS to Inhibit Anti-Viral Type I Interferon Responses

**DOI:** 10.1371/journal.ppat.1003231

**Published:** 2013-03-21

**Authors:** Bei Wang, Xueyan Xi, Xiaobo Lei, Xiaoyan Zhang, Sheng Cui, Jianwei Wang, Qi Jin, Zhendong Zhao

**Affiliations:** 1 MOH Key Laboratory of Systems Biology of Pathogens, Institute of Pathogen Biology, Chinese Academy of Medical Sciences & Peking Union Medical College, Beijing, People's Republic of China; 2 Department of Medical Laboratory Science, Fenyang College Shanxi Medical University, Fenyang, Shanxi, People's Republic of China; University of Pittsburgh, United States of America

## Abstract

Enterovirus 71 (EV71) is the major causative pathogen of hand, foot, and mouth disease (HFMD). Its pathogenicity is not fully understood, but innate immune evasion is likely a key factor. Strategies to circumvent the initiation and effector phases of anti-viral innate immunity are well known; less well known is whether EV71 evades the signal transduction phase regulated by a sophisticated interplay of cellular and viral proteins. Here, we show that EV71 inhibits anti-viral type I interferon (IFN) responses by targeting the mitochondrial anti-viral signaling (MAVS) protein—a unique adaptor molecule activated upon retinoic acid induced gene-I (RIG-I) and melanoma differentiation associated gene (MDA-5) viral recognition receptor signaling—upstream of type I interferon production. MAVS was cleaved and released from mitochondria during EV71 infection. An *in vitro* cleavage assay demonstrated that the viral 2A protease (2A^pro^), but not the mutant 2A^pro^ (2A^pro^-110) containing an inactivated catalytic site, cleaved MAVS. The Protease-Glo assay revealed that MAVS was cleaved at 3 residues between the proline-rich and transmembrane domains, and the resulting fragmentation effectively inactivated downstream signaling. In addition to MAVS cleavage, we found that EV71 infection also induced morphologic and functional changes to the mitochondria. The EV71 structural protein VP1 was detected on purified mitochondria, suggesting not only a novel role for mitochondria in the EV71 replication cycle but also an explanation of how EV71-derived 2A^pro^ could approach MAVS. Taken together, our findings reveal a novel strategy employed by EV71 to escape host anti-viral innate immunity that complements the known EV71-mediated immune-evasion mechanisms.

## Author Summary

Enterovirus 71 (EV71) is the causative pathogen of hand, foot, and mouth disease (HFMD). Since the 2008 outbreak of HFMD in Fuyang, Anhui province, China, HFMD has been a severe public health concern affecting children. The major obstacle hindering HFMD prevention and control efforts is the lack of targeted anti-viral treatments and preventive vaccines due to the poorly understood pathogenic mechanisms underlying EV71. Viral evasion of host innate immunity is thought to be a key factor in viral pathogenicity, and many viruses have evolved diverse antagonistic mechanisms during virus-host co-evolution. Here, we show that EV71 has evolved an effective mechanism to inhibit the signal transduction pathway leading to the production of type I interferon, which plays a central role in anti-viral innate immunity. This inhibition is carried out by an EV71-encoded 2A protease (2A^pro^) that cleaves MAVS—an adaptor molecule critical in the signaling pathway activated by the viral recognition receptors RIG-I and MDA-5—to escape host innate immunity. These findings provide new insights to understand EV71 pathogenesis.

## Introduction

When viruses infect host cells, the innate immune response is activated as the first line of defense against viral invasion. Pathogen associated molecular patterns (PAMPs) are sensed by host pattern recognition receptors (PRRs), resulting the expression of type I interferon and proinflammatory cytokines [1], [2]. These cytokines can induce an anti-viral state in the host cells and initiate host adaptive immunity, leading to limitation or clearance of the viral infection. Anti-viral innate immunity can be roughly divided into three phases: (i) the initiation phase, where PRRs recognize viral RNA and recruit specific signaling adaptor molecules; (ii) the signal-transduction phase, where adaptor molecules transduce signaling to activate IKK-related kinases that activate transcription factors, like interferon regulatory factor 3 (IRF3) and nuclear factor-κB (NF-κB); and (iii) the effector phase, where IRF3 and NF-κB translocate to the nucleus and prime type I IFN synthesis. Type I IFNs then activate the signal transducers and activators of transcription (STAT) pathway on neighboring cells to induce synthesis of interferon-stimulated genes (ISGs). RNA viruses are detected by membrane-bound Toll-like receptors (TLRs) and cytoplasmic sensors, including retinoic acid induced gene-I (RIG-I) and melanoma differentiation associated gene (MDA-5). Although RIG-I and MDA-5 are both RNA helicase domain-containing proteins that use mitochondrial anti-viral signaling protein (MAVS, also called VISA, IPS-1, Cardif) to transduce signaling, they specialize in sensing different types of viruses [3]–[6].

Enterovirus 71 (EV71), which belongs to the *Picornaviridae* family, is a single-stranded, positive-sense RNA virus. EV71 infection usually causes childhood exanthema, also known as hand, foot, and mouth disease (HFMD). Acute EV71 infection can also induce severe neurological disease, including aseptic meningitis, brainstem and/or cerebellar encephalitis, and acute flaccid paralysis [7]. EV71 outbreaks have been reported around the world since the first report in the United States in 1974 [8]. In recent years, the frequency and the severity of EV71 infection are increasing in China and pose a threat to human health and social stability. However, no effective vaccines or specific anti-viral treatments are currently available.

Although the specific molecular mechanism underlying EV71 pathogenesis is not clear, EV71 virulence is associated with circumventing anti-viral immunity. While type I IFN administration protects mice against EV71 infection, anti-IFNα/β neutralizing antibody treatment exacerbates EV71-induced disease [9]. Recent studies show that the EV71-encoded 3C protease (3C^pro^) inhibits the RIG-I and MAVS interaction and is able to cleave TIR domain-containing adaptor inducing IFN-β (TRIF), a key TLR3 adaptor molecule, to inhibit type I IFN production [10], [11]. Another recent study showed that 2A^pro^, another EV71 protease, reduced IFN receptor I (IFNAR1) expression that inhibited type I IFN signaling [12]. Although these known EV71-mediated inhibitory mechanisms affect the initiation and effector phases of the innate immune response, not much is known about the effect of EV71 infection on the signal transduction phase involving TLR3- or RIG-I/MDA5-mediated type I IFN production, a phase that is usually regulated by a sophisticated interplay between host and viral proteins under infection conditions. This study aimed to explore whether and how EV71 inhibits type I IFN production through regulating signal transduction pathways. We found that EV71 inhibited type I IFN responses upstream of IRF3 activation. MAVS, the common adaptor signaling molecule acting upstream of IRF3, was cleaved during EV71 infection. MAVS cleavage was independent of host cellular protease activity, but was dependent on EV71-encoded protease 2A^pro^, where 2A^pro^ cleaved MAVS at three residues with different degrees of cleavage. EV71 also induced morphological and functional changes to host-cell mitochondria, and the EV71 VP1 protein was found to associate with host-cell mitochondria. Overall, our findings reveal a novel virus–MAVS interaction that inhibits signal transduction induced by anti-viral innate immunity to evade the ensuing immune response.

## Results

### EV71 inhibits type I interferon responses upstream of IRF3 activation

**Figure 1:**
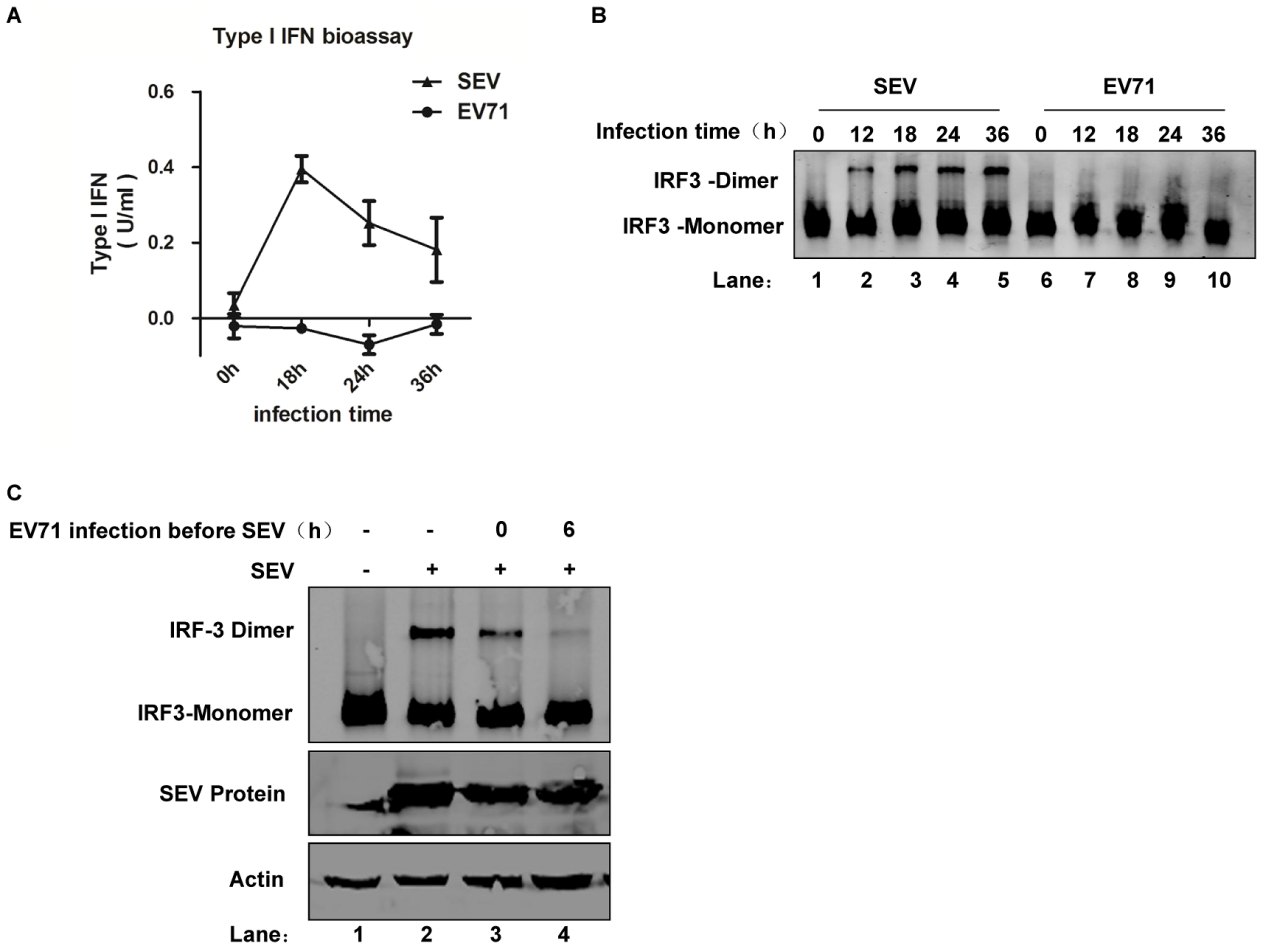
EV71 inhibits type I interferon responses upstream of IRF3 activation. (**A**) HeLa cells were infected with EV71 (multiplicity of infection [MOI] = 10) and SEV (20 HA/mL) for the indicated time. Supernatants were collected, and type I IFN production was measured by the type I IFN bioassay in the 2FTGH-ISRE cell line. Results were expressed as mean ± SD among triplicate samples. (**B**) HeLa cells were infected with SEV (20 HA/mL, lanes 1–5) and EV71 (MOI = 10, lanes 6–10) for indicated time. Cell lysates were subjected to native PAGE, and the presence of IRF3 monomers and dimers was detected by western blot. (**C**) HeLa cells were mock- or EV71-infected (MOI = 10) at the indicated time before SEV infection (lanes 3&4), and then the cells were super-infected with SEV (20 HA/mL) for 24 h (lanes 2–4); cells infected with SEV alone served as a positive control for IRF3 dimerization (lane 2). Cell extracts were subjected to IRF3 dimerization assay by native PAGE (upper panel). The same samples were resolved by SDS-PAGE followed by western blot analysis using a polyclonal antibody against Sendai virus (middle panel) and an antibody against actin (lower panel).

Previous studies demonstrated that EV71 evolved mechanisms to counteract type I IFN production [10]–[12]. To confirm and further clarify whether and how EV71 inhibits type I IFN production and determine at which step inhibition occurs, type I IFN production was evaluated. First, we measured type I IFN activity in supernatant from Sendai virus (SEV)- or EV71-infected HeLa cells using the type I IFN-responsive 2FTGH-ISRE reporter cell line. While the supernatant from the positive control SEV-infected HeLa cells exhibited time-dependent type I IFN production, supernatant from EV71 infected cells contained negligible type I IFN production over 36 h (Figure 1A). RT-PCR analysis showed that EV71 failed to induce mRNA expression of IFN-β or RANTES, a proinflammatory cytokine, in HeLa cells even though SEV could successfully do so (Supplemental Figure S1A). To confirm these results, a luciferase reporter assay was performed to investigate whether SEV- and EV71-infection induced IFN-β and NF-κB promoter activation. EV71 barely activated the IFN-β and NF-κB promoters (Supplemental Figure S1B). The above results suggest that EV71 inhibitory activity may occur upstream of the effector phase of type I IFN production.

Based on the above results, we next looked at IRF3 dimerization, which is a critical step upstream of IFN-β transcription and production. IRF3 dimerization was monitored by native PAGE, and we found that EV71-infected HeLa cells did not induce IRF3 dimerization even though SEV was able to induce it in a time-dependent manner (Figure 1B). This result indicates that EV71 might inhibit IFN-β production upstream of IRF3 activation. In order to confirm this result, native PAGE was performed on EV71-infected HeLa cells super-infected with SEV at different time points post-EV71 infection. The results showed that EV71 infection led to a pronounced, time-dependent decrease in SEV-induced IRF3 dimerization but did not interfere with SEV replication (Figure 1C). This EV71-mediated suppression of SEV-induced IRF3 dimerization reinforced the idea that EV71 inhibited IFN-β upstream of IRF3 activation.

### MAVS is cleaved in EV71-infected cells

**Figure 2:**
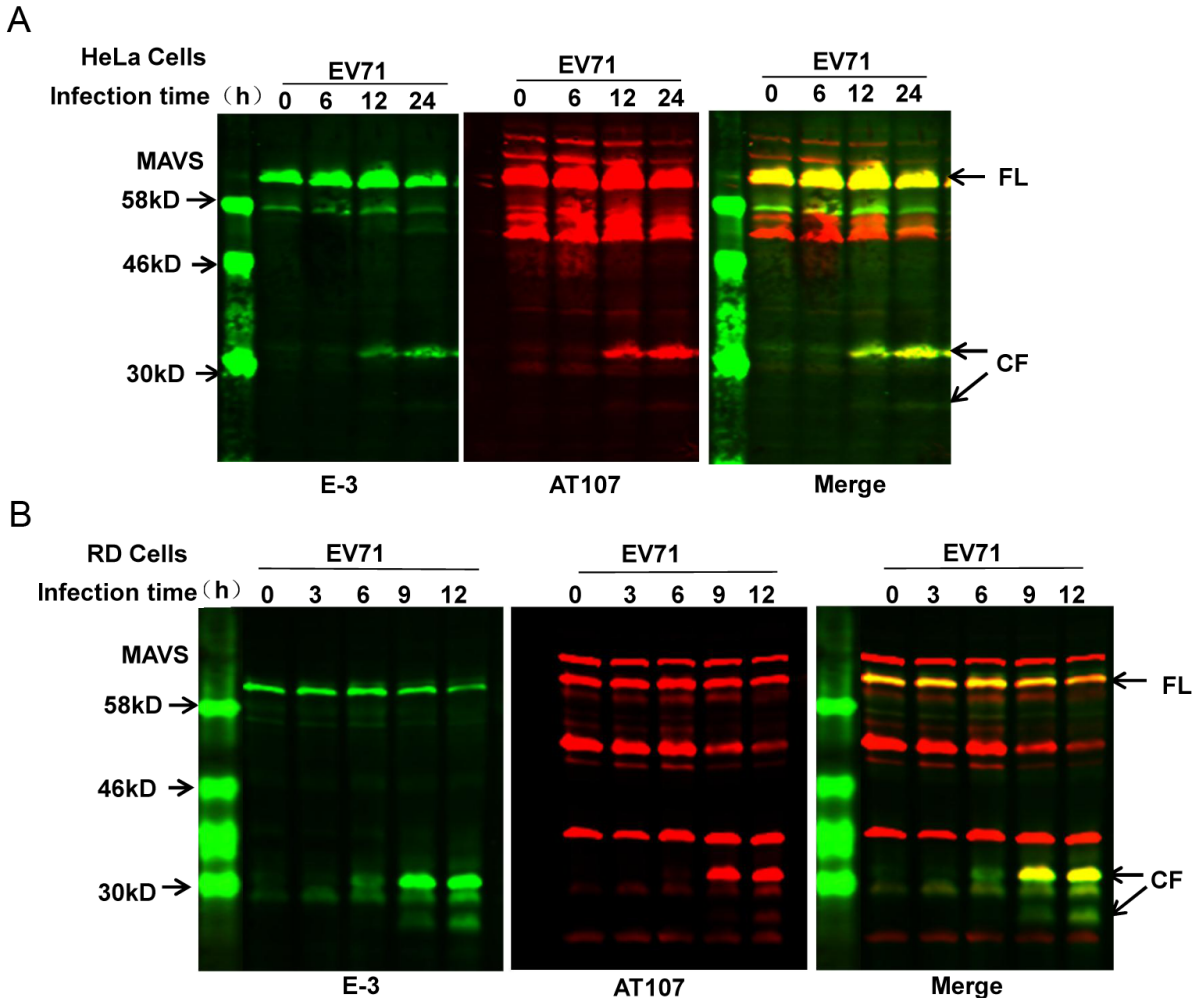
MAVS is cleaved in EV71-infected cells. Western blot analysis of MAVS expression in EV71-infected (MOI = 10) (**A**) HeLa cells and (**B**) RD cells for the indicated time. The time course evaluating MAVS expression was carried out by LI-COR Odyssey Dual-Color System using two different antibodies against MAVS (E-3, 700 nm, green; AT107, 800 nm, red). Results are displayed as images from each channel as well as an overlaid image of the two channels. Arrows indicate full-length MAVS (FL) and the cleaved fragments (CF) derived from MAVS.

MAVS is the unique adaptor molecule shared between the RIG-I and MDA-5 cytoplasmic PRRs, which acts upstream of IRF3 [3]–[6]. Many viruses, such as hepatitis C virus (HCV) [6], [13]–[16], GB virus [17], hepatitis A virus (HAV) [18], Coxsackievirus B3 (CVB3) [19], and rhinovirus [20], specifically target MAVS in order to escape host innate immunity. Considering the important function of MAVS in both the RIG-I and MDA-5 signaling pathway, a time-course study was conducted to test MAVS expression levels during EV71 infection by western blot. We found that expression of full-length MAVS declined after EV71 infection, and two fragments appeared at approximately 30 kD in both EV71-infected HeLa cells and rhabdomyosarcoma (RD) cells (Figure 2A–B). This result suggested that MAVS was cleaved during EV71 infection and that more than one cleavage residue may exist. In order to confirm that MAVS was indeed the source of these cleavage bands, two separate antibodies raised against different amino acid sequences of MAVS (E-3 was raised against residues 1–135 of human MAVS, while AT107 was raised against residues 160–450) were used to probe the above-mentioned western blot. Indeed, the cleavage products were recognized by both antibodies, as exhibited by the yellow signal that appeared after merging the green (E3) and red (AT107) western blot images. This result confirmed that MAVS was the source of the cleaved products (Figure 2A–B).

**Figure 3:**
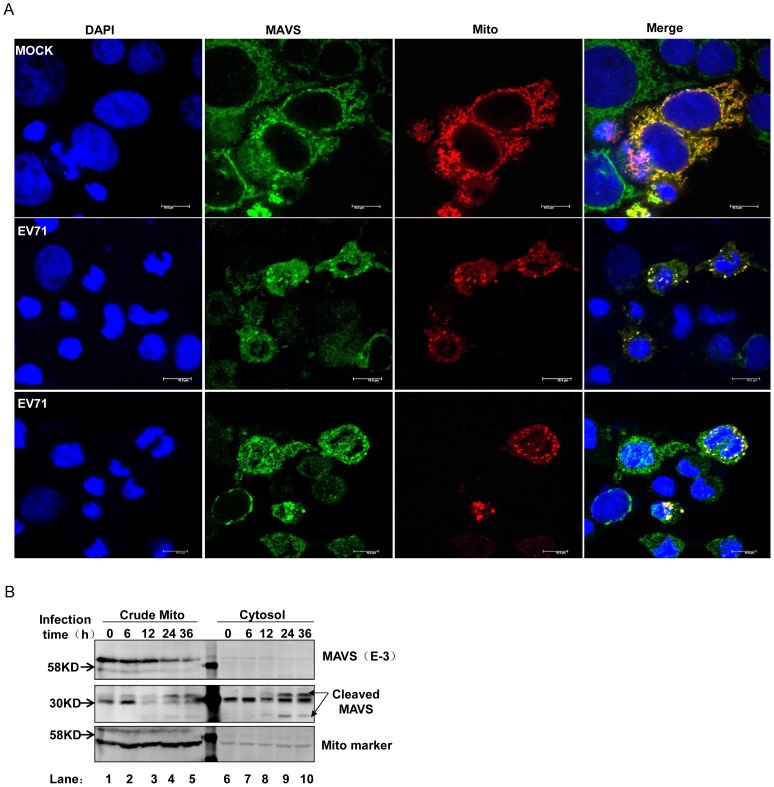
MAVS is cleaved from mitochondria. (**A**) HeLa cells were transfected with Mito-dsRed plasmid. At 24 h after transfection, cells were mock infected (upper panel) or infected with EV71 (MOI = 10, middle and lower panels). Cells were then fixed, stained for MAVS using MAVS E-3 antibody at the 24 h post-infection time point, and confocal microscopy analysis was performed (nucleus: blue; MAVS: green; mitochondria: red. Scale bar: 10 µm.). All images represent 3 independent experiments. (**B**) EV71-infected HeLa cells were subjected to differential centrifugation to separate subcellular fractions. Western blot analysis was carried out in the crude mitochondrial (lanes 1–5) and cytosolic (lanes 6–10) compartments to detect full-length MAVS (upper panel) and cleaved MAVS (middle panel) using the anti-MAVS E-3 antibody. Mito marker was used to demonstrate isolation efficiency (lower panel).

MAVS is localized on the outer membrane of mitochondria, and this sub-cellular localization is crucial for its function in anti-viral signaling. We therefore examined whether any changes to the cellular distribution of its cleavage products occurred during EV71 infection by confocal microscopy. The results showed that MAVS co-localized with Mito-dsRed, an RFP-containing mitochondrial target construct, in mock-infected cells. However, EV71 infection dramatically disrupted this co-localization (Figure 3A). To further confirm this, we separated the mitochondrial protein from the cytosolic protein by differential centrifugation. Western blot analysis was performed to determine the distribution of MAVS and its cleaved fragments; we clearly observed that MAVS was cleaved from the mitochondria, and the cleaved fragments were released into the cytoplasm (Figure 3B).

### MAVS cleavage is independent of cellular apoptosis and proteasome degradation

**Figure 4:**
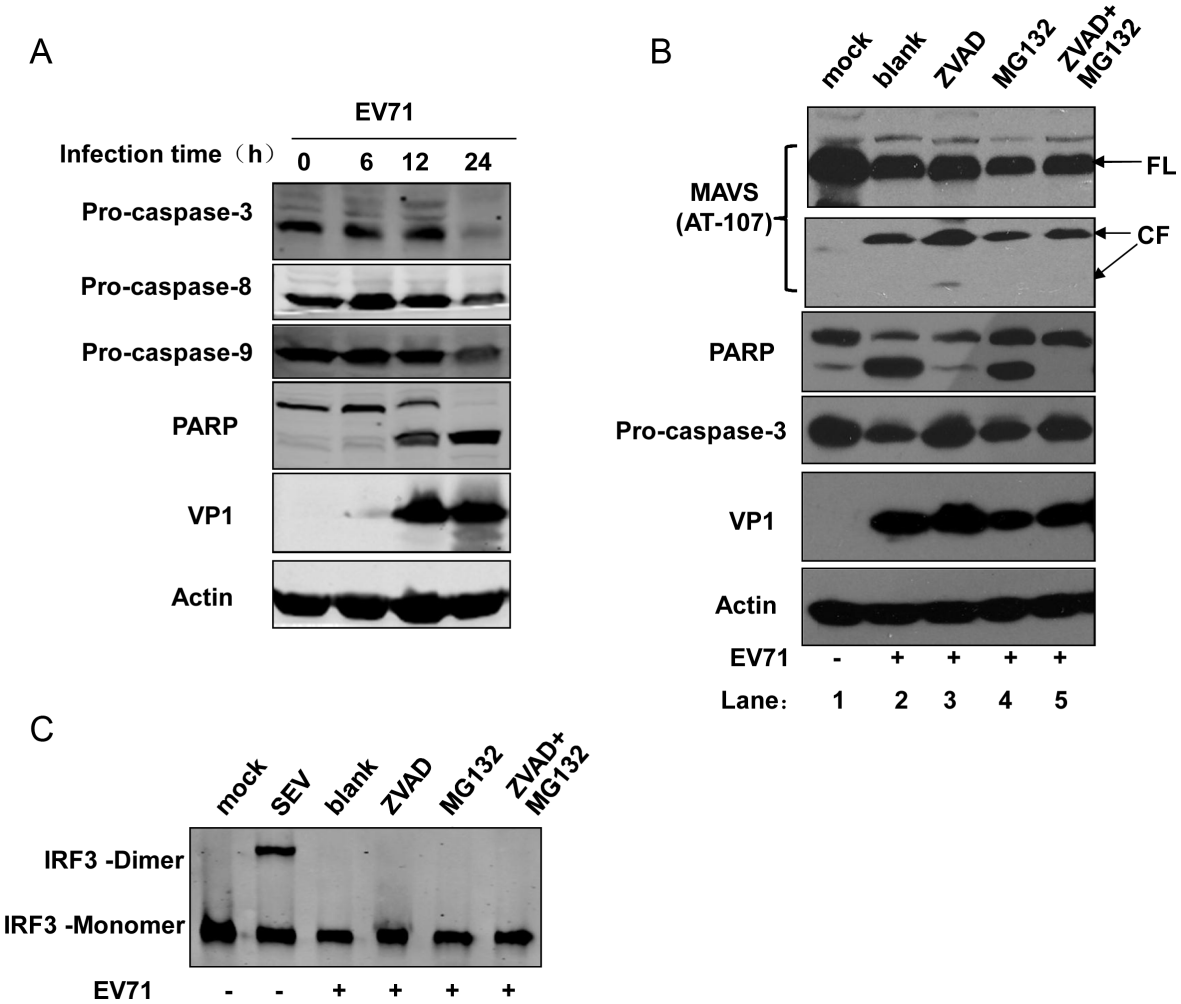
MAVS cleavage is independent of cellular apoptosis and proteasome degradation. (**A**) Time course evaluation of caspase 3, 8, and 9 activation and PARP cleavage in EV71-infected HeLa cells (MOI = 10). (**B**) Effects of caspase and proteasome inhibitors on EV71 induced MAVS cleavage. HeLa cells were mock infected (lane 1) or infected with EV71 (MOI = 10) in the absence (blank, lane 2) or presence of Z-VAD-FMK (ZVAD) (100 µM, lane 3), MG132 (20 µM, lane 4), or both (lane 5). At the 24 h post-infection time point, the cells were harvested and western blot was used to detect MAVS and its cleavage fragments using the anti-MAVS AT107 antibody. (**C**) Effects of caspase and proteasome inhibitors on IRF3 dimerization. The inhibitor concentrations used in this experiment were the same as in (B).

Viral infection induces cellular apoptosis as a consequence of the battle between the host cells and the virus. Apoptosis has been observed to occur in EV71-infected cells [21]–[23], and the EV71-derived proteases 2A^pro^ and 3C^pro^ have been reported to induce this process [24], [25]. During virus-induced apoptosis, caspases are activated and lead to cleavage of some cellular proteins like PARP. Innate immune signaling proteins such as RIG-I, MDA-5, and MAVS are also targeted by activated caspases in other viral infections [20], [26]–[28]. These proteins also undergo proteasomal degradation through host- and viral-protein-mediated ubiquitin-ligating proteins, like host-derived RNF125, RNF5, and PCBP2 and the virus-derived hepatitis B virus (HBV) X protein [29]–[32]. To test whether EV71-induced MAVS cleavage is associated with cellular apoptosis and activated caspases, we first examined whether caspase activation occurred after EV71 infection in HeLa cells by western blot analysis of pro-caspase 3, 8, 9, PARP, and EV71-VP1 during an infection time course. EV71 infection led to caspase 3, 8, and 9 activation as well as PARP cleavage. PARP cleavage began at 12 h post-infection and was nearly complete at 24 h (Figure 4A), while MAVS cleavage was similarly detected at both 12 and 24 h post-infection (Figure 2A), suggesting that MAVS cleavage accompanied cellular apoptosis. To further investigate whether MAVS cleavage is the result of activated caspases or proteasome degradation, we tested the effect of pan-caspase inhibitor Z-VAD-FMK and proteasome inhibitor MG132 on MAVS cleavage in mock- or EV71-infected HeLa cells. Western blot analysis showed that PARP cleavage and caspase-3 activation, but not MAVS cleavage, was inhibited by Z-VAD-FMK alone or Z-VAD-FMK in combination with MG132. MG132 alone inhibited EV71 replication (indicated by the decreased VP1 protein, which was also reported in other viral infections [33]–[35]), but could not rescue MAVS cleavage (Figure 4B). Consistent with these results, neither the inhibitors alone nor their combined treatment could rescue IRF3 dimerization in EV71-infected cells as determined by native PAGE (Figure 4C). Taken together, the above results indicate that MAVS cleavage is independent of cellular apoptosis and proteasome degradation.

### EV71 induces mitochondrial abnormalities, and EV1-derived VP1 protein appears on mitochondria

**Figure 5:**
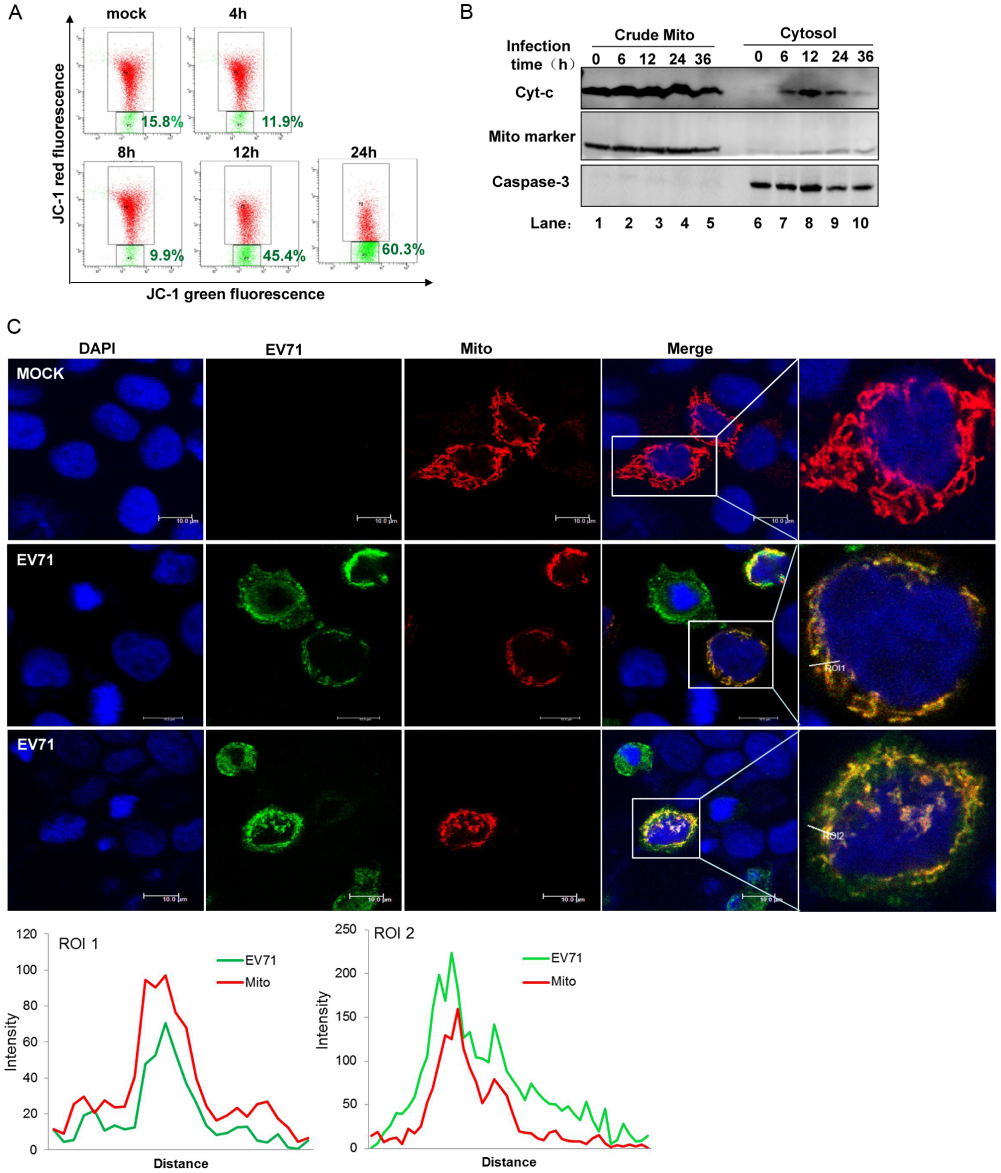
EV71 targets mitochondria to inhibit innate immunity. HeLa cells were infected with EV71 (MOI = 10) for the indicated time. (**A**) Mitochondrial membrane potential was analyzed by flow cytometry method using 5,5′,6,6′-tetrachloro-1,1′,3,3′-tetramethyl benzimidazolyl carbocyanine iodide (JC-1). The vertical and horizontal coordinates represented JC-1 red and green fluorescence, respectively. (**B**) Cells were subjected to differential centrifugation to separate the crude mitochondrial (lanes 1–5) from the cytosolic (lanes 6–10) compartment. Western blot analysis was performed on these two compartments to detect cytochrome c (cyt-c) (upper panel). Mito marker (middle panel) and caspase 3 (lower panel) served as the mitochondrial and cytosolic markers, respectively [64]. (**C**) HeLa cells were transfected with Mito-dsRed plasmid. At 24 h post-transfection, cells were infected with EV71 (MOI = 10, middle and lower panel). At 24 h post-infection, cells were fixed and stained for EV71 using an antibody against EV71. Morphological changes in the mitochondria were analyzed under confocal microscopy (nucleus: blue; EV71: green; mitochondria: red; Scale bar: 10 µm). Histograms show the fluorescence intensity analysis results from ROI1 and ROI2 in the merged panel. All images represent 3 independent experiments.

Mitochondria are well known for their crucial role in energy production, calcium homeostasis, and apoptosis. The presence of MAVS on the mitochondrial outer membrane indicates that this organelle has anti-viral functions. Recently, reports show that mitochondrial dynamics and membrane potential (ΔΨ m) are all required for MAVS-mediated anti-viral signaling, which underscores the importance of the mitochondrial microenvironment in anti-viral signaling [36], [37]. As MAVS was cleaved during EV71 infection and accompanied cellular apoptosis, we evaluated whether other mitochondrial abnormalities were associated with EV71 infection. First, we measured membrane potential using Mito-probe JC-1, a cationic dye that indicates mitochondrial depolarization by red-green fluorescence ratio reduction. Upon EV71 infection, an obvious loss of ΔΨ m began at 12 h (Figure 5A). We next assessed mitochondrial outer-membrane permeability by measuring cytochrome c release, another indicator of mitochondrial abnormality, and found that EV71 infection led to a small amount of cytochrome c release from the mitochondria into the cytoplasm (Figure 5B). To further explore mitochondrial abnormalities, we observed morphological changes by confocal microscopy of Mito-dsRed-transfected HeLa cells infected with EV71. Dramatic morphological changes occurred, as the typical mitochondrial network structure observed in mock-infected cells became diffuse and unclear in EV71 infected cells. Moreover, mitochondria partially stained positive for an anti-EV71 virus antibody, indicating viral co-localization with mitochondria (Figure 5C). Further in-cell western blot analysis demonstrated that the EV71 antibody was against the EV71 structural protein VP2 (Supplemental Figure S2). The extent of this partial co-localization indicated that mitochondria might only function at particular steps during the viral life cycle.

**Figure 6:**
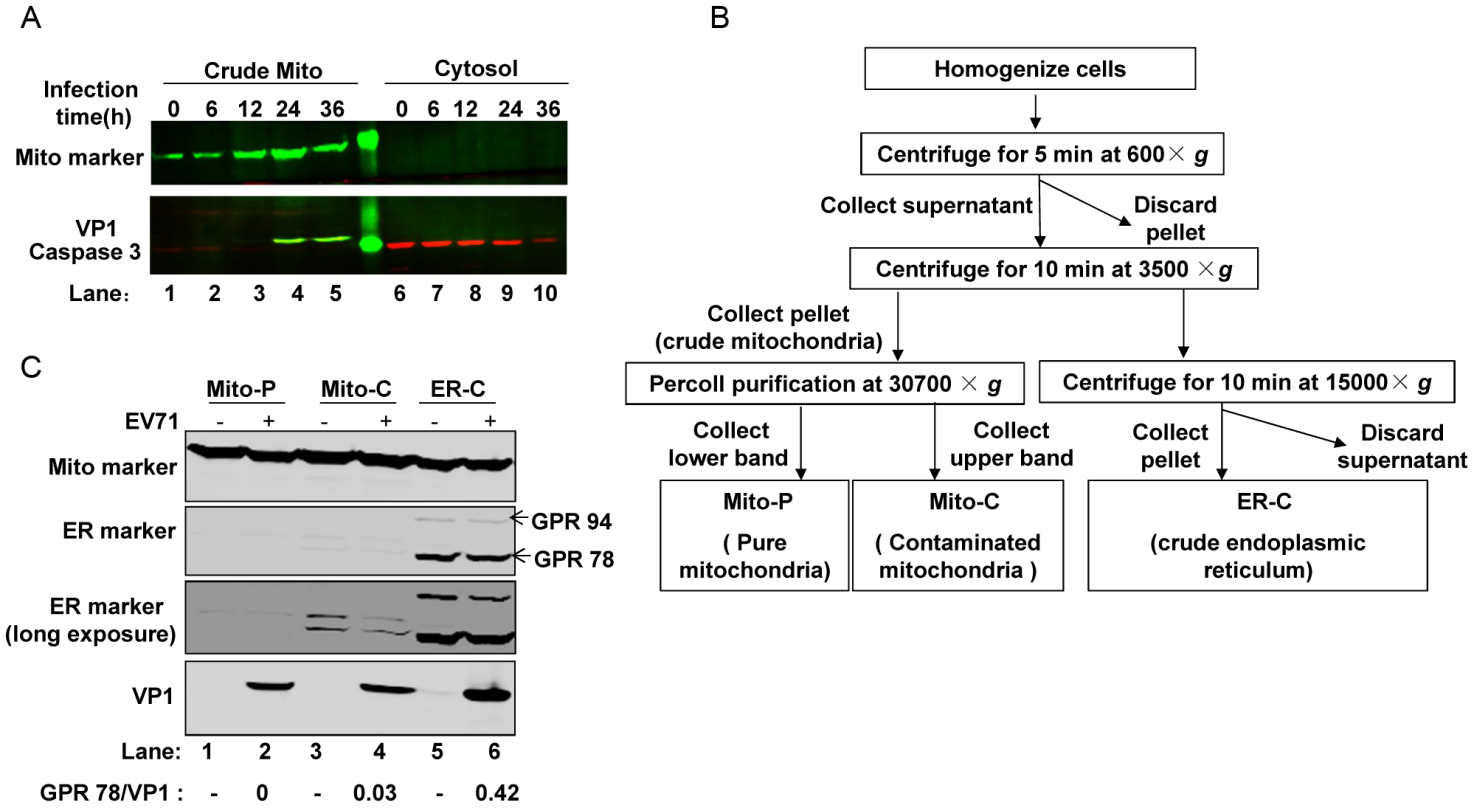
EV71 viral protein co-localizes with mitochondria. (**A**) Western blot analysis for EV71 VP1 protein in the crude mitochondrial (lanes 1–5) and cytosolic (lanes 6–10) compartments from fractionated EV71-infected HeLa cells at different time points (lower panel, green). Mito marker (upper panel) and cytosolic marker caspase 3 (lower panel, red) demonstrated fractionation efficiency. (**B**) Schematic diagram detailing the mitochondria isolation and purification procedure for (F). (**C**) Western blot analysis of EV71 VP1 protein in pure mitochondria (Mito-P, lanes 1&2), contaminated mitochondria (Mito-C, lanes 3&4), and crude ER (ER-C, lanes 5&6) from mock- or EV71-infected (MOI = 10) HeLa cells. Mitochondria isolation and purification procedure was carried out at 24 h post-infection. ER marker and Mito marker demonstrated isolation and purification efficiency. The ratio of ER marker (GPR78)/VP1 from each preparation was calculated and listed below the graph.

The processed viral components of many viruses, like HBx of HBV [38], NS3/4A and NS4A of HCV [6], [13], [14], [16], [39], 2B of poliovirus [40], and the 3ABC precursor of HAV [18], have been reported to associate with mitochondria to induce morphologic and functional changes in the mitochondria, causing subsequent apoptosis or targeting MAVS to inhibit innate-immune signaling. Based on the above analysis, we tested whether mitochondria were involved in the EV71 viral replication cycle by evaluating whether the EV71 structural protein, VP1, physically associated with mitochondria. Western blot analysis of mitochondria isolated from the cytoplasmic protein fraction showed that VP1 is mainly detected in the crude mitochondria as compared to the cytosol compartment (Figure 6A). In order to exclude the possibility that the VP1 detected in the isolated mitochondria fraction was a result of endoplasmic reticulum (ER) contamination that is believed to be important for picornavirus replication, we performed a more rigorous protocol to isolate mitochondria (using slower centrifugation speeds) and further purified it by Percoll gradient fractionation (Figure 6B) [15], [41]. Using specific markers for ER and mitochondria, western blot analysis demonstrated that the pure mitochondria were not contaminated with ER and that EV71 VP1 still associated with the mitochondrial compartment (Figure 6C). Collectively, the above results strongly indicate that the EV71 viral replication cycle involves the mitochondria, suggesting that viral proteins expressed during EV71 propagation may cause mitochondrial abnormalities and induce MAVS cleavage.

### EV71 2A^pro^ cleaves MAVS

**Figure 7:**
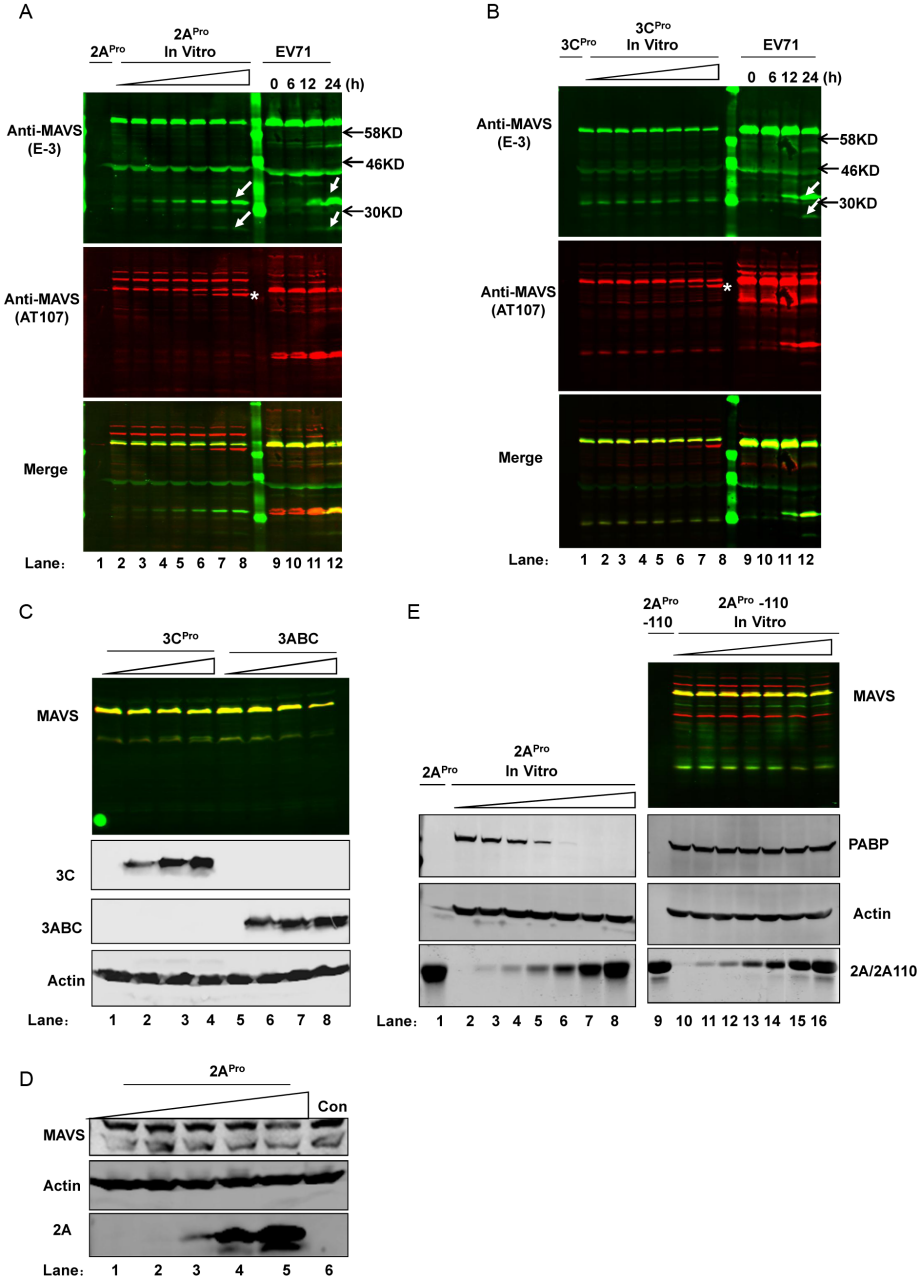
EV71 2A^pro^ cleaves MAVS. (**A, B**) *In vitro* dose- dependent cleavage assay of EV71 2A^pro^ (A) and 3C^pro^ (B) on MAVS using LI-COR Odyssey Dual-Color System. Increasing doses of recombinant proteases were added to the cell lysates and incubated at 37°C for 6 h (from 0–200 ng/µL, lanes 2–8); recombinant proteases (lane 1) and EV71-infected HeLa cells (lanes 9–12) served as negative and positive controls, respectively. Two antibodies recognizing different MAVS epitopes were used (E-3, 700 nm, green; AT107, 800 nm, red). An overlay of the two channels is shown in the “Merge” panel. White arrows indicate cleaved bands in EV71-infected HeLa cells and the same-size bands in 2A^pro^-cleaved HeLa extracts. (**C**) Western blot analysis of MAVS in HeLa cells transfected with increasing doses of plasmids (0–4 µg) encoding EV71 3C^pro^ (lanes 1–4) and 3ABC (lanes 5–8) precursor protein fused with GFP. The MAVS image is an overlay of two signals from the different channels described in (A). The same cell lysates were also used to detect 3C^pro^ and 3ABC using an antibody against GFP; actin served as the loading control. (**D**) Western blot analysis of MAVS in BSRT7/5 cells transfected with increasing doses of pcDNA3.1-IRES-2A plasmid (lanes 1–5, 0–4 µg) and pcDNA3.1-EGFP control plasmid (4 µg). (**E**) *In vitro* dosage cleavage assay (0–200 ng/µL) of 2A^pro^ (lanes 2–8) and mutated 2A^pro^ (2A^pro^-110) (lanes 10–16) on MAVS; recombinant 2A^pro^ (lane 1) and 2A^pro^-110 (lane 9) served as negative controls. The MAVS image is an overlay of two signals from the different channels described in (A). PABP was used as a readout for the enzyme activity of 2A^pro^ and 2A^pro^-110; actin served as a loading control. The image of 2A^pro^ and 2A^pro^-110 (lower panel) is a direct scan of SDS-PAGE gel stained with Coomassie brilliant blue.

EV71 encodes two proteases, 2A^pro^ and 3C^pro^, that are important for processing viral protein precursors; they also reportedly cleave a variety of host-cell molecules that affect fundamental functions of the host cell. Since we found that MAVS was cleaved upon EV71 infection, we speculated that EV71 proteins executed this cleavage, especially as we previously excluded the role of cellular proteases and further detected the presence of viral protein on mitochondria. Since 2A^pro^ has a strong inhibitory effect on host gene expression that makes it difficult to express and test in cultured cells, we first took advantage of a cell-free *in vitro* cleavage system—considered to be the most straight-forward approach to study picornavirus protease hydrolysis function [42]–[48]—to determine whether EV71-encoded 2A^pro^ and 3C^pro^ proteases could directly cleave MAVS. We incubated recombinant EV71 2A^pro^ and 3C^pro^ with HeLa cell extracts and detected MAVS cleavage by western blot using two antibodies that recognize different MAVS epitopes. EV71-infected HeLa cells were used as the positive control. We found that although both proteases generated cleavage bands, only 2A^pro^ generated the same-sized cleavage bands as the EV71-infected cells. The appearance of these cleavage bands, approximately 30 kD in size, correlated with 2A^pro^ treatment in a dose-dependent manner (Figure 7A–B). Another band in both 2A^pro^ and 3C^pro^ treated cell extracts (Figure 7A–B, indicated by *) was considered to be a non-specific cleavage product and will be discussed later. In order to further scrutinize the role of EV71 3C^pro^, we transfected HeLa cells with increasing doses of a plasmid encoding GFP-tagged 3C^pro^ and 3ABC proteases, as HAV use the 3ABC precursor to cleave MAVS [18]. Neither of these proteins induced MAVS cleavage even when expressed at a high level in HeLa cells (Figure 7C). This result was also consistent with our previous study showing that EV71 3C^pro^ could not interact with MAVS when over-expressed in live cells [11].

We next explored whether 2A^pro^ exhibited any proteolysis ability on MAVS by transfecting a 2A^pro^-expressing plasmid into HeLa cells. eIF4GI, a known substrate of 2A^pro^, was used as an readout to indicate whether 2A^pro^ was functional in this experimental system, as we know that 2A^pro^ expression in this system may be weak since 2A^pro^ protein was difficult to detect by western blot (likely due to the concomitant restriction on its own expression from its inhibition effect on host gene expression). To our surprise, while eIF4GI cleavage was detected in this system, PABP, another 2A^pro^ substrate [20], [26], and MAVS remained intact (Supplemental Figure S3A). We speculated that this difference might be due to the varied sensitivities that these substrates have to 2A^pro^ levels, and we tested this idea by a time-course study in EV71-infected cells. Since all mature EV71 viral proteins are derived from the same poly-protein precursor that undergoes subsequent post-translational cleavage, the amount of VP1 could indirectly reflect the varied expression of 2A^pro^ and was therefore utilized to monitor 2A^pro^ expression in this study. The results showed that eIF4GI cleavage appeared at 6 h after EV71 infection when VP1 protein was expressed at a low, not detectable level; in contrast, PABP and MAVS cleavage was observed at a later time point, at 12 h, when VP1 was abundantly expressed during infection (Supplemental Figure S3B). This result supported our above speculation. Previous attempts to efficiently express target genes in mammalian cells used the prokaryotic T7 RNA polymerase and the internal ribosome entry site sequence (IRES) of encephalomyocarditis virus (EMCV) to avoid host transcription factors and permit mRNA translation in a capping-independent way [49]. Another study showed that the foot-and-mouth disease virus (FMDV), which also belongs to the *Picornaviridae* family, could be efficiently rescued in a baby hamster kidney cell line (BHK-21) stably expressing T7 polymerase [50]. Considering that FMDV has a similar genomic structure and encodes a similar protease to EV71 2A^pro^ [45], [51] and that our 2A^pro^-expressing plasmid contained both a T7 promoter and IRES sequence upstream of the 2A^pro^ coding region, we exogenously expressed 2A^pro^ and assessed its cleavage effect on MAVS in BSRT7/5 cells, a derivative cell line from BHK-21 that constitutively expresses T7 RNA polymerase [52]. 2A^pro^ was indeed abundantly expressed in these cells, and the results showed that MAVS decreased with increasing 2A^pro^ expression (Figure 7D). However, the cleavage bands were absent in this system; this absence might be due to the amino acid sequence differences between human and hamster MAVS, or to highly efficient cleavage in this over-expression system, rendering the cleavage fragments unstable or short-lived. An analogous phenomenon was previously reported in CVB 3C^pro^-mediated cleavage of MAVS and in HCV-mediated cleavage of TRIF [19]. Taken together, these results suggest that EV71 2A^pro^, but not 3C^pro^, is the protease inducing MAVS cleavage upon EV71 infection.

As EV71 2A^pro^ is a cysteine protease, its major catalytic sites are His21, Asp39, and Cys110. To further confirm that the catalytic enzymatic activity of 2A^pro^ is responsible for cleaving MAVS, we introduced a mutation in 2A^pro^ that changed amino acid 110 from Cys to Ala (named 2A^pro^-110), which destroyed and inactivated the catalytic site of 2A^pro^ [12], [47], [53]. We incubated 2A^pro^-110 with HeLa cell extracts and used PABP as a positive control for picornavirus 2A^pro^ enzyme activity. Western blot results showed that the mutated 2A^pro^ lost the ability to induce cleavage of both MAVS and PABP in this cell-free cleavage system (Figure 7E). Taken together, these results suggest that EV71 2A^pro^ mediates MAVS cleavage during EV71 infection, and that the catalytic enzyme activity of 2A^pro^ is required for cleaving the MAVS protein.

### EV71 2A^pro^ cleaves MAVS on multiple distinct sites

**Figure 8:**
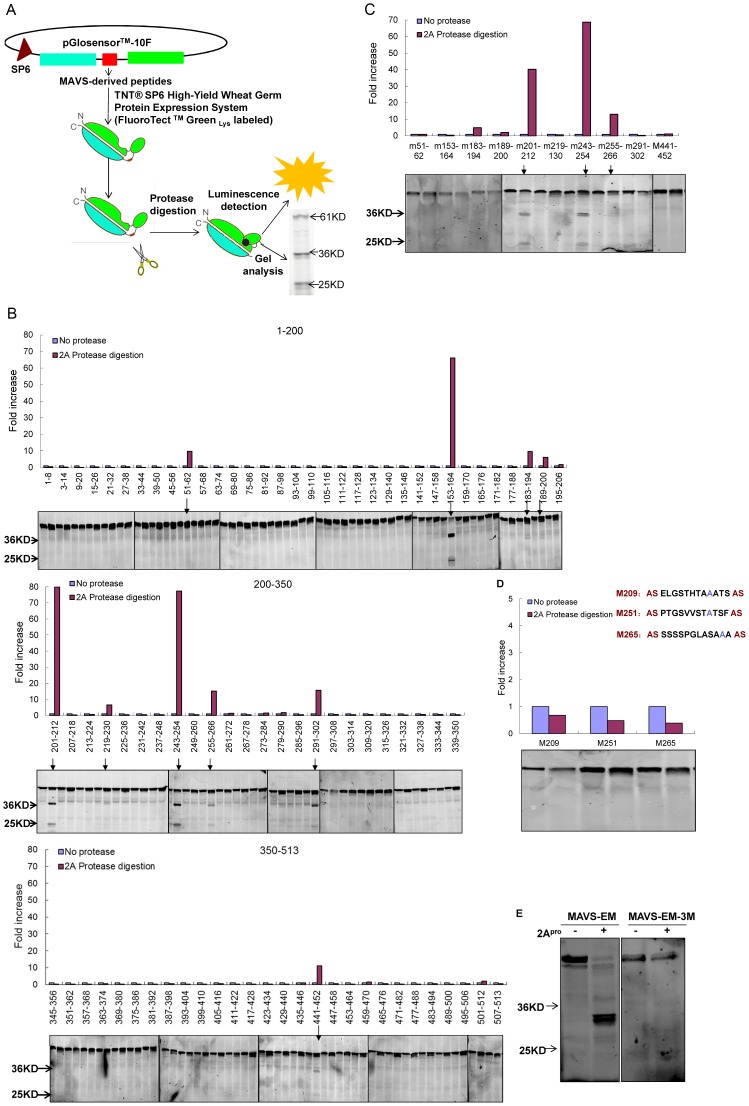
EV71 2A^pro^ cleaves MAVS at multiple residues. (**A**) Schematic diagram of the Protease-Glo assay of 2A^pro^ on MAVS extra-membrane region. (**B**) The first-round screening of 86 constructs containing the coding region for the 12-mer polypeptides covering the MAVS extra-membrane region. Luciferase assay results are shown together with gel analysis results. (**C**) The second-round screening of the 10 positive constructs selected in the first round of screening (Table 1) using a mutated pGlosensor-10F vector containing a mutation from Gly to Ala in the linker region. (**D**) Protease-Glo assay of M209, M251, and M265. These constructs were mutated from the 3 selected constructs from the second round of screening (Table 2), and each contains a point mutation (from Gly to Ala) at residue 209, 251, or 265. (**E**) *In vitro* cleavage assay of EV71 2A^pro^ on *in vitro* translated extra-membrane region of MAVS (MAVS-EM) and its mutants (MAVS-EM-3M) labeled with FluoroTect Green_Lys_ by gel analysis.

In order to identify the 2A^pro^-targeted cleavage residue(s) within the MAVS protein, we took advantage of the Protease-Glo Assay system to screen the whole extra-membrane region of MAVS. In this system, synthesized oligonucleotides encoding 12-mer polypeptides of MAVS (every 12 amino acids, with 6 amino-acid overlap) were inserted in-frame into a pGlosensor-10F linear vector that contained a genetically engineered firefly luciferase. The constructs were then expressed in a protein expression system labeled with FluoroTect Green_Lys_ and used as substrate for 2A^pro^. If 2A^pro^ cleaved any of the expressed polypeptides, an increase in luciferase activity would be detected, and two cleaved products at 36 and 25 kD would emerge in gel analysis (Figure 8A). Upon the first round of screening the 86 constructs we generated, we chose any plasmid exhibiting more than a 5-fold increase in luminescence density together with the visualized cleavage products in gel analysis as positive candidates; 10 constructs met this criterion (Figure 8B, Table 1). Some of these positive constructs may be false positives, since the linker region of pGlosensor-10F vector contains a Gly residue that is prone to being recognized as P1′ site of 2A^pro^ substrate and cleaved by 2A^pro^ [47], [54]–[56]. The Gly residues were mutated to Ala, and these vectors were used in the second round of screening. Three constructs remained positive and were found to encode MAVS protein residues 201–212, 243–254, and 255–266 (Figure 8C; Table 2).

**Table 1:** Constructs identified in the first round of screening by Protease-Glo assay.

NO.	Residues	Sequences	Fold increase
1	51–62	**AS** NRDTLWHLFNTL **GS**	9.7
2	153–164	**AS** PGENSEQALQTL **GS**	66.08
3	183–194	**AS** DLAALSPLTSSG **GS**	9.55
4	189–200	**AS** PLTSSGHQEQDT **GS**	6.06
5	201–212	**AS** ELGSTHTAGATS **GS**	79.86
6	219–130	**AS** GPVSPSVSFQPL **GS**	6.47
7	243–254	**AS** PTGSVVSTGTSF **GS**	77.28
8	255–266	**AS** SSSSPGLASAGA **GS**	15.15
9	291–302	**AS** ANSLPSKVPTTL **GS**	15.65
10	441–452	**AS** ISASTSLGMGPC **GS**	11.19

Note: Bold characters represent the linker region of pGlosensor-10F vector.

**Table 2:** Constructs identified in the second round of screening by Protease-Glo assay.

NO.	Residues	Sequence	Fold increase
5	201–212	**AS** ELGSTHTAGATS **AS**	40
7	243–254	**AS** PTGSVVSTGTSF **AS**	68
8	255–266	**AS** SSSSPGLASAGA **AS**	13

Note: Bold characters represent the linker region of pGlosensor-10F vector.

Some characteristics are common among picornavirus 2A^pro^ substrates, according to previous studies: the P1 position is preferentially occupied with a hydrophobic residue, and the P2 position is usually a Thr/Ser residue [47], [54]–[56]. The amino acid composition of the three positive polypeptides revealed that the P1′ residues were composed of Gly209, Gly251, and Gly265, respectively. We therefore constructed site-directed mutants (from Gly to Ala) of these potential cleavage residues and designated them as M209, M251, and M265. Upon exposure to 2A^pro^, the results showed that these mutations conferred resistance to all three constructs (Figure 8D). To further confirm the above results, we constructed plasmids encoding the non-mutated MAVS extra-membrane segment (designated as MAVS-EM) as well as a corresponding MAVS mutant containing Gly to Ala mutations at all three (209, 251, and 265) sites (designated as MAVS-EM-3M). These two plasmids were expressed by *in vitro* translation in the presence of FluoroTect Green_Lys_. Gel analysis of the 2A^pro^-induced cleavage pattern demonstrated that 2A^pro^ hydrolyzed MAVS-EM but failed to hydrolyze MAVS-EM-3M (Figure 8E). Taken together, these results demonstrate that Gly209, Gly251, and Gly265 are the cleavage residues within MAVS that are targeted by EV71 2A^pro^.

We also tested EV71 3C^pro^ in both the Protease-Glo assay screening for cleavage sites in the extra-membrane region of MAVS and in the cleavage assay testing cleavage ability on the *in vitro* translated MAVS-EM. Although two oligo sets (encoding MAVS residues 87–98 and 147–158) appeared to be positive candidates in the Protease-Glo assay (Supplemental Figure S4), 3C^pro^ failed to cleave *in vitro* translated MAVS-EM (Supplemental Figure S5). This result was consistent with the results obtained from the 3C^pro^ and 3ABC over-expressed cells, and again demonstrated the inability of EV71 3C^pro^ to cleave MAVS. The discrepancy between the results may be explained by MAVS harboring potential 3C^pro^ cleavage sites that could be cleaved in the linear-polypeptide-based screening assay but not in the whole-protein-based cleavage assay due to conformational structure constraints that might block the approaching of 3C^pro^ protein.

### EV71 2A^pro^ exhibits different proteolysis activity on the cleavage residues in MAVS

**Figure 9:**
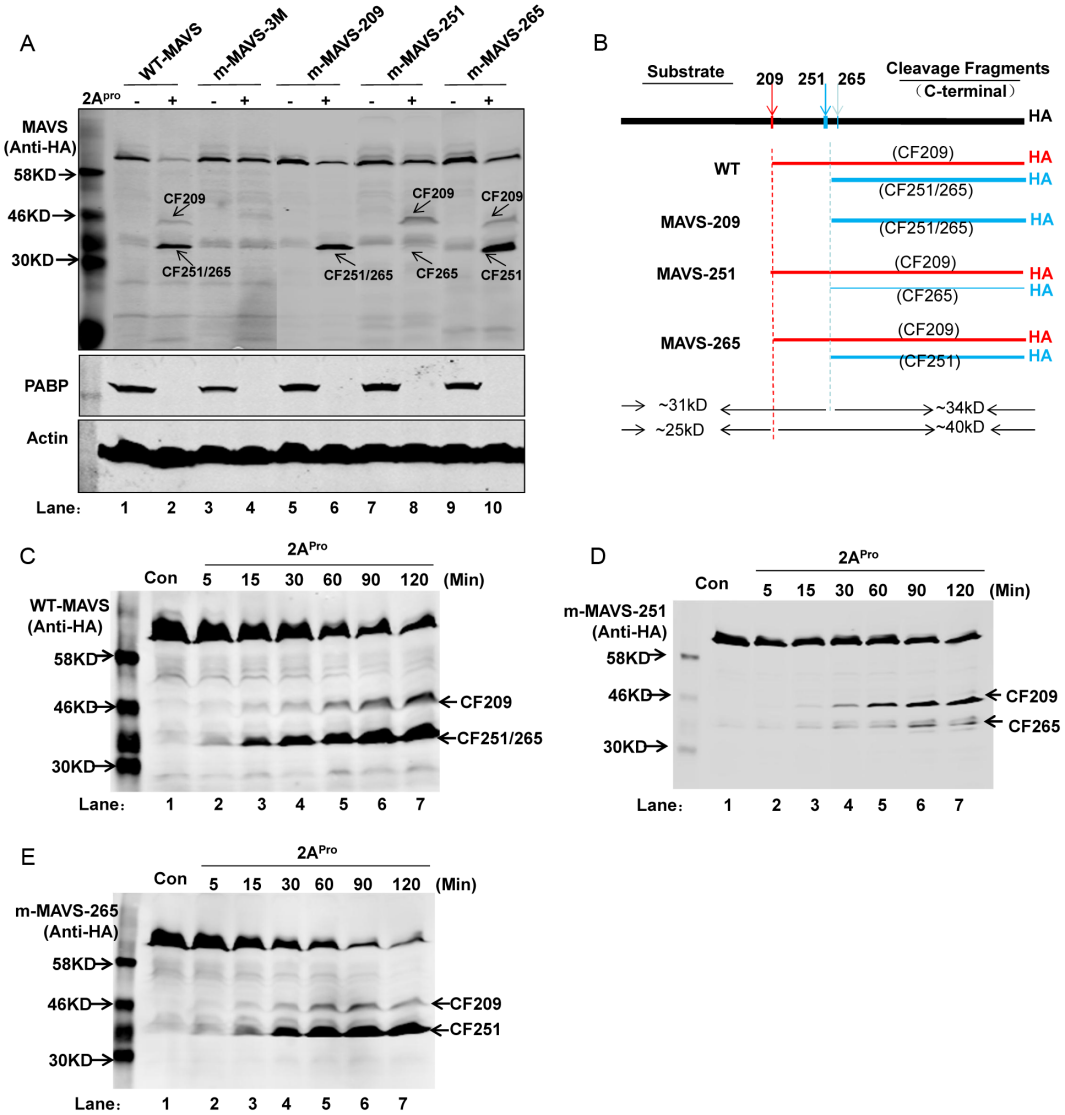
*In vitro* cleavage assay of EV71 2A^pro^ on MAVS and MAVS mutants expressed in HeLa cells. (**A**) The cell lysates from stable cell lines expressing WT-MAVS (lanes 1&2), m-MAVS-3M (lanes 3&4), m-MAVS-209 (lanes 5&6), m-MAVS-251 (lanes 7&8), and m-MAVS-265 (lanes 9&10) were incubated with 200 ng/µL 2A^pro^ at 30°C for 2 h; the cell lysates were then subjected to western blot analysis to probe MAVS using an HA antibody against an HA peptide fused to the C-terminus of MAVS and MAVS mutants. Cleavage of PABP served as a readout for the enzymatic activity of 2A^pro^. (**B**) Schematic diagram analyzing cleavage results from (A). The differing line thickness represents the differing extent of cleavage activity of 2A^pro^ on each substrate. (**C, D, E**) Time-course study of 2A^pro^ on WT-MAVS (C), m-MAVS-251, (D) and m-MAVS-265 (E) stably expressed in their corresponding cell lines by western blot, which was carried out with 200 ng/µL recombinant 2A^pro^ at 30°C for indicated time.

Considering that MAVS translated *in vitro* may be slightly different from MAVS expressed in mammalian cells, such as in its protein conformation, stable cell lines were established to express wild-type MAVS (WT-MAVS) and MAVS mutants. Among the MAVS mutants, each residue was mutated individually and designated as m-MAVS-209, m-MAVS-251, and m-MAVS-265, and all three residues were also simultaneously mutated within one mutant (m-MAVS-3M). Cell lysates from the above cell lines were incubated with 2A^pro^ and then subject to western blot analysis to evaluate MAVS cleavage. Wild-type MAVS could be cleaved by 2A^pro^, resulting in two major cleavage fragments: CF209, a ∼40 kD peptide cleaved from Gly209, and CF251/265, a ∼34 kD product. CF251/265 might contain a mixture of cleavage fragments from CF251 and CF265 cleaved from Gly251 and Gly265, respectively. Gly251 and Gly265 lie close to each other within the protein, which might account for why these two cleavage fragments could not be distinguished from each other in the gel. While m-MAVS-3M is resistant to cleavage by 2A^pro^, m-MAVS-209, m-MAVS-251, and m-MAVS-265 exhibited different degrees of cleavage after incubation with 2A^pro^ (Figure 9A). 2A^pro^ showed the strongest cleavage ability against Gly251, followed by Gly209 and Gly265. This comes from the evidence that CF209 was more abundant than CF265 in 2A^pro^-treated cell lysates of m-MAVS-251 but was relatively less than CF251 in 2A^pro^-treated cell lysates of m-MAVS-265 (Figure 9A, lanes 8&10). These results were also consistent with the luminescence density detection results in the previous Protease-Glo screening assay, which showed that the vector encoding residues 243–254 induced the highest fold increase of luminescence density (68-fold), compared to the vector encoding residues 243–254 (40-fold) and 255–266 (13-fold) (Table 2). Figure 9B schematically summarizes the cleavage fragments and the degrees of cleavage that we could conclude from the above analysis.

To evaluate the cleavage order of each residue by the 2A^pro^ protease, we performed a kinetic analysis of 2A^pro^ on WT-MAVS, m-MAVS-251, and m-MAVS-265. Although all cleavage fragments exhibited a time-dependent increase upon incubation with 2A^pro^, the time they emerged slightly differed among them. CF251 emerged at 5 min, while CF209 and CF265 began to appear at 15 min (Figure 9C–E). Moreover, this assay verified that CF251 had the strongest band intensity, followed by CF209 and CF265 (Figure 9C–E), consistent with the results from Figure 9A. Taken together, these results suggest that 2A^pro^ exerts varying proteolysis ability on the different cleavage residues contained in MAVS and that Gly251 is the dominant residue that 2A^pro^ most strongly and rapidly cleaves.

### EV71-induced MAVS cleavage inhibits type I IFN production

**Figure 10:**
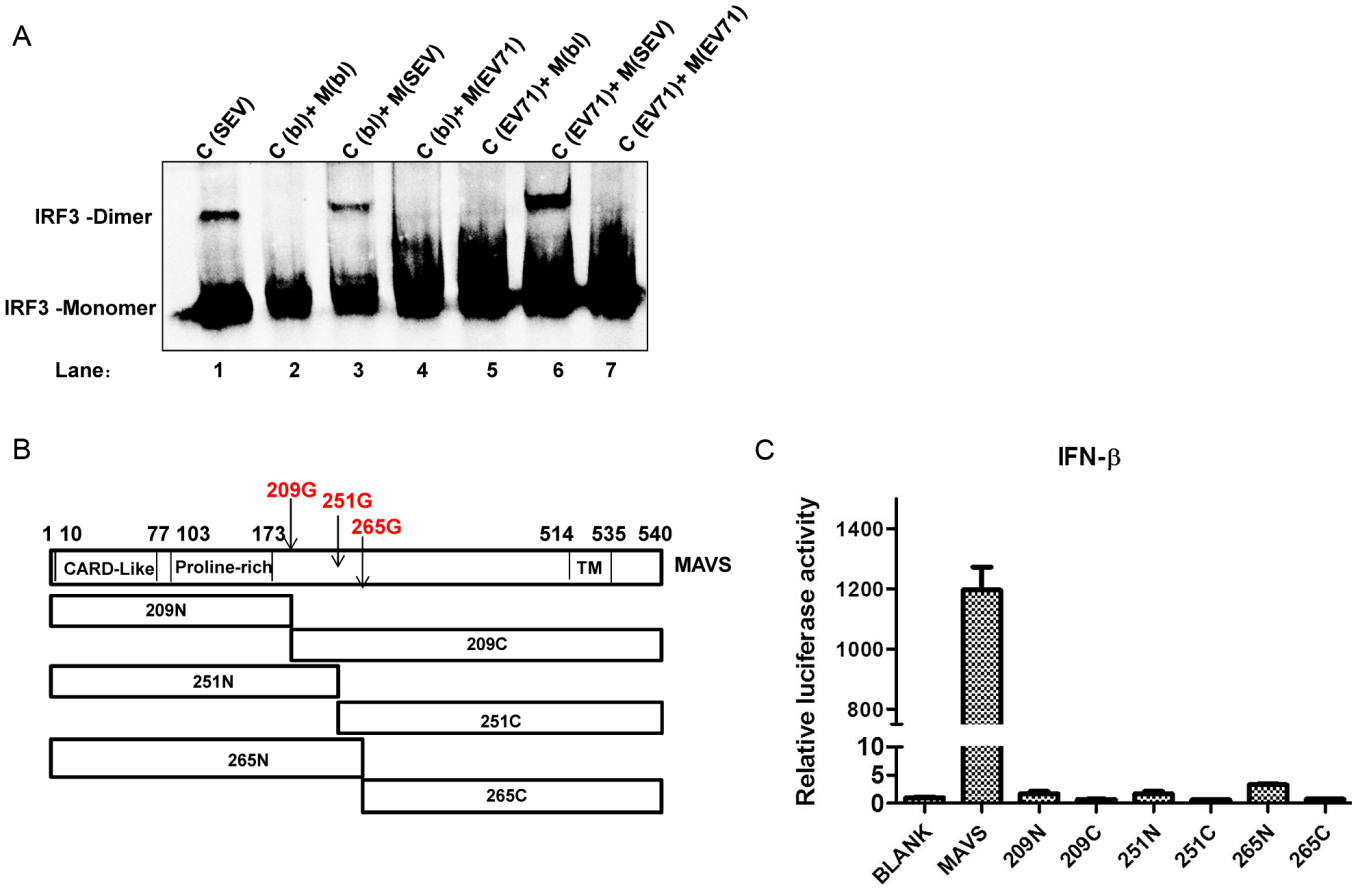
EV71-induced MAVS cleavage inhibits type I interferon production. (**A**) *In vitro* IRF3 dimerization assay using separated mitochondrial fractions from Blank (bl), SEV-infected, and EV71-infected cells incubated with the cytosolic compartments from Blank (lanes 2–4) and EV71-infected (lanes 5–7) HeLa cells. Cytosolic compartments from SEV-infected cells served as a positive control for IRF3 dimerization (lane 1) (M: mitochondrial; C: cytosol). The cells were infected for 18 h by both SEV and EV71 before the mitochondrial isolation procedure was performed. (**B**) Schematic diagram showing functional domains of MAVS, location of 2A^pro^ cleavage sites on MAVS, and constructed deletion mutants of MAVS. (**C**) Luciferase reporter assay of HeLa cells co-transfected with empty vector (Blank), MAVS, and MAVS deletion mutants from cleavage sites along with IFN-β promoter luciferase reporter plasmids and pRL-Actin control plasmid. Results are presented as relative luciferase activity and are expressed as the mean ± SD from two samples.

Since both MAVS and mitochondria are EV71 targets, we wondered whether normal mitochondria containing full-length MAVS could rescue the EV71-mediated inhibition of IRF3 activation. Zeng et al. had established a cell-free system demonstrating that mitochondria derived from SEV-infected cells could activate IRF3 in cytosol [57], [58]. Taking advantage of this system, we separated the mitochondrial and cytosolic compartments from mock-, SEV-, and EV71-infected cells, and reconstituted the RIG-I signaling pathway by exchanging the different compartments. While the mitochondria from SEV-infected cells dimerized IRF3 in the presence of mock-infected cytosol (Figure 10A, lane 3), mitochondria from EV71-infected cells inhibited this process (Figure 10A, lane 4). Moreover, mitochondria from SEV-infected cells rescued IRF3 activation in EV71-infected cytosol (Figure 10A, lane 6). These results suggest that MAVS cleavage and the associated mitochondrial changes might be a direct cause of EV71-induced inhibition of the innate immune response.

MAVS function requires mitochondrial localization. Since the EV71-induced MAVS cleavage occurred at three different residues between the proline-rich domain and the transmembrane domain, the N-terminal MAVS cleavage fragments would be released from the mitochondria. To test whether these cleavage fragments lost function in inducing type I IFN production, a series of deletion mutants from each cleavage residue was generated (Figure 10B) and transfected into HeLa cells with an IFN-β luciferase reporter plasmid. While full-length MAVS strongly activated the IFN-β promoter (nearly 1200-fold), none of the deletion mutants could activate the promoter, suggesting that the EV71-induced MAVS cleavage inactivated the signaling cascade leading to type I IFN production (Figure 10C).

## Discussion

EV71 is a member of the *Enterovirus* genus, *Picornaviridae* family. Its pathogenicity is likely related to its ability to evade host innate immunity. Although both the TLR3 and RIG-I/MDA-5 pathways recognize viral PAMPs and induce host anti-viral signaling during the innate immune response induced upon EV71 infection [1], [2], [59], the type I IFN response usually resulting from these pathways is totally absent [11]. The mechanism behind this observation is not clearly understood, although circumventing strategies have been found in RIG-I and TLR3 pathways [10], [11]. In this report, we reveal that another signaling molecule, MAVS, is cleaved by the EV71 viral protein 2A^pro^ at multiple residues that results in inhibiting type I IFN production. This novel finding can help to explain the influence of EV71 on both RIG-I and MDA-5 signaling transduction pathways and is a good supplement to the current understanding of how EV71 escapes host innate immunity.

The central role of MAVS in innate immunity predisposes it to being a target of many viruses. In recent years, several different viruses were reported to use various strategies to disrupt MAVS function. HCV-derived NS3/4A protease was the first viral protein reported to co-localize with MAVS at mitochondrial membranes and cleave MAVS at Cys508 [6], [13], [14], [16], and HBV-derived HBx protein was reported to bind MAVS and promote its degradation to inhibit IFN-β production [31], [60]. More interestingly, viruses within the *Picornaviridae* family cleave MAVS through various mechanisms and at different sites. HAV, a picornavirus belonging to the *Hepatovirus* genus, cleaves MAVS at Gln428 by the protease precursor 3ABC [18]. Rhinovirus cleaves MAVS by its 2A^pro^ and 3C^pro^ proteases as well as by activated caspase 3. Coxsackievirus B3 (CVB3), another member of *Enterovirus* genus in the *Picornaviridae* family, cleaves MAVS at Gln148 by its 3C^pro^ [19]. Our finding that EV71 2A^pro^ cleaved MAVS at Gly209, Gly251, and Gly265 provides a new insight into how virus-derived proteins and MAVS can interact. To our knowledge, our study is also the first to show that MAVS cleavage occurred at multiple residues to inhibit type I IFN production. All three cleavage residues reside within the region between the proline-rich domain and transmembrane domain of MAVS, and this region is relatively disorganized from a structural point of view and forms a reasonable docking structure for the approaching of 2A^pro^ protease. Mukherjee et al. previously studied MAVS expression in CVB3- and EV71-infected cells. While they found that CVB3 cleaved MAVS into fragments between 40–50 kD, they failed to detect these cleavage products in EV71-infected cells even though MAVS expression was significantly reduced in both cases; they speculated that MAVS was cleaved at other sites during EV71 infection [19]. Our studies confirmed their speculation, as EV71-induced MAVS cleavage not only occurred at other residues but also by a new mechanism. This finding provides new information regarding pathogen diversity as well as host-pathogen antagonism.

Due in part to the identification that mitochondrial-localized MAVS participates in the innate immune response, the idea that mitochondria not only play an important role in energy metabolism and cellular apoptosis but also provide a platform for virus-host interaction is now a generally accepted concept [3]–[6]. Consistent with this, some viral proteins also localize to the mitochondria to cleave MAVS as a way to circumvent innate immunity, like NS3/4A of HCV or the 3ABC precursor of HAV [6], [13], [14], [16], [18]. Also, mitochondrial dynamics and membrane potential have recently been recognized as essential for MAVS-mediated anti-viral signaling [36], [37]. These examples highlight the function of mitochondria as a platform structure in innate immunity, where viruses rely on its membrane structure and constitution to complete replication, and host cells utilize its membrane communication mechanisms to sense viral PAMPs and induce anti-viral immunity. In our study, we detected EV71 VP1 protein on mitochondria, raising the possibility that mitochondria may function at some particular stage of EV71 propagation; our results also further support the idea that EV71 could use this localization to cleave MAVS and destroy mitochondria to evade host innate immunity and provide another example for host-pathogen antagonism occurring on this intracellular-membrane platform. This finding could also explain the previously reported interaction between EV71 3C^pro^ and RIG-I [11]. RIG-I is recruited to a region nearby the mitochondria upon activation and interacts with MAVS via its CARD domain; the known role of 3C^pro^ in this process suggests that its presence is proximal to the mitochondria. In the literature, mitochondria have only been identified as a replication site for alphanodavirus flock house virus (FHV) [61], although the mitochondrial localization of HAV-derived 3ABC suggested an association with mitochondria in picornavirus replication [18]. Our current findings that EV71 VP1 co-localizes with mitochondria and that mitochondrial abnormalities were observed in EV71-infected cells strengthen the concept that mitochondria play a role in picornavirus replication. Future studies focusing on the specific mechanisms of mitochondria during the picornavirus life cycle should be carried out to further explore this concept.

EV71-encoded 2A^pro^ and 3C^pro^ proteases are responsible for processing poly-protein precursors to produce mature structural and non-structural viral proteins. Picornavirus proteases affect numerous host mechanisms. EV71 3C^pro^ had been identified as a strong antagonist of innate immunity, as it was shown to interact with RIG-I and cleave TRIF to inhibit the RIG-I– and TLR3-mediated anti-viral signaling [10], [11]. Picornavirus 2A^pro^, on the other hand, has been shown to hijack host-cell gene expression by cleaving eIF4GI, eIF4GII, and PABP, among other things [10], [11]. This gene “shutoff” mechanism also inhibits expression of IFN-stimulated genes and can therefore be considered another mechanism by which picornavirus regulates host innate immunity. Moreover, Enterovirus 2A^pro^ was also previously shown to be essential for its own replication in type I interferon-treated cells [62], and a recent study showed that EV71 2A^pro^ reduces IFN receptor I (IFNAR1) to inhibit type I IFN signaling, indicating that EV71 2A^pro^ functions as an antagonist to anti-viral innate immunity. Our finding that EV71 2A^pro^ strongly cleaves MAVS supports role for this protease in antagonizing innate immunity. Since our study as well as others showed that both EV71-encoded proteases target anti-viral innate immunity at multiple steps, it is possible they may act synergistically to ensure the effective immune-evasion of EV71. Our study also attempted to evaluate the contribution of the different mechanisms used by EV71 2A^pro^ and 3C^pro^ to antagonize innate immunity. We generated two mutated EV71 infectious clones, M-EV71-2A110 and M-EV71-3C40, that contained mutations at residue 110 of 2A^pro^ and at residue 40 of 3C^pro^, respectively, as these sites had previously been demonstrated to be indispensable for innate-immune inhibition by 2A^pro^ and 3C^pro^ in the above-mentioned study and in our previous study [11]. Unfortunately, we were not able to obtain EV71 mutants with these mutated proteases, as the mutations impeded EV71 production due to the critical nature of these residues in catalytic enzyme activity and in EV71 replication (Supplemental Figure S6).

In this study, we provided direct biochemical evidence that EV71 2A^pro^ protease cleaved MAVS using a cell-free *in vitro* system. This *in vitro* cleavage system is widely used and considered to be the most straight-forward approach to study the hydrolysis function of picornavirus proteases [42]–[48]. However, this system presented the following drawbacks as compared to the *in vivo* system: factors affecting the cleavage process in live cells might be omitted in the *in vitro* system, such as subcellular location; the *in vitro* cleavage-reaction buffer is different from the microenvironment in live cells and might cause slight conformational changes of the target proteins; and variation in the amount of recombinant protease, cleavage time, and temperature might induce non-specific cleavage that might confound the results. We speculate that these factors might help to explain the appearance of another MAVS cleavage band (Figure 7A–B, indicated by *) in our *in vitro* cleavage system that did not appear in EV71-infected cells, which we now think may represent a non-specific product.

When mapping protease cleavage site(s) on a target molecule, the routine method is to construct a series of mutants based on cleavage band size and bioinformatic analysis according to the hydrolyzing characteristics of the protease, followed by co-transfection of the mutants and protease into cells to test the predicted outcome. This approach requires accurate prediction, and missing potential cleavage sites is a possibility, especially when multiple cleavage sites exist. This routine strategy was not appropriate to use in our study for the following additional reasons. First, we cannot successfully express EV71 2A^pro^ at the required levels for verifying the speculated cleavage sites in regular cells, since 2A^pro^ was reported to hijack host-cell gene expression and also affect its own exogenous expression in mammalian cells. Second, we failed to observe the cleavage bands in cells over-expressing MAVS upon EV71 infection, which we originally thought was due to the poor viral replication inhibited by innate-immune activation. Therefore, two strategies were adopted to circumvent these issues, including: (i) establishing HeLa cell lines that stably express MAVS and MAVS mutants in which no sustained IRF3 activation was observed; and (ii) using P2.1 cells to transiently over-express MAVS for EV71 infection experiments. The P2.1 cell line is derived from the HT1080 cell line; it cannot respond to type I and type II IFNs because it lacks functional Jak1 and expresses very low IRF3 levels [63]. Despite these strategies, we still failed to observe cleavage bands from exogenously transfected MAVS (data not shown). Although the underlying reason is not yet clear, we speculate the following possibilities to explain these results: (i) the conformation and distribution of exogenously transfected MAVS might be different from endogenous MAVS; and (ii) exogenously transfected MAVS might have the potential to activate innate immunity and therefore induce and recruit MAVS-associated negative regulators that might prevent its interaction with downstream molecules. This latter possibility was hinted at by a report showing that PCBP2 is a negative regulator of MAVS-mediated signaling [29], and association of MAVS with other proteins might also prevent any effect of EV71. We therefore switched strategies and took advantage of the Protease-Glo assay system to screen the whole MAVS extra-membrane region. Using these methods, we successfully identified three MAVS residues cleaved by the EV71 2A^pro^ and confirmed this in both the *in vitro* translated MAVS-EM and the stably expressed MAVS in HeLa cells.

When using exogenous MAVS and MAVS mutants expressed in HeLa cells to evaluate MAVS cleavage, the cleavage fragments recognized by the HA antibody are located in the C-terminus of MAVS and its mutants; they are indeed the corresponding counterparts to the endogenous N-terminal cleavage fragments recognized by the anti-MAVS antibodies used in EV71-infected cells (Figure 2A–B). This can be deduced from the molecular weight size and band intensity of the cleavage fragments. Full-length endogenous MAVS is approximately 65 kD in size, and the two cleavage fragments resulting from EV71 infection are both approximately 30 kD, where one appears above the 30 kD molecular weight band (∼31 kD) and the other one appears below the 30 kD band (∼25 kD). These bands seem to be counterparts to and coincident with the observed 34 kD (CF251/265) and 40 kD (CF209) bands in Figure 9C–E, including their respective band intensities.

Overall, we showed in this study that the EV71-derived 2A^pro^ cleaves the key adaptor molecule MAVS as a strategy to evade anti-viral innate immunity at the signal transduction phase. Furthermore, we identified three key residues cleaved by the 2A^pro^ protease activity on the extracellular fragment of MAVS. Our findings therefore reveal a new mechanism of EV71 viral protease-mediated evasion of host innate immunity.

## Materials and Methods

### Cells and viruses

Rhabdomyosarcoma (RD) cells and HeLa cells were purchased from ATCC. RD cells were cultured in MEM supplemented with 10% FBS and penicillin/streptomycin. HeLa cells were cultured in DMEM supplemented with 10% FBS and penicillin/streptomycin. 2FTGH-ISRE cells were a gift from Dr. Zhengfan Jiang (School of Life Sciences, Peking University, China). BSRT7/5 cells were cultured in DMEM supplemented with 10% FBS and 1 mg/mL G418. Enterovirus 71 (EV71) is a Fuyang strain isolated from a child in the city of Fuyang with a clinical diagnosis of HFMD in 2008 (GenBank accession no. FJ439769.1), and was propagated in RD cells. Sendai virus (SEV) was kindly provided by Dr. Zhengfan Jiang and propagated in chicken embryos.

### Plasmids

The PGL3-IFNβ-Luc, pNifty-Luc, and pRL-Actin plasmids were gifts from Dr. Zhengfan Jiang. Mito-dsRed was provided by Dr. Xuejun Jiang (Institute of Microbiology, Chinese Academy of Sciences, China). pEGFPC1-EV71-3ABC was constructed by inserting EV71 3ABC cDNA fragment into the Hind III and Sal I sites of the pEGFPC1 vector. The plasmid expressing EV71 2A^pro^ was generated by PCR amplification from PEGFPC1-EV71-2A as described before [11] and cloned into pET 30a (+) vector. Plasmid expressing EV71 2A^pro^-110 was mutated by PCR using pET 30a (+)-2A as template. The MAVS construct and its mutants were generated by PCR amplification from GFP-MAVS (provided by Dr. Zhengfan Jiang) and cloned into the pcDNA3.1 (+) vector. pcDNA3.1-IRES-2A was a gift from Dr. Shih-Yen Lo (Department of Laboratory Medicine and Biotechnology, Tzu Chi University, Hualien, Taiwan) and described before [53].

### Antibodies and reagents

Mouse monoclonal antibodies directed against β-Actin (AC-15) and GFP (GSN24) were purchased from Sigma. Rabbit polyclonal antibody against HA was purchased from Bethyl Laboratories. Rabbit polyclonal antibodies against IRF-3 (FL-425) and cytochrome c (7H8) were purchased from Santa Cruz Biotechnology. Mouse anti-MAVS (E-3, monoclonal antibody raised against residues 1–135 of human MAVS) and rabbit anti-MAVS (AT107, polyclonal antibody raised against residues 160–450 of human MAVS) were obtained from Santa Cruz Biotechnology and Enzo Life Sciences, respectively. Another MAVS antibody, which reacts with human, mouse, and rabbit MAVS, was purchased from Signalway Antibody and used in western blot analysis of BSRT7/5 cells. Mouse anti-KDEL (10C3, recognizes GPR78 and GPR94 with particular prominence), mouse anti-mitochondria (MTC02, recognizes a 60 kD non-glycosylated protein component of human mitochondria), rabbit anti-caspase 3, and mouse anti-PABP (10E10) were obtained from Abcam. Rabbit anti-PARP, rabbit anti-caspase 8 (D35G2), and rabbit anti-caspase 9 were obtained from Cell Signaling Technologies. Mouse anti-enterovirus 71 was purchased from Millipore. Mouse anti-enterovirus 71 VP1 (3D7) was purchased from Abnova. Rabbit anti-Sendai antibody was purchased from MBL International Corporation. The general caspase inhibitor benzyloxycarbonyl-Val-Ala-Asp-(OMe) fluoromethylketone (Z-VAD-FMK) and proteasome inhibitor MG132 were purchased from Sigma and Calbiochem, respectively.

### Reporter assay and type I IFN bioassay

HeLa cells (∼2×10^5^) were seeded on 24-well dishes and transfected the following day by Lipofectamine 2000 (Invitrogen) with 200 ng of PGL3-IFNβ-Luc or pNifty-Luc and 5 ng pRL-Actin. Cells were co-transfected with 600 ng of the indicated plasmids or infected with EV71/SEV 24 h post-transfection. In all experiments, cells were lysed and reporter activity was analyzed using the Dual-Luciferase Reporter Assay System (Promega). The type I IFN bioassay was performed as previously reported by Sun et al. [64]. Briefly, the supernatant from SEV- and EV71-infected cells were collected at the indicated times, added directly to 96-well dishes seeded with 2FTGH-ISRE cells, and luciferase activity was measured after 6 h and calculated with reference to a recombinant human IFN-β standard (R&D system).

### Native PAGE

Native PAGE was carried out as previously described [65]. Native gel (8%) was pre-run with native running buffer (25 mM Tris and 192 mM glycine, pH 8.4) with 0.5% deoxycholate in the cathode chamber for 30 min at 25 mA on ice. Samples were prepared in the native sample buffer (62.5 mM Tris–HCl, pH 6.8, 15% glycerol, and 1% deoxycholate), then loaded onto the gel and electrophoresed at 20 mA for an additional 1 h.

### Western blot

Whole-cell extracts (20–100 µg) were separated by 8%–15% SDS-PAGE. After electrophoresis, proteins were transferred to a PVDF membrane (Bio-Rad). The membranes were blocked for 1 h at room temperature in 5% dried milk and then were probed with the indicated primary antibodies at an appropriate dilution overnight at 4°C. The following day, the membranes were incubated with corresponding IRD Flour 680- or 800-labeled IgG secondary antibodies (LI-COR Biosciences) and were scanned by the Odyssey Infrared Imaging System (LI-COR Biosciences).

### Immunofluorescence

Cells were fixed in 4% formaldehyde, permeabilized in 0.5% Triton X-100, blocked in 1% BSA in PBS, and then probed with indicated primary antibodies for 1 h at room temperature. Following a wash, cells were incubated with their respective secondary antibodies for another 1 h. The cells were then washed and stained with 4, 6-diamidino-2-phenylindole (DAPI) to detect nuclei. Images were captured with a laser confocal microscope (Leica).

### Mitochondrial isolation and purification

Mitochondrial isolation was carried out by differential centrifugation. Briefly, cells were harvested and resuspended in HB buffer (210 mM mannitol, 70 mM sucrose, 5 mM HEPES, pH 7.12, 1 mM EGTA, and an EDTA-free protease inhibitor cocktail) and subject to homogenization. After 30 strokes, cell homogenate was centrifuged at 600×*g* for 10 min at 4°C. The supernatant was saved and subjected to further centrifugation at 10000×*g* for 10 min at 4°C. The pellet was washed once with HB buffer and designated as the crude mitochondrial fraction. The supernatant was further centrifuged at 12000×*g* and designated as the cytosol fraction after discarding the final pellet. Mitochondria purification was performed by Percoll gradient fractionation as previously described with minor modifications [41], [66], [67]. A schematic overview of the isolation and purification protocol is displayed in Figure 6B.

### Recombinant protein expression and purification, and *in vitro* cleavage assay

Recombinant EV71 3C^pro^ was produced as described before [68]. To produce EV71 2A^pro^ and 2A^pro^-110, the respective plasmids were introduced into competent *E. coli* BL21 (DE3) cells, and protein expression was induced by treatment with 200 µM IPTG at 18°C overnight. 2A-His fusion protein was purified by Ni-Agarose column. *In vitro* cleavage assay was performed with the indicated amount of recombinant protease incubated together with cell lysates in reaction buffer (50 mM Tris-HCl, pH 7.0, and 200 mM NaCl) at 37°C for 6 h or 30°C for 2 h.

### Flow cytometric analysis

Mitochondrial membrane potential was analyzed using Flow Cytometry Mitochondrial Membrane Potential Detection Kit (BD Biosciences) by a BD FACS Canto II flow cytometer (BD Biosciences). The experiments were carried out according to the manufacturer's instructions.

### Protease-Glo assay and *in vitro* transcription/translation of the MAVS extra-membrane region

Synthesized oligonucleotides encoding 12-mer peptides (with six amino-acid overlap between two adjacent 12-mers) for the MAVS extra-membrane region were inserted in pGloSensor-10F linear vector (Promega). The resulting vectors were subjected to *in vitro* transcription/translation with TNT SP6 High-Yield Wheat Germ Protein Expression System (Promega) and FluoroTect Green_Lys_ *in vitro* Translation Labeling System (Promega) according to manufacturer's instructions. The reactions were incubated at 25°C for 2 h. Then, 7 µg of recombinant EV71 2A^pro^ or 3C^pro^ was added to 10 µL reactions with 10 µL 2× digestion buffer (100 mM Tris-HCl, pH 7.0, and 400 mM NaCl). The digestion reactions were incubated for 2 h at 30°C, and a 10 µL aliquot was removed and subjected to 10% SDS-PAGE. The gels were scanned by a Typhoon gel scanner (GE Healthcare) to visualize the fluorescently labeled proteins. The remaining 10 µL was diluted 20-fold, and luciferase activity was measured using the Bright-Glo assay reagent (Promega) according to the manufacturer's instructions. *In vitro* transcription/translation of the MAVS extra-membrane region was performed by the TNT SP6 High-Yield Wheat Germ Protein Expression System Labeled with FluoroTect Green_Lys_. The DNA template for this assay was constructed by amplifying the MAVS coding region at residues 1–513 and cloned into the pF3AWG (BYDV) Flexi Vector (Promega).

### Generation of cell lines stably expressing MAVS and MAVS mutants

HeLa cells were transfected with pcDNA3.1-MAVS and its mutants by Lipofectamine 2000 (Invitrogen) and selected in Zeocin (200 µg/mL) to establish the cell lines stably expressing MAVS and MAVS mutants.

### *In Vitro* IRF3 activation assay

*In Vitro* IRF3 activation assay was carried out as previously described by Zeng et al [57], [58]. Briefly, HeLa cells were resuspended in Buffer A (10 mM Tris-HCl pH 7.5, 10 mM KCl, 0.5 mM EGTA, 1.5 mM MgCl_2_, 0.25 M D-mannitol, and EDTA-protease inhibitor cocktail) and homogenated. Then, the homogenates were centrifuged at 1000×*g* at 4°C for 5 min; the supernatants were further centrifuged at 5000×*g* at 4°C for 10 min to separate the pellets (P5) and the supernatants (S5). P5 was washed once with Buffer B (20 mM HEPES-KOH pH 7.4, 0.5 mM EGTA, 0.25 M D-mannitol, and EDTA-protease inhibitor cocktail) and resuspended in Buffer B. For each reaction, 10 µg P5 and 20 µg S5 were mixed in Buffer C (20 mM HEPES-KOH pH 7.0, 2 mM ATP, 5 mM MgCl_2_) and incubated at 30°C for 1 h in a 10 µL reaction system. The reaction mixtures were then subjected to native PAGE, and the dimerization of endogenous IRF3 was detected by western blot.

### Gene IDs

MAVS (HGNC: 29233); RIG-I (HGNC: 19102); MDA-5 (HGNC: 18873); TLR3 (HGNC: 11849); IRF3 (HGNC: 6118); TRIF (HGNC: 18348); Caspase 3 (HGNC: 1504); Caspase 8 (HGNC: 1509); Caspase 9 (HGNC: 1511); PARP (HGNC: 270); PABP (HGNC: 8554); RANTES (HGNC: 10632); IFN-β (HGNC: 5434); PCBP2 (HGNC: 8648); RNF125 (HGNC: 21150); RNF5 (HGNC: 10068); IFNAR1 (HGNC: 5432); Cytochrome c (HGNC: 19986).

## Supporting Information

Figure S1 **EV71 inhibits type I interferon responses upstream of IRF3 activation.** **(A)** HeLa cells were infected with EV71 (MOI = 10) and SEV (20 HA/mL) for the indicated time. Total RNA extracted from cells was used for RT-PCR to detect mRNA of IFN-β, RANTES, and GAPDH. **(B)** For the luciferase assay, HeLa cells were co-transfected with IFN-β and NF-κB promoter luciferase reporter plasmids with pRL-Actin control plasmid. At 24 h post transfection, the cells were infected with EV71 (MOI = 10) and SEV (20 HA/mL) for the indicated times. Results are presented as relative luciferase activity and are expressed as mean ± SD among three samples.
(TIF)

Figure S2 **Anti-EV71 antibody reacted against EV71 structural protein VP2.** 293T cells were transfected with plasmids encoding various EV71 viral proteins fused with GFP. The parental vector pEGFPC1 (lane 11) and EV71-infected cells (MOI = 10, lane 12) were included as controls. At 24 h post transfection/infection, cells were fixed, and in-cell western blot analysis was carried out with an anti-EV71 antibody.
(TIF)

Figure S3 **Various 2A^pro^ substrates reacted differently to 2A^pro^ proteolytic activity.** Western blot analysis for MAVS, PABP and eIF4GI in **(A)** HeLa cells transfected with increasing doses (0–4 µg) of pcDNA3.1-IRES-2A plasmid, and **(B)** HeLa cells were infected with EV71 (MOI = 10) for the indicated time.
(TIF)

Figure S4 **Protease-Glo assay of 3C^pro^ activity on MAVS.** Data depicts the screening assay testing 3C^pro^ activity on 86 constructs containing the coding region for the 12-mer polypeptides covering the MAVS extra-membrane region. Luciferase assay results are shown together with gel analysis results.
(TIF)

Figure S5 **EV71 3C^pro^ could not cleave MAVS translated** ***in vitro*** **.** The extra-membrane region of MAVS (MAVS-EM) was translated by the TNT SP6 High-Yield Wheat Germ Protein Expression System Labeled with FluoroTect Green_Lys_. The reaction mixture was incubated with recombinant EV71 3C^pro^ (lane 2) and 2A^pro^ (lane 3), then resolved by gel analysis and visualized by the Typhoon gel scanner (GE Healthcare).
(TIF)

Figure S6 **2A^pro^ and 3C^pro^ mutations in EV71 infectious clones inhibit virus production.** Immunofluorescence detecting the presence of EV71 virus in HeLa cells infected with supernatant from Vero cells transfected with RNA transcripts derived from a wild-type EV71 infectious clone (upper panel), a 2A^pro^ mutated infectious clone (M-EV71-2A110) (middle panel), and a 3C^pro^ mutated infectious clone (M-EV71-3C40) (lower panel).
(TIF)

Text S1 **Supporting Materials and Methods.**
(DOCX)
