# Correction: Enterovirus 71 Protease 2A^pro^ Targets MAVS to Inhibit Anti-Viral Type I Interferon Responses

**DOI:** 10.1371/journal.ppat.1006243

**Published:** 2017-03-02

**Authors:** Bei Wang, Xueyan Xi, Xiaobo Lei, Xiaoyan Zhang, Sheng Cui, Jianwei Wang, Qi Jin, Zhendong Zhao

The authors would like to correct Fig 5A, as an error was introduced in the preparation of this figure for publication. In Fig 5A, the “mock” graph was duplicated and also appears as the “4h” graph. The authors have replaced the duplicated graph with the correct “4h” graph.

**Fig 5:**
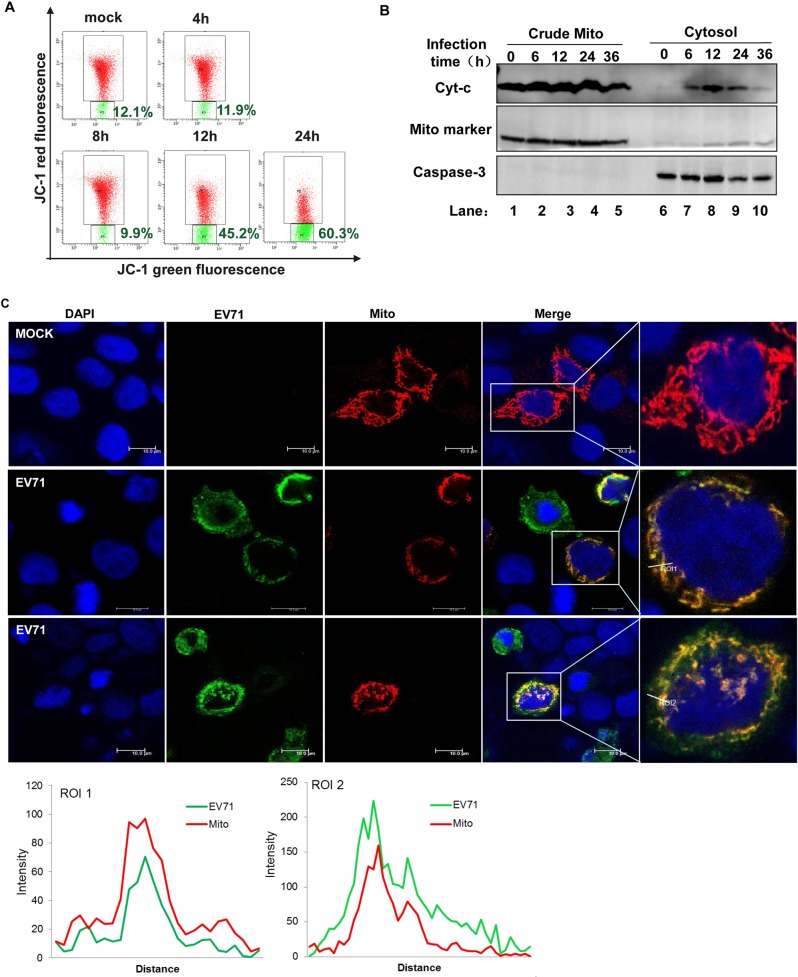
EV71 targets mitochondria to inhibit innate immunity. HeLa cells were infected with EV71 (MOI = 10) for the indicated time. (A) Mitochondrial membrane potential was analyzed by flow cytometry method using 5,5′,6,6′-tetrachloro-1,1′,3,3′-tetramethyl benzimidazolyl carbocyanine iodide (JC-1). The vertical and horizontal coordinates represented JC-1 red and green fluorescence, respectively. (B) Cells were subjected to differential centrifugation to separate the crude mitochondrial (lanes 1–5) from the cytosolic (lanes 6–10) compartment. Western blot analysis was performed on these two compartments to detect cytochrome c (cyt-c) (upper panel). Mito marker (middle panel) and caspase 3 (lower panel) served as the mitochondrial and cytosolic markers, respectively [64]. (C) HeLa cells were transfected with Mito-dsRed plasmid. At 24 h post-transfection, cells were infected with EV71 (MOI = 10, middle and lower panel). At 24 h post-infection, cells were fixed and stained for EV71 using an antibody against EV71. Morphological changes in the mitochondria were analyzed under confocal microscopy (nucleus: blue; EV71: green; mitochondria: red; Scale bar: 10 μm). Histograms show the fluorescence intensity analysis results from ROI1 and ROI2 in the merged panel. All images represent 3 independent experiments.

Additionally, when the authors were alerted to the errors, they re-ran the software for all graphs to re-create the duplicated graph, and in re-running the software with the raw data, the gating percentages for the “mock” and “12H” graphs changed slightly. The authors have provided a corrected Fig 5 with both the updated graphs and gating percentages.

The authors apologize for the errors and confirm that these changes do not alter their findings.
