# Correction: Enterovirus 71 Protease 2A^pro^ Targets MAVS to Inhibit Anti-Viral Type I Interferon Responses

**DOI:** 10.1371/journal.ppat.1012209

**Published:** 2024-05-06

**Authors:** Bei Wang, Xueyan Xi, Xiaobo Lei, Xiaoyan Zhang, Sheng Cui, Jianwei Wang, Qi Jin, Zhendong Zhao

After publication and correction of this article [1, 2], concerns were raised about Figs 2B, 7E and 9A and S4. Specifically:

1. In Fig 2B, when levels are adjusted to visualize the background, there appear to be vertical discontinuities between lanes 1 and 2 in both the E-3 and merge panels.
2. In Fig 7E, there appears to be a vertical discontinuity in the background of the right 2A/2A110 panel, between lanes 14 and 15.
3. In Fig 9A, in the top panel, there appear to be vertical discontinuities in the background between lanes 1 and 2; lanes 5 and 6; lanes 7 and 8; and lanes 9 and10.
4. In S4 Fig, when levels are adjusted to visualize the background, there appear to be vertical discontinuities in some panels and the images are not annotated to demark different gels.

The first author provided corrected figures and the following explanations:

The molecular weight markers from repeat experiments were used in the E-3 and merge panels of Fig 2B. The corrected figure includes the marker lane from the same original blot as the experimental results shown in the figure.

For Fig 7B, lanes 1–8 and lanes 9–12 were combined from two blot images. The corrected Fig 7B has vertical lines to clearly indicate different image sources. For Fig 7E, when samples in the 2A/2A110 right panel were examined in SDS-PAGE, samples in lanes 15 and 16 were loaded in the reverse order. Lanes 15 and 16 were thus rearranged during figure preparation. In the corrected Fig 7E a vertical line indicates where the image was spliced.

The top panel in Fig 9A was the combination of two representative western blot images, and except for the m-MAVS-251 lanes the PABP and Actin data shown in the lower panels of the figure are from a different experiment than the data shown in the upper panel (see S1 File). The corrected figure shows the result from a single image with a vertical line to indicate where lanes were removed.

The results in S4 Fig were the combination of screening data aimed at identifying the 3C^pro^ cleavage sites in MAVS. The large number of screening samples was examined in multiple gels. The corrected figure has vertical lines to clearly indicate different image sources.

The raw data underlying the corrected figures are provided in S2 File. Raw data underlying other results in the article are in S3 File.

The authors apologize for the errors in the published article.

*Note*: *Corresponding author Zhendong Zhao is deceased*. *Future inquiries about this article should be directed to the first author Bei Wang at* wangbei20170217@163.com.

**Fig 2:**
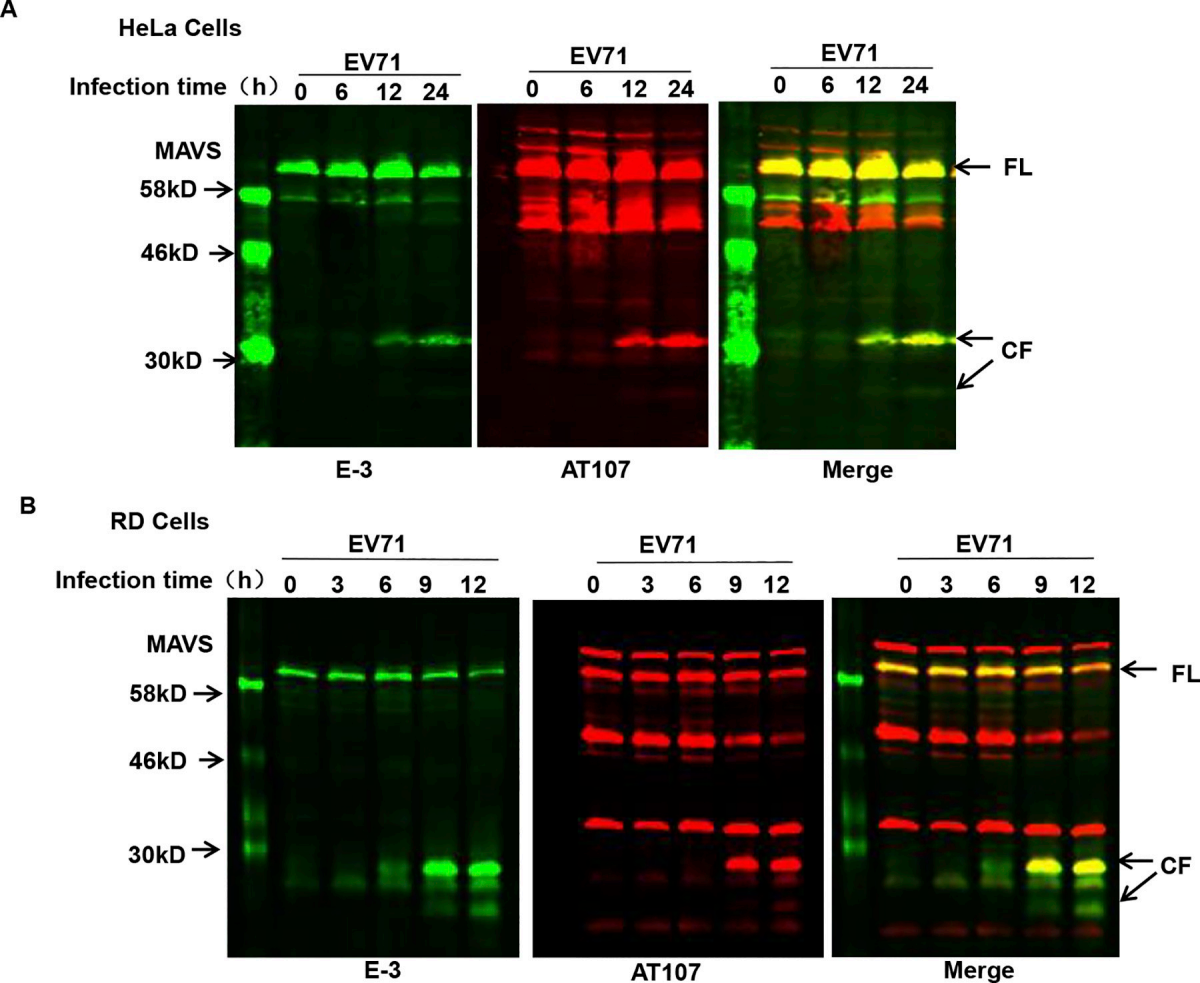
MAVS is cleaved in EV71-infected cells. Western blot analysis of MAVS expression in EV71-infected (MOI  =  10) (**A**) HeLa cells and (**B**) RD cells for the indicated time. The time course evaluating MAVS expression was carried out by LI-COR Odyssey Dual-Color System using two different antibodies against MAVS (E-3, 700 nm, green; AT107, 800 nm, red). Results are displayed as images from each channel as well as an overlaid image of the two channels. Arrows indicate full-length MAVS (FL) and the cleaved fragments (CF) derived from MAVS.

**Fig 7:**
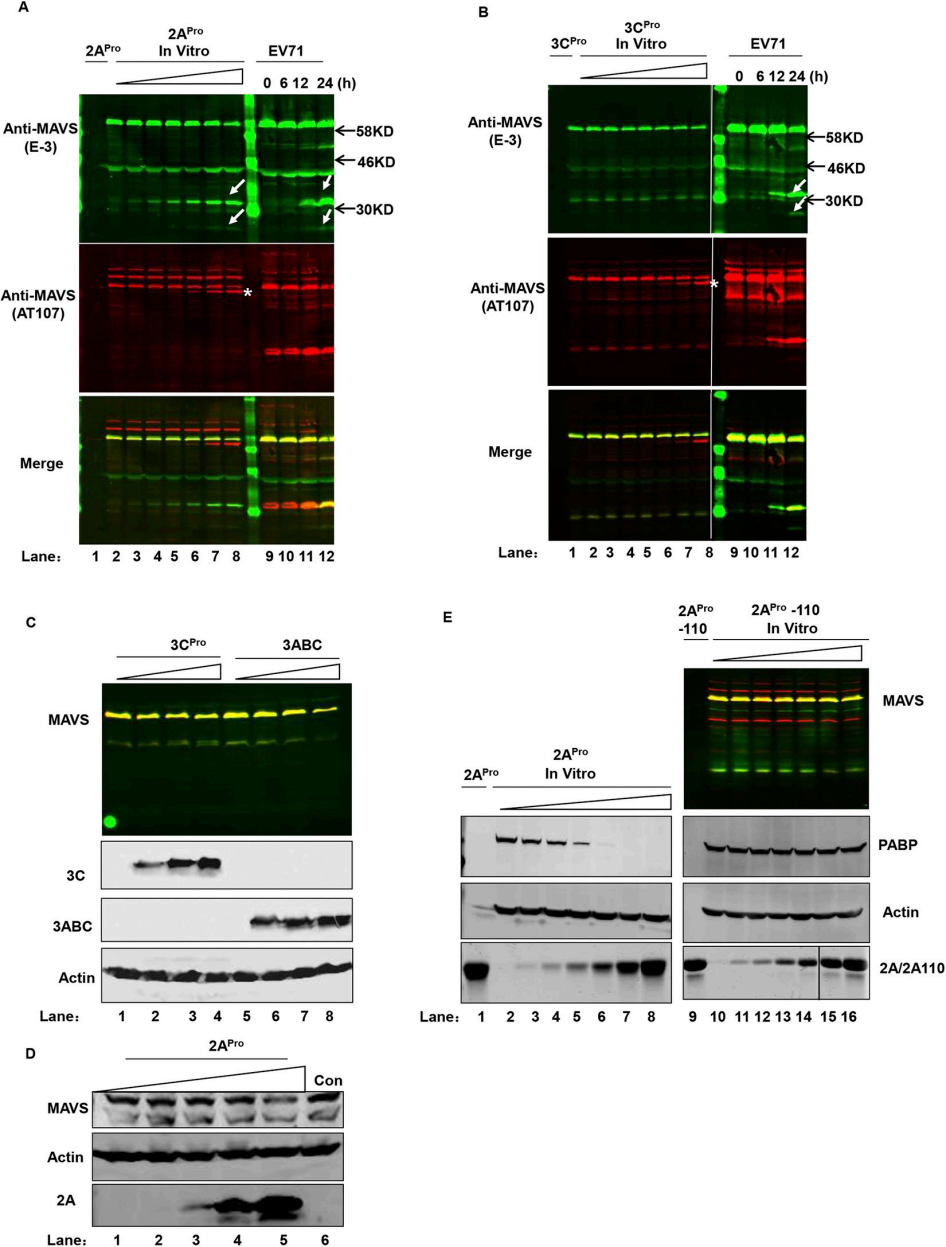
EV71 2A^pro^ cleaves MAVS. (**A, B**) *In vitro* dose- dependent cleavage assay of EV71 2A^pro^ (A) and 3C^pro^ (B) on MAVS using LI-COR Odyssey Dual-Color System. Increasing doses of recombinant proteases were added to the cell lysates and incubated at 37°C for 6 h (from 0–200 ng/µL, lanes 2–8); recombinant proteases (lane 1) and EV71-infected HeLa cells (lanes 9–12) served as negative and positive controls, respectively. Two antibodies recognizing different MAVS epitopes were used (E-3, 700 nm, green; AT107, 800 nm, red). An overlay of the two channels is shown in the “Merge” panel. White arrows indicate cleaved bands in EV71-infected HeLa cells and the same-size bands in 2A^pro^-cleaved HeLa extracts. (**C**) Western blot analysis of MAVS in HeLa cells transfected with increasing doses of plasmids (0–4 µg) encoding EV71 3C^pro^ (lanes 1–4) and 3ABC (lanes 5–8) precursor protein fused with GFP. The MAVS image is an overlay of two signals from the different channels described in (A). The same cell lysates were also used to detect 3C^pro^ and 3ABC using an antibody against GFP; actin served as the loading control. (**D**) Western blot analysis of MAVS in BSRT7/5 cells transfected with increasing doses of pcDNA3.1-IRES-2A plasmid (lanes 1–5, 0–4 µg) and pcDNA3.1-EGFP control plasmid (4 µg). (**E**) *In vitro* dosage cleavage assay (0–200 ng/µL) of 2A^pro^ (lanes 2–8) and mutated 2A^pro^ (2A^pro^-110) (lanes 10–16) on MAVS; recombinant 2A^pro^ (lane 1) and 2A^pro^-110 (lane 9) served as negative controls. The MAVS image is an overlay of two signals from the different channels described in (A). PABP was used as a readout for the enzyme activity of 2A^pro^ and 2A^pro^-110; actin served as a loading control. The image of 2A^pro^ and 2A^pro^-110 (lower panel) is a direct scan of SDS-PAGE gel stained with Coomassie brilliant blue.

**Fig 9:**
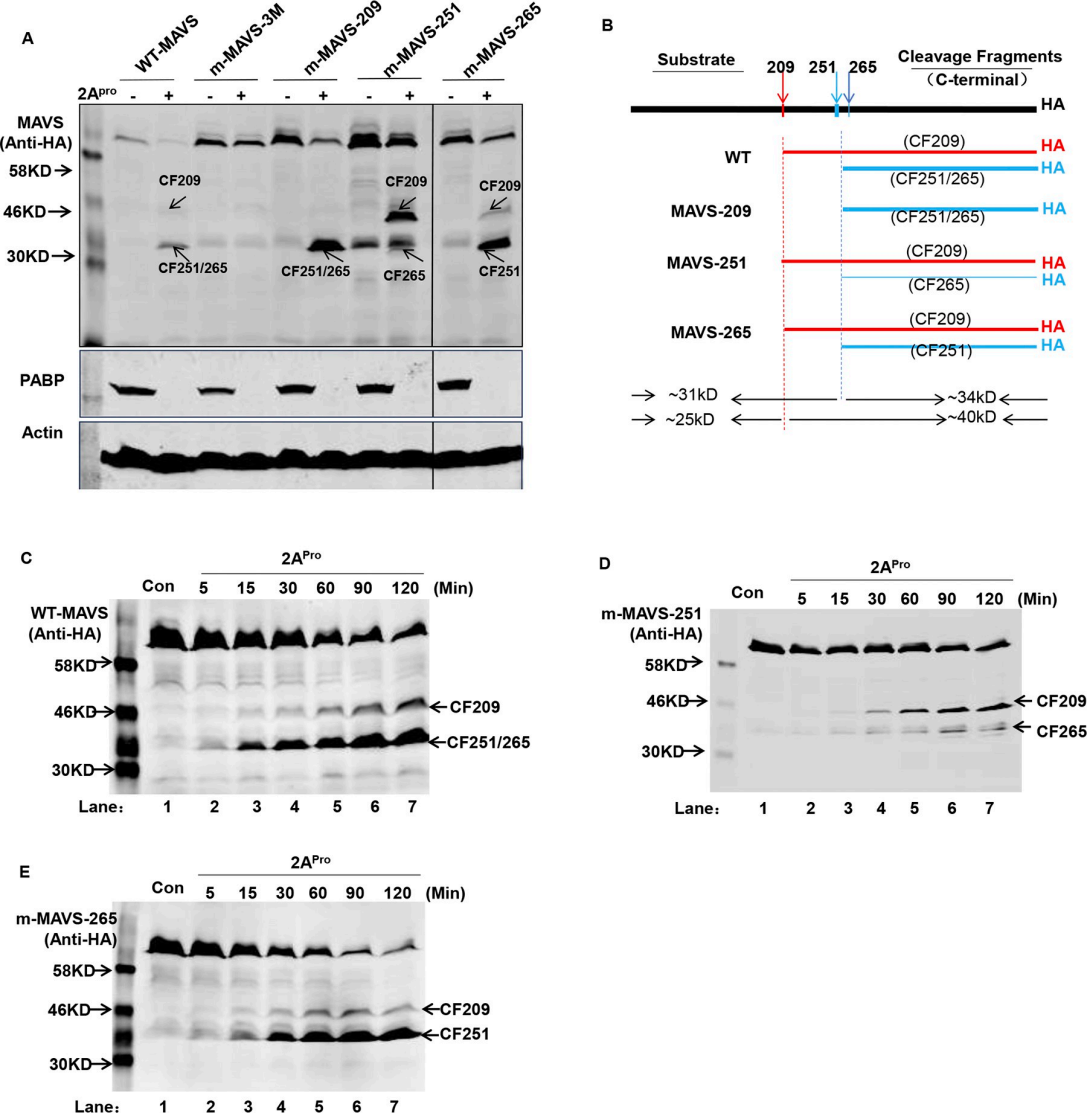
*In vitro* cleavage assay of EV71 2A^pro^ on MAVS and MAVS mutants expressed in HeLa cells. (**A**) The cell lysates from stable cell lines expressing WT-MAVS (lanes 1&2), m-MAVS-3M (lanes 3&4), m-MAVS-209 (lanes 5&6), m-MAVS-251 (lanes 7&8), and m-MAVS-265 (lanes 9&10) were incubated with 200 ng/µL 2A^pro^ at 30°C for 2 h; the cell lysates were then subjected to western blot analysis to probe MAVS using an HA antibody against an HA peptide fused to the C-terminus of MAVS and MAVS mutants. Cleavage of PABP served as a readout for the enzymatic activity of 2A^pro^. (**B**) Schematic diagram analyzing cleavage results from (A). The differing line thickness represents the differing extent of cleavage activity of 2A^pro^ on each substrate. (**C, D, E**) Time-course study of 2A^pro^ on WT-MAVS (C), m-MAVS-251, (D) and m-MAVS-265 (E) stably expressed in their corresponding cell lines by western blot, which was carried out with 200 ng/µL recombinant 2A^pro^ at 30°C for indicated time.

## Supporting information

S4 Fig Protease-Glo assay of 3C^pro^ activity on MAVS. Data depicts the screening assay testing 3C^pro^ activity on 86 constructs containing the coding region for the 12-mer polypeptides covering the MAVS extra-membrane region. Luciferase assay results are shown together with gel analysis results.
(TIF)

S1 File Raw image data underlying the originally published version of Fig 9A. (PDF)

S2 File Raw image data underlying corrected Figs 2, 7, 9 and S4. (ZIP)

S3 File Raw data underlying other figures. (ZIP)
