# Supplementary figures and images for: Enterovirus 71 Protease 2Apro Targets MAVS to Inhibit Anti-Viral Type I Interferon Responses
Source: PLoS Pathog. 2013 Mar 21;9(3):e1003231. doi: 10.1371/journal.ppat.1003231 (PMC3605153; doi:10.1371/journal.ppat.1003231)

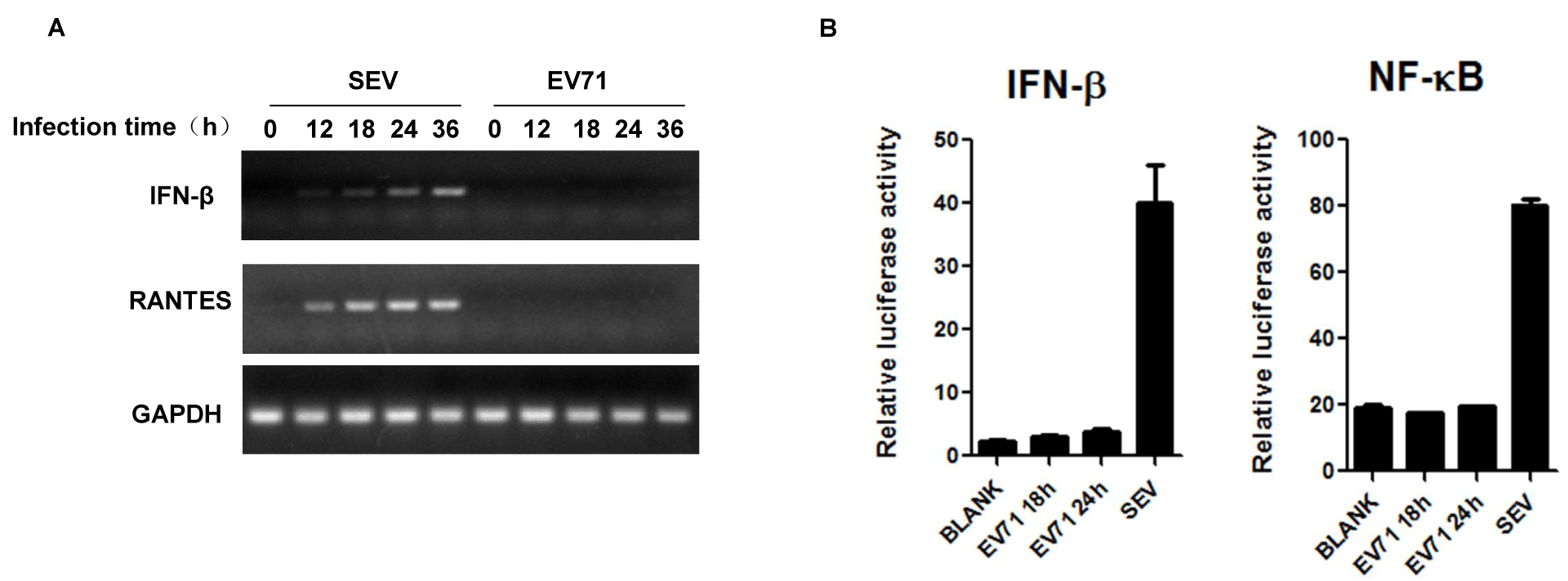

Supplement: Figure S1 — EV71 inhibits type I interferon responses upstream of IRF3 activation. (A) HeLa cells were infected with EV71 (MOI = 10) and SEV (20 HA/mL) for the indicated time. Total RNA extracted from cells was used for RT-PCR to detect mRNA of IFN-β, RANTES, and GAPDH. (B) For the luciferase assay, HeLa cells were co-transfected with IFN-β and NF-κB promoter luciferase reporter plasmids with pRL-Actin control plasmid. At 24 h post transfection, the cells were infected with EV71 (MOI = 10) and SEV (20 HA/mL) for the indicated times. Results are presented as relative luciferase activity and are expressed as mean ± SD among three samples. (TIF) [file ppat.1003231.s001.tif]

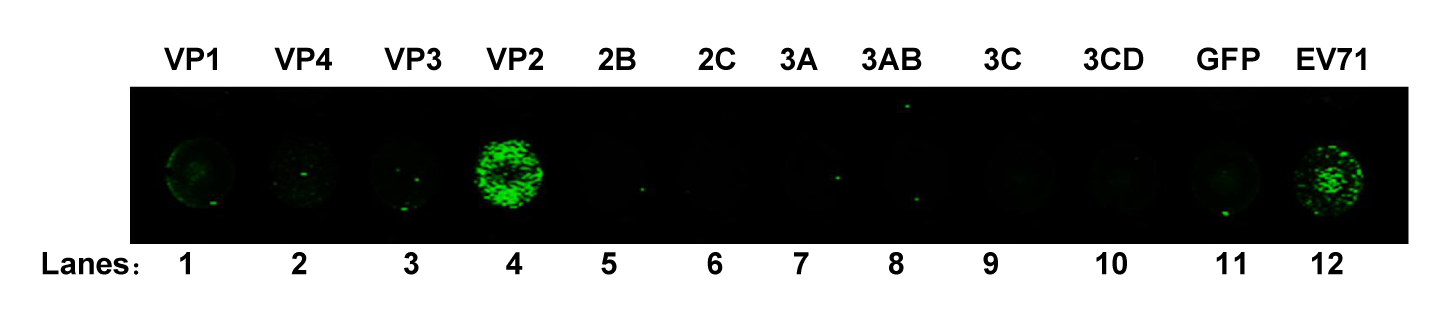

Supplement: Figure S2 — Anti-EV71 antibody reacted against EV71 structural protein VP2. 293T cells were transfected with plasmids encoding various EV71 viral proteins fused with GFP. The parental vector pEGFPC1 (lane 11) and EV71-infected cells (MOI = 10, lane 12) were included as controls. At 24 h post transfection/infection, cells were fixed, and in-cell western blot analysis was carried out with an anti-EV71 antibody. (TIF) [file ppat.1003231.s002.tif]

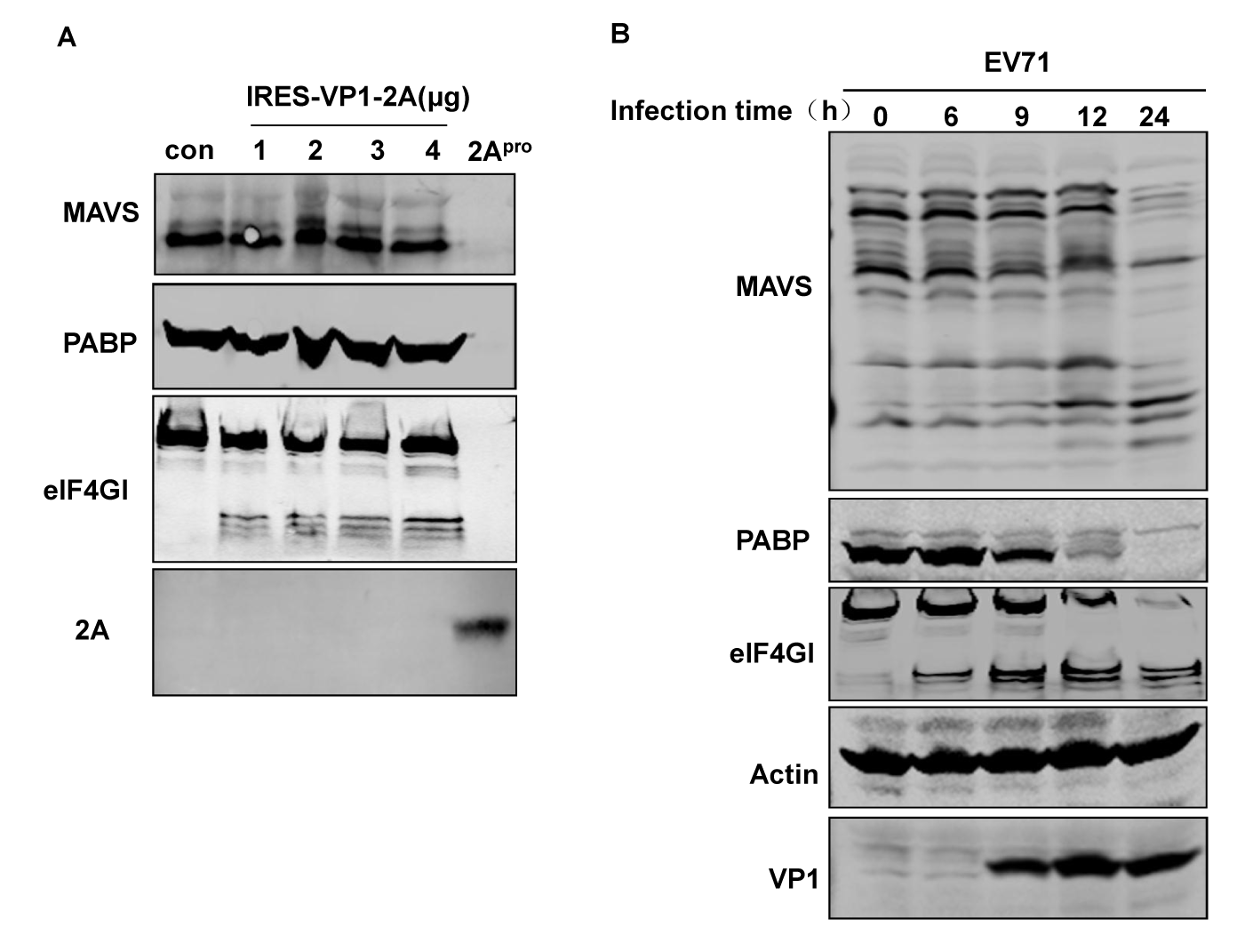

Supplement: Figure S3 — Various 2Apro substrates reacted differently to 2Apro proteolytic activity. Western blot analysis for MAVS, PABP and eIF4GI in (A) HeLa cells transfected with increasing doses (0–4 µg) of pcDNA3.1-IRES-2A plasmid, and (B) HeLa cells were infected with EV71 (MOI = 10) for the indicated time. (TIF) [file ppat.1003231.s003.tif]

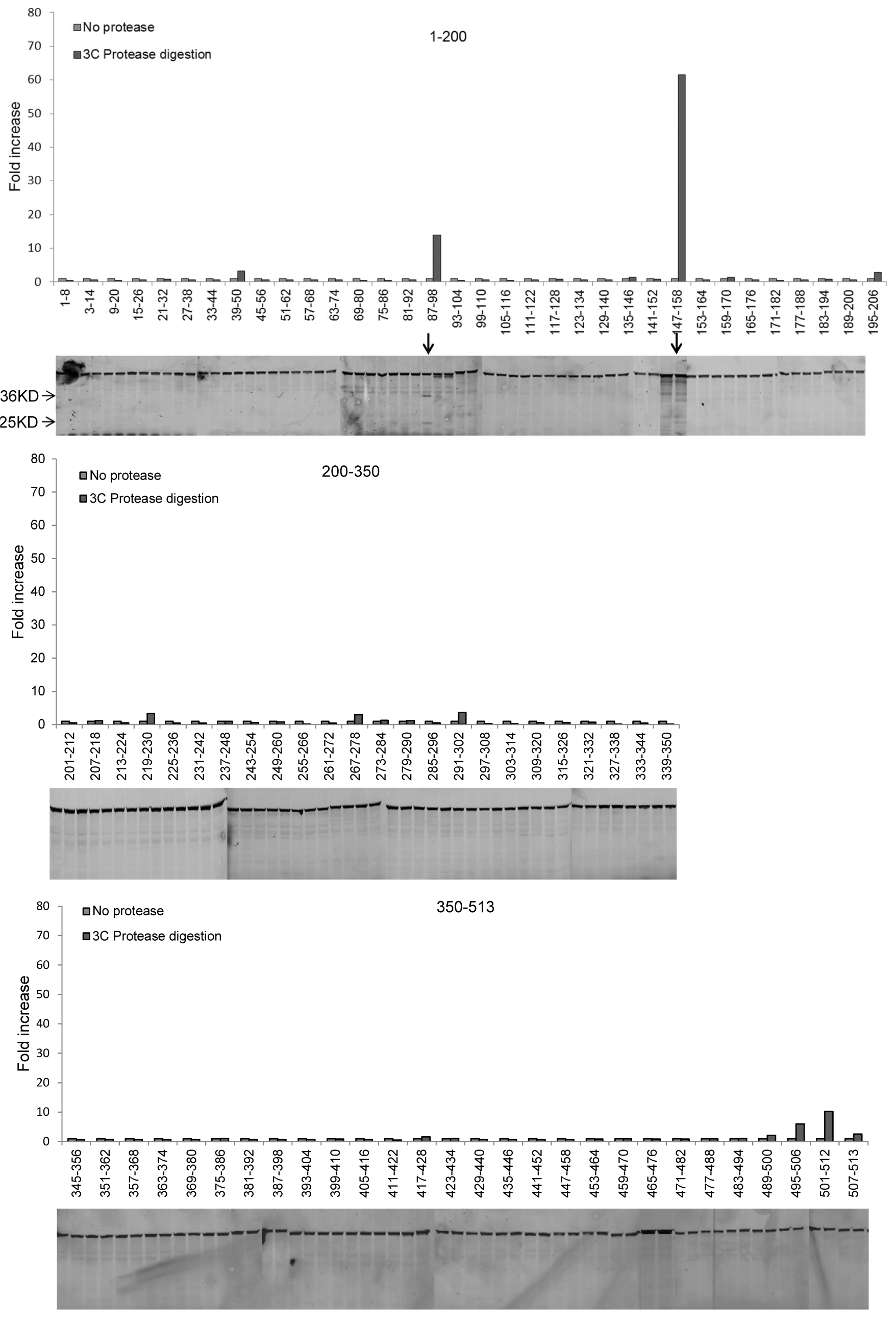

Supplement: Figure S4 — Protease-Glo assay of 3Cpro activity on MAVS. Data depicts the screening assay testing 3Cpro activity on 86 constructs containing the coding region for the 12-mer polypeptides covering the MAVS extra-membrane region. Luciferase assay results are shown together with gel analysis results. (TIF) [file ppat.1003231.s004.tif]

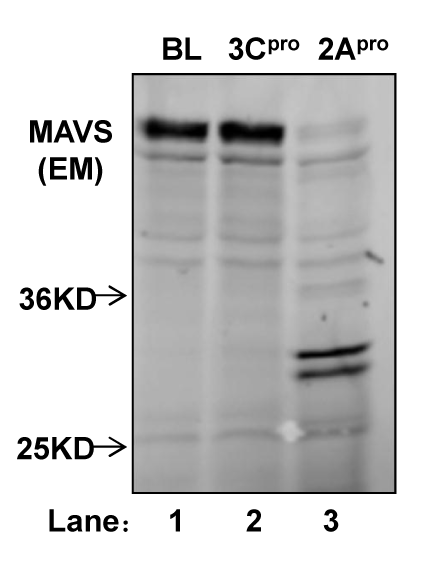

Supplement: Figure S5 — EV71 3Cpro could not cleave MAVS translated in vitro . The extra-membrane region of MAVS (MAVS-EM) was translated by the TNT SP6 High-Yield Wheat Germ Protein Expression System Labeled with FluoroTect GreenLys. The reaction mixture was incubated with recombinant EV71 3Cpro (lane 2) and 2Apro (lane 3), then resolved by gel analysis and visualized by the Typhoon gel scanner (GE Healthcare). (TIF) [file ppat.1003231.s005.tif]

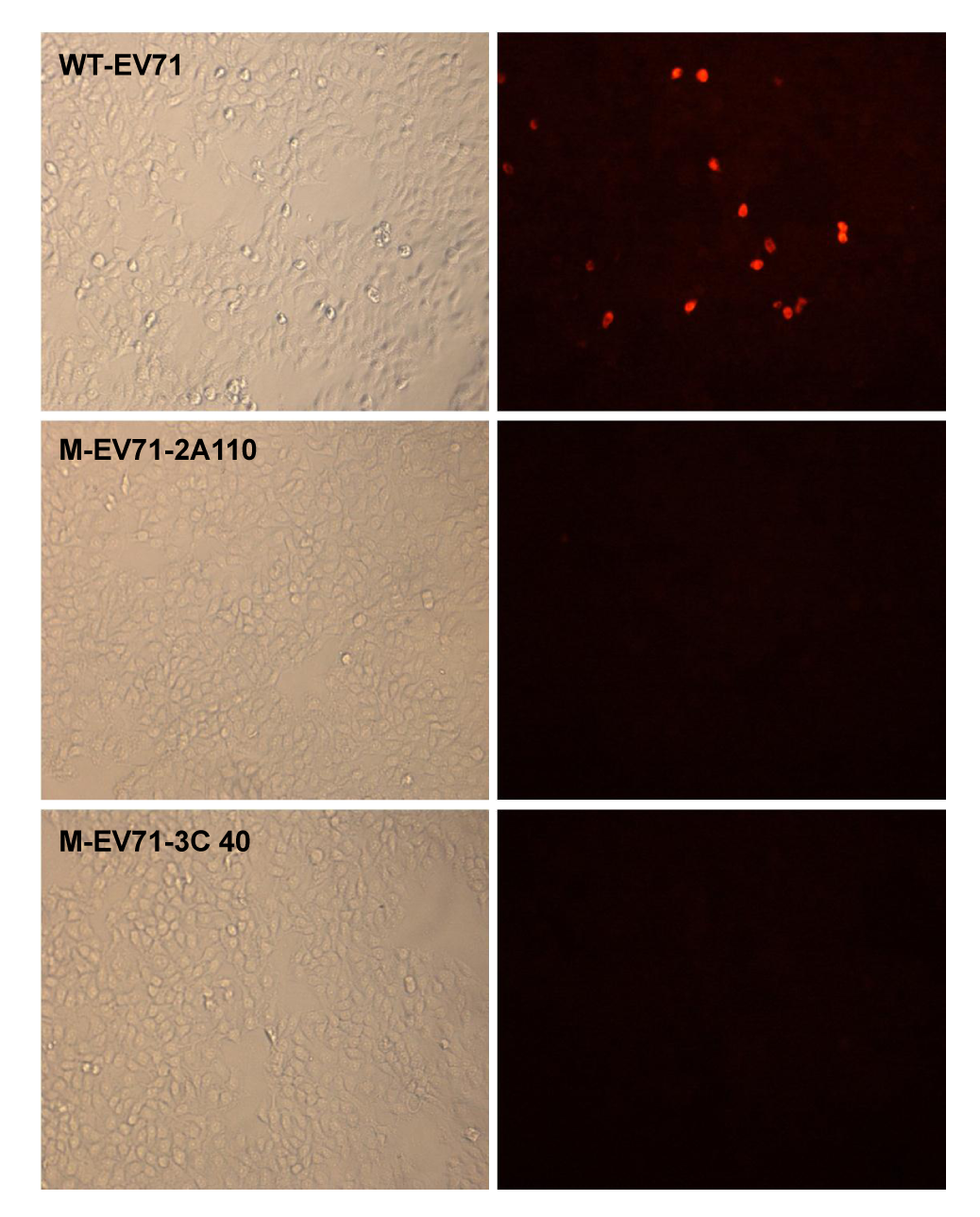

Supplement: Figure S6 — 2Apro and 3Cpro mutations in EV71 infectious clones inhibit virus production. Immunofluorescence detecting the presence of EV71 virus in HeLa cells infected with supernatant from Vero cells transfected with RNA transcripts derived from a wild-type EV71 infectious clone (upper panel), a 2Apro mutated infectious clone (M-EV71-2A110) (middle panel), and a 3Cpro mutated infectious clone (M-EV71-3C40) (lower panel). (TIF) [file ppat.1003231.s006.tif]
