# Supplementary material for: Enterovirus 71 Protease 2Apro Targets MAVS to Inhibit Anti-Viral Type I Interferon Responses
Source: PLoS Pathog. 2013 Mar 21;9(3):e1003231. doi: 10.1371/journal.ppat.1003231 (PMC3605153; doi:10.1371/journal.ppat.1003231)
Supplement: Text S1 — Supporting Materials and Methods. (DOCX) [file ppat.1003231.s007.docx]

**Supporting Materials and Methods**

**Cells**

Vero cells were purchased from ATCC and cultured in DMEM supplemented with 10% FBS and penicillin/streptomycin.

**Reverse transcription PCR**

HeLa cells were plated and infected by EV71 or SEV. At the indicated time points post-infection, total cellular RNA was extracted from cells using TRIzol (Invitrogen). RNA samples were quantified by spectrophotometry at 260 nm using Nanodrop (Nanodrop Technologies). RNA (1 μg) was used for reverse transcription using the Reverse Transcription System (Promega). cDNA samples were subject to PCR amplification for IFN-β, RANTES, and GAPDH. Primers sequences used were as follows:

IFN-β F: 5′-CCAACAAGTGTCTCCTCCAA-3′

IFN-β R: 5′-ATAGTCTCATTCCAGCCAGT-3′

RANTES F: 5′-CCCCGTGCCCACATCAAGGAGTATTT-3′

RANTES R: 5′-CGTCCAGCCTGGGGAAGGTTTTTGTA-3′

GAPDH F: 5′-AAAATCAAGTGGGGCGATGCT-3′

GAPDH R: 5′-GGGCAGAGATGATGACCCTTT-3′

**In-cell western blot analysis**

In-cell western blot assays were performed as described previously [11]. Briefly, 293T cells (~4 × 10^4^) were seeded on 96-well dishes and either transfected with plasmids encoding GFP fused to EV71 viral proteins [11] or infected with EV71 the next day. At 24 h post-transfection/infection, cells were fixed with 4% formaldehyde and permeabilized in 0.5% Triton X-100. After washing with PBS, cells were incubated with anti-EV71 antibody overnight at 4°C. After washing with PBS, cells were incubated with IRD Flour 680-labeled goat anti-mouse IgG. Then, cells were scanned by the LI-COR Odyssey Dual-Color System.

**EV71 virus production from EV71 and mutated-EV71 infectious clones**

The EV71 infectious clone was a gift from Dr. Shan Cen (Institute of Medicinal Biotechnology, Chinese Academy of Medical Sciences & Peking Union Medical College). The mutated infectious clones were generated by PCR amplification using the wild-type EV71 infectious clone as a template. RNA transcripts were synthesized *in vitro* from linearized templates using MEGAscript® T7 Kit (Invitrogen). Then, RNA transcripts were transfected into Vero cells. At 60 h post-transfection, supernatants were collected and used to infect HeLa cells. At 24 h post-infection, HeLa cells were fixed, and immunofluorescence was performed to detect EV71 production using an anti-EV71 antibody.
