# Supplementary figures and images for: Correction: Enterovirus 71 Protease 2Apro Targets MAVS to Inhibit Anti-Viral Type I Interferon Responses
Source: PLoS Pathog. 2024 May 6;20(5):e1012209. doi: 10.1371/journal.ppat.1012209 (PMC11073730; doi:10.1371/journal.ppat.1012209)

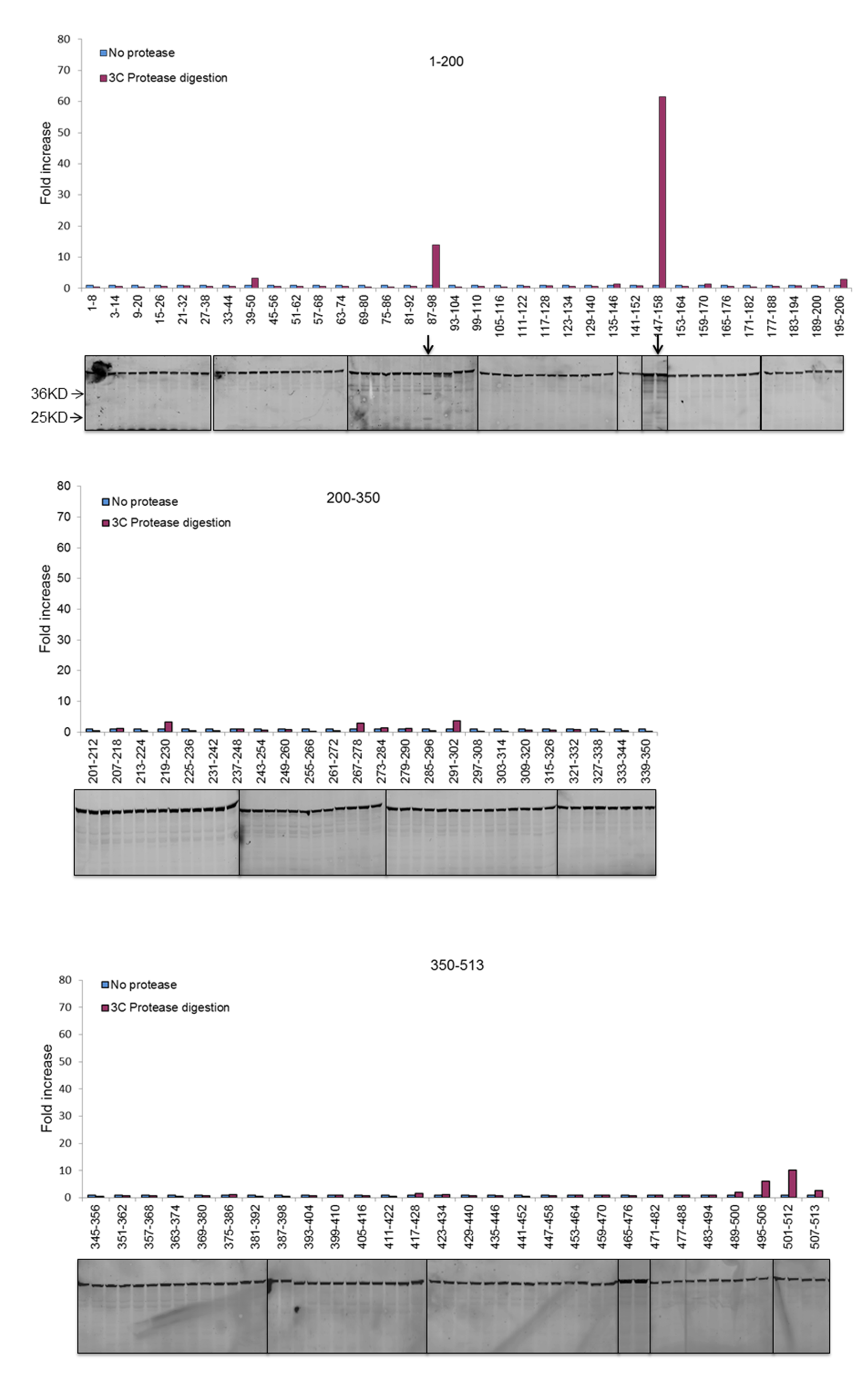

Supplement: S4 Fig — Data depicts the screening assay testing 3Cpro activity on 86 constructs containing the coding region for the 12-mer polypeptides covering the MAVS extra-membrane region. Luciferase assay results are shown together with gel analysis results. (TIF) [file ppat.1012209.s001.tif]

# All images for Figure 9A

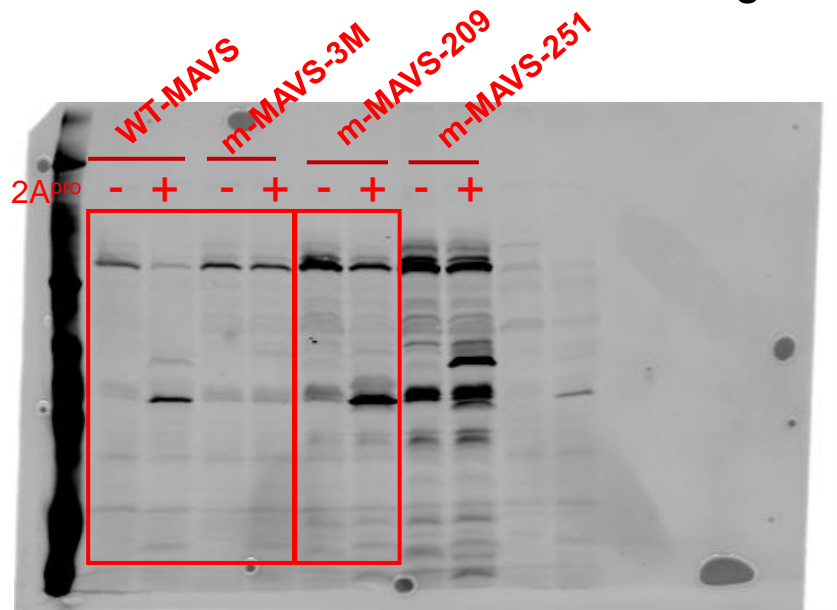

Image in the published paper

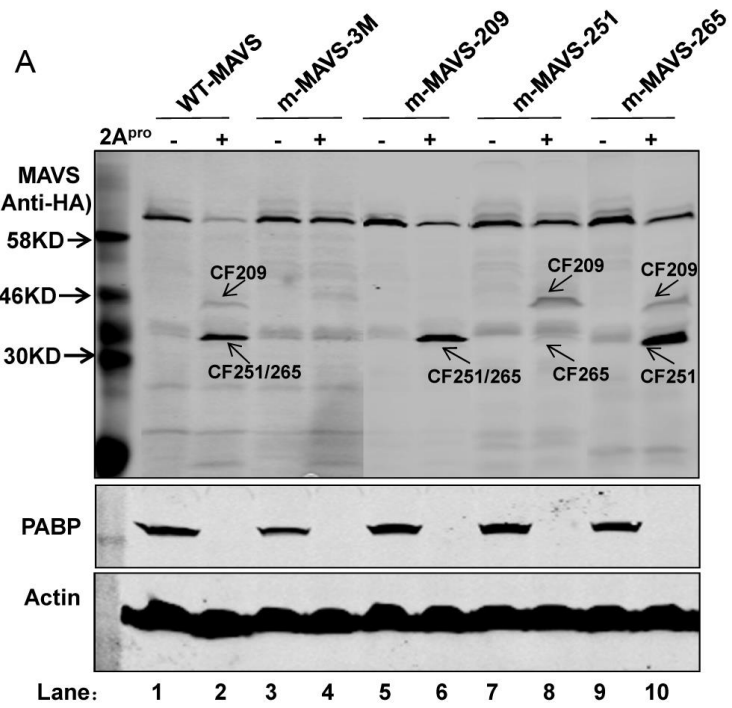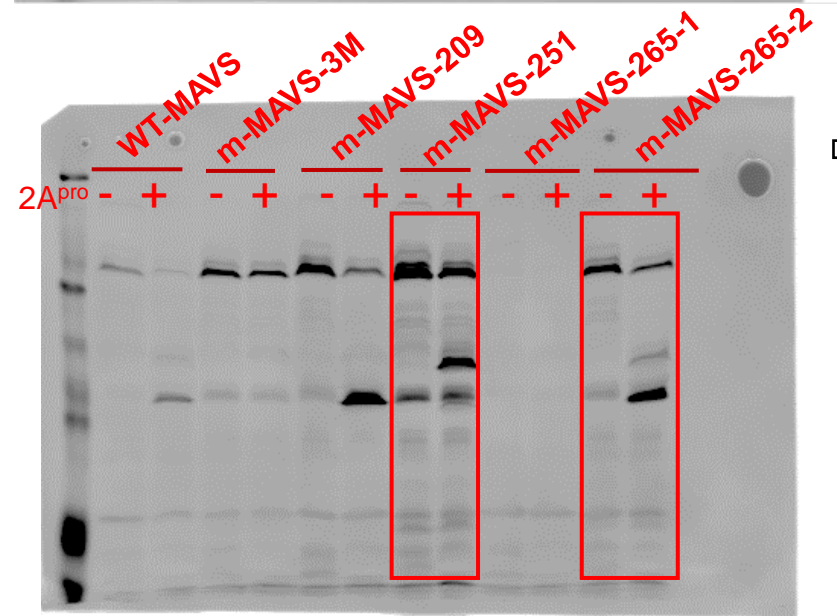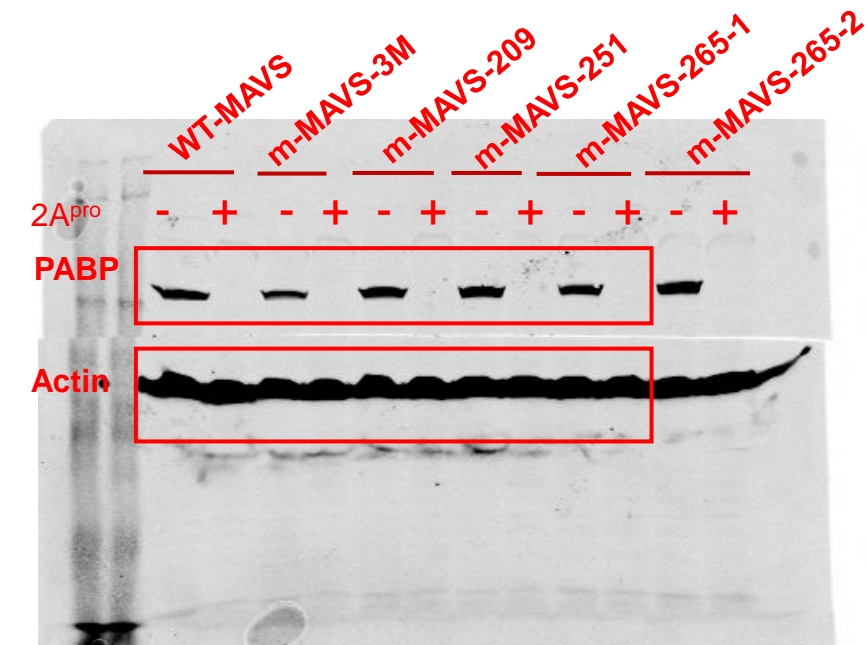

Supplement: S1 File — (PDF) [file ppat.1012209.s002.pdf]

# All images for Figure 7E

MAVS Lane 9-16

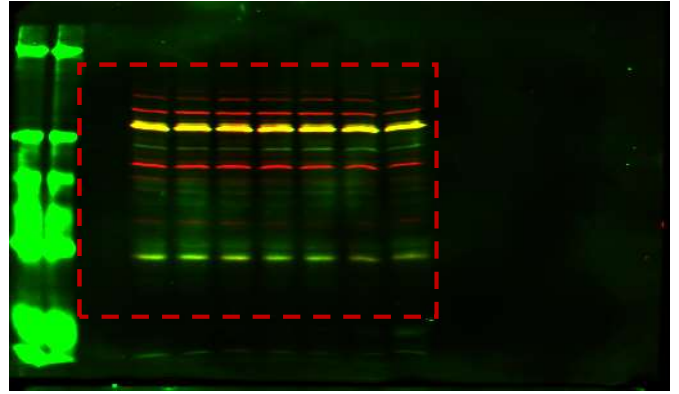

PABP Lane 1-8

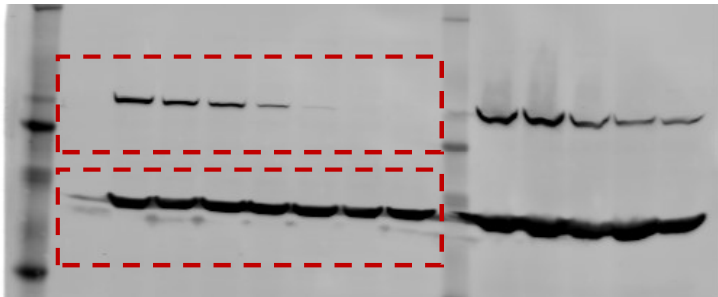

Actin Lane 1-8

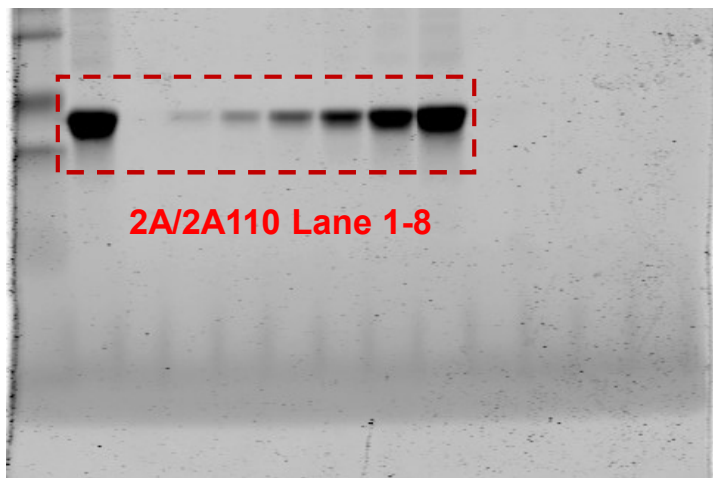

2A/2A110 Lane 1-8

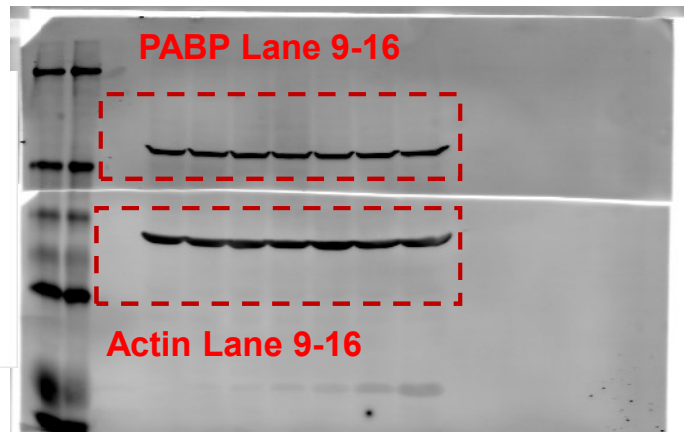

Actin Lane 9-16

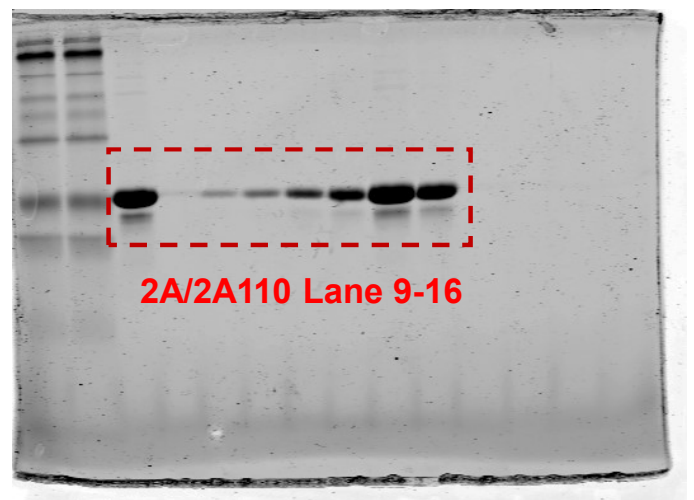

2A/2A110 Lane 9-16

Supplement: S2 File — (ZIP) [file ppat.1012209.s003.zip › S2 file/raw data-fig7E.pdf]

All images for Figure S4

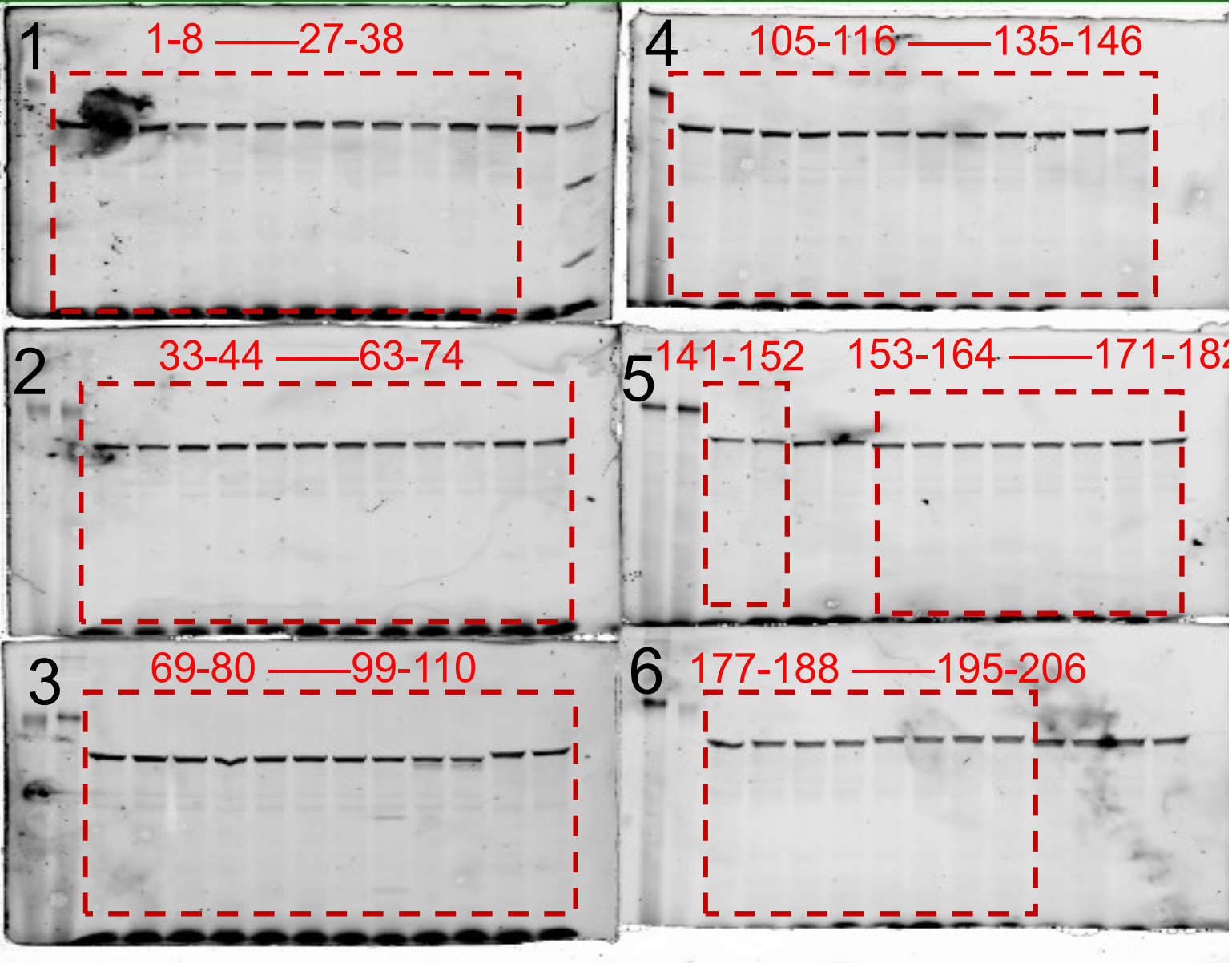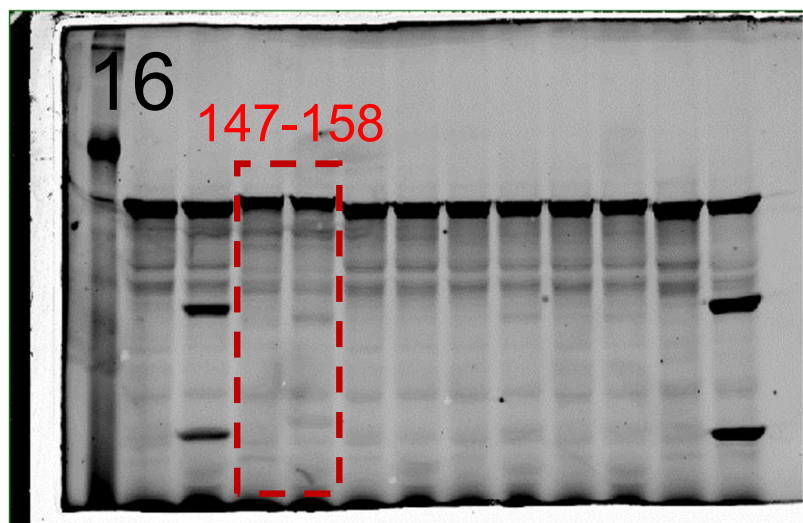

7 201-212 — 237-248

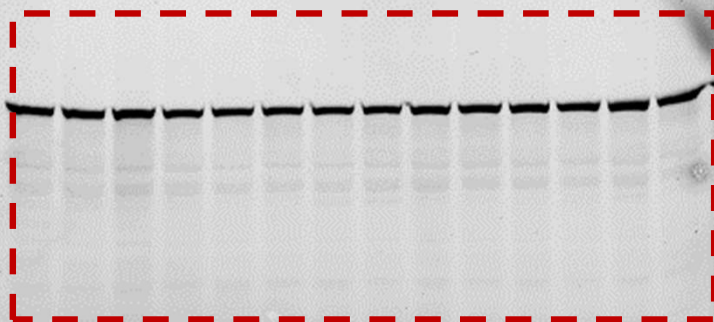

10 321-332 — 339-350

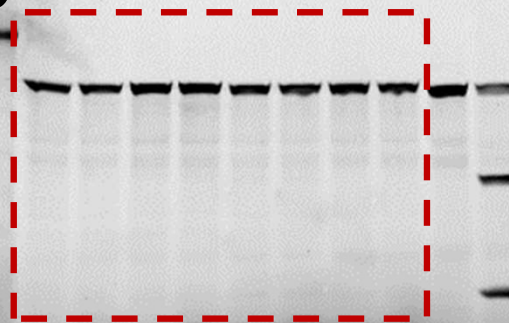

8 243-254 — 273-284

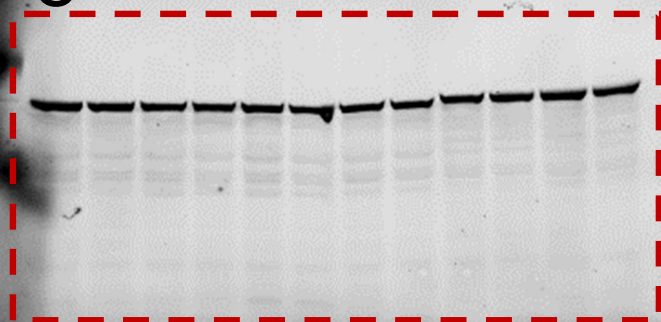

9 279-290 — 315-326

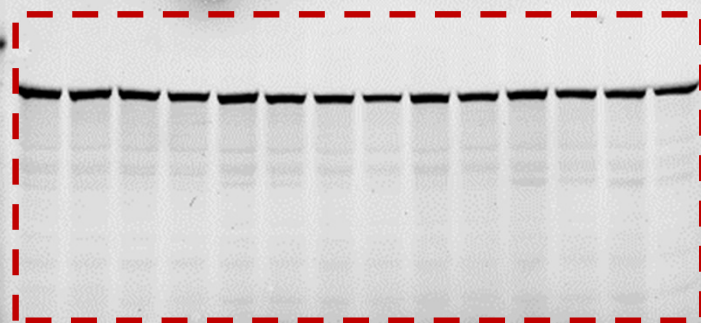

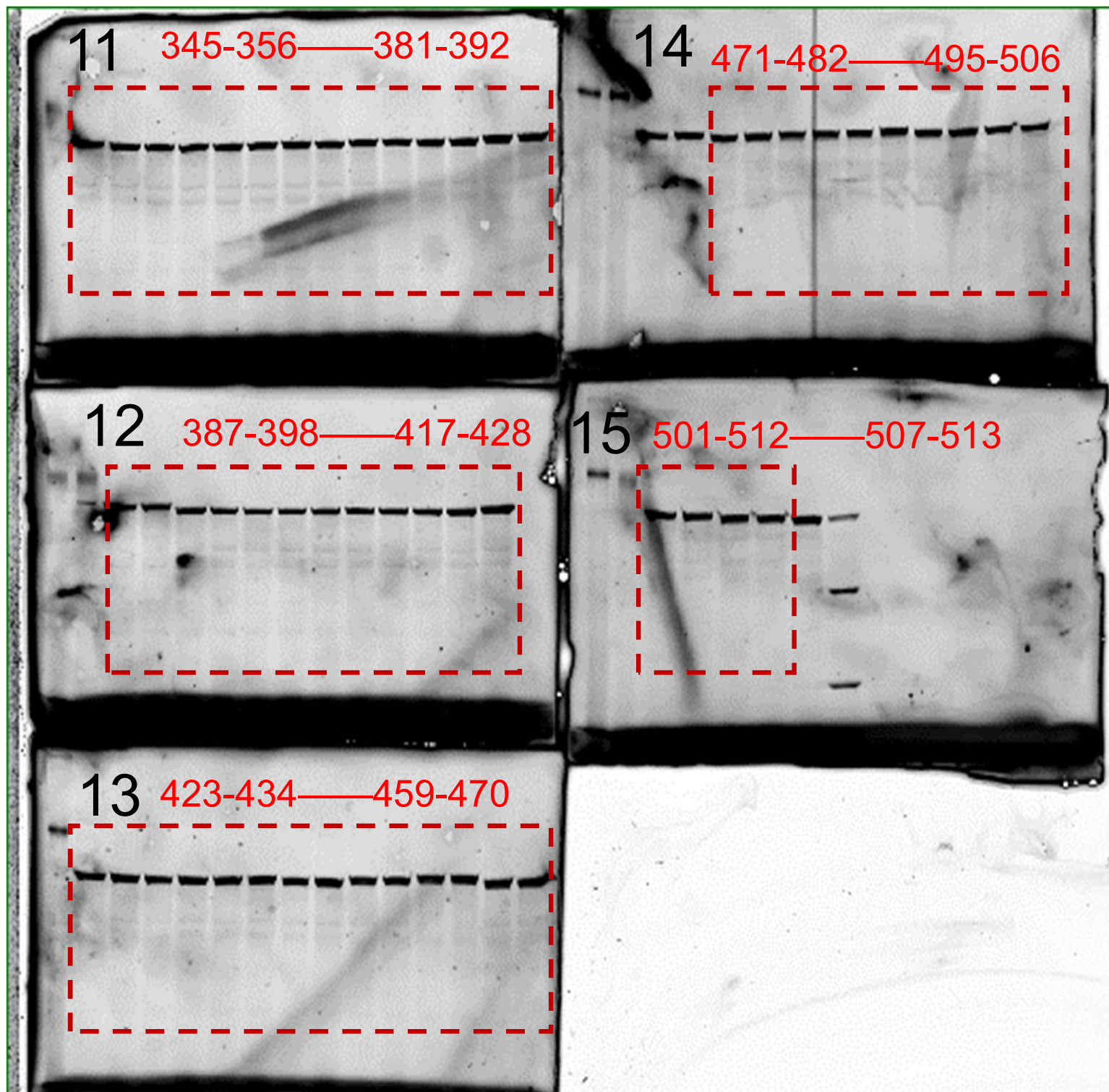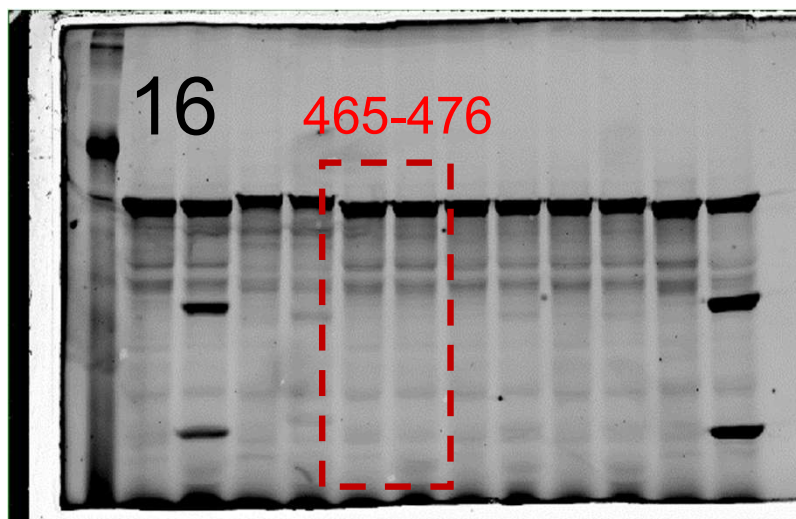

Supplement: S2 File — (ZIP) [file ppat.1012209.s003.zip › S2 file/raw data-figS4.pdf]

# All images for Figure 4A

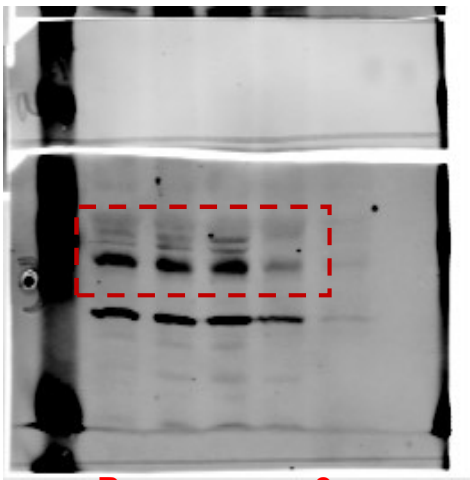

**Pro-caspase-3**

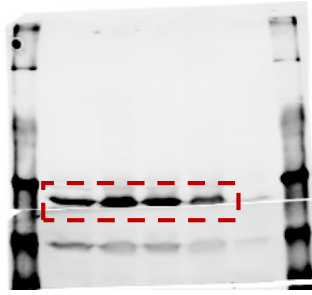

**Pro-caspase-8**

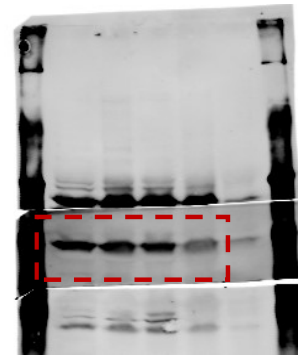

**Pro-caspase-9**

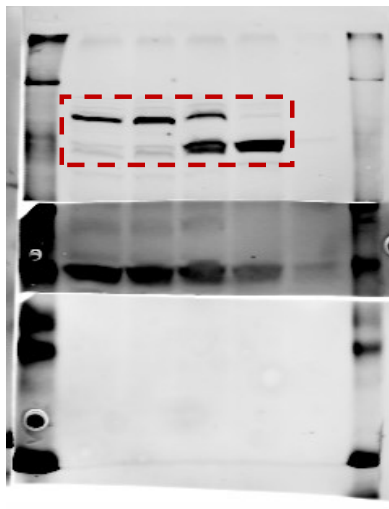

**PARP**

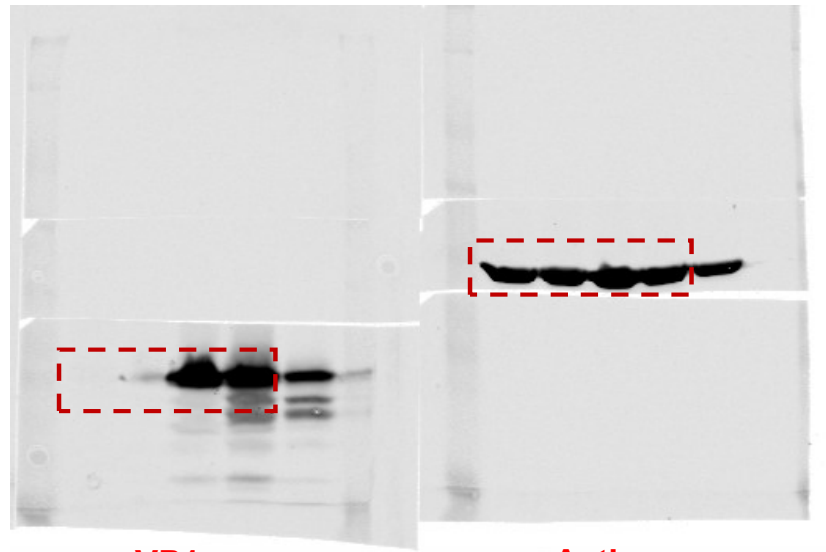

**VP1**

**Actin**

Supplement: S2 File — (ZIP) [file ppat.1012209.s003.zip › S2 file/raw data-fig4A.pdf]

# All images for Figure 9A

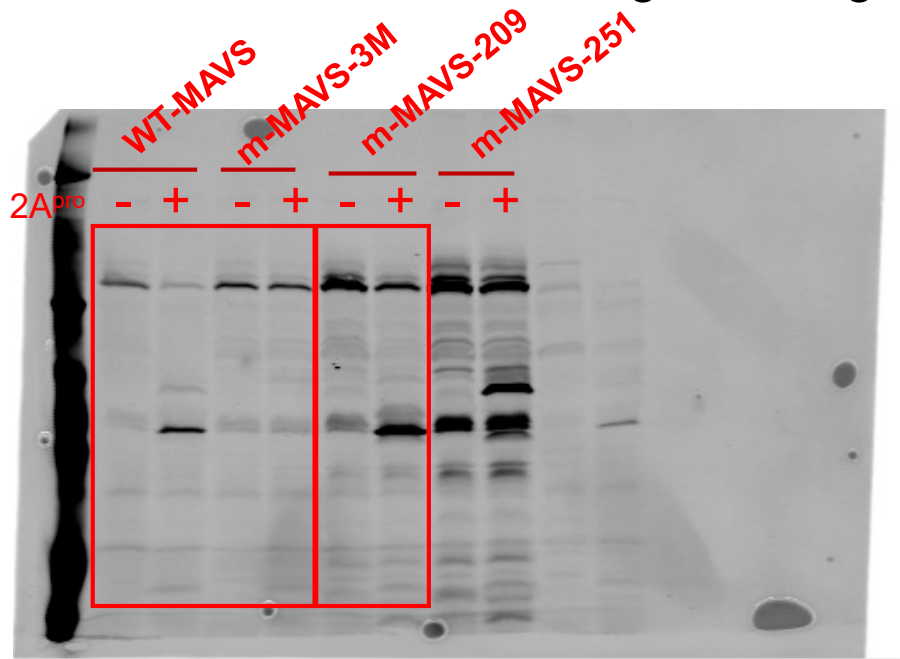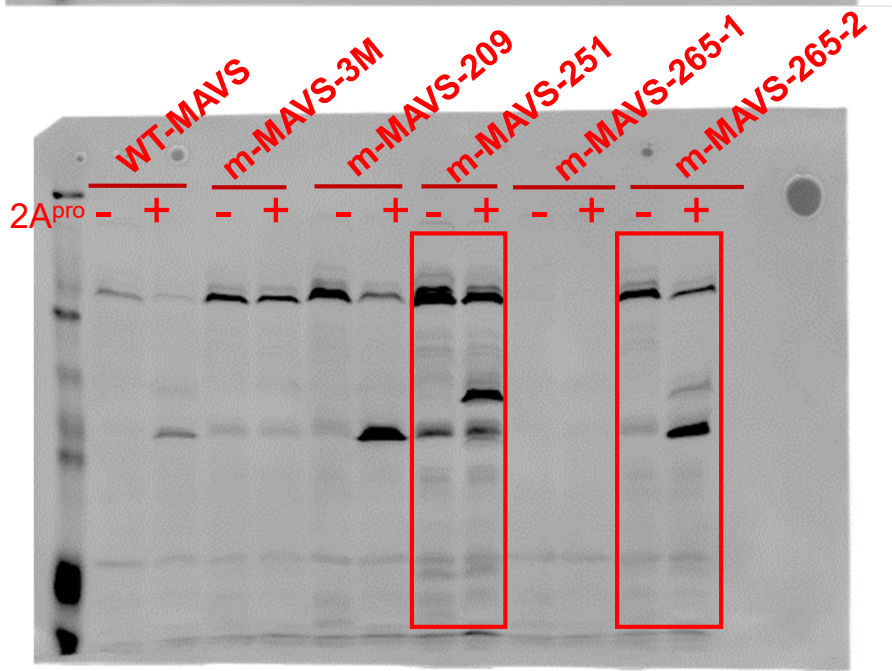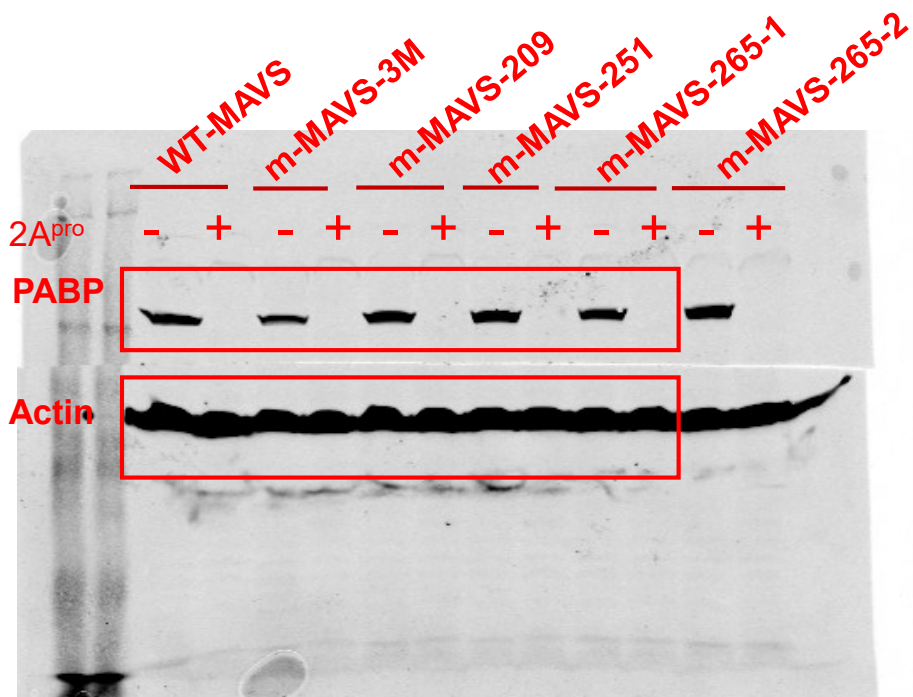

Supplement: S2 File — (ZIP) [file ppat.1012209.s003.zip › S2 file/raw data-fig9A.pdf]

Image for Figure 9E

m-MAVS-265

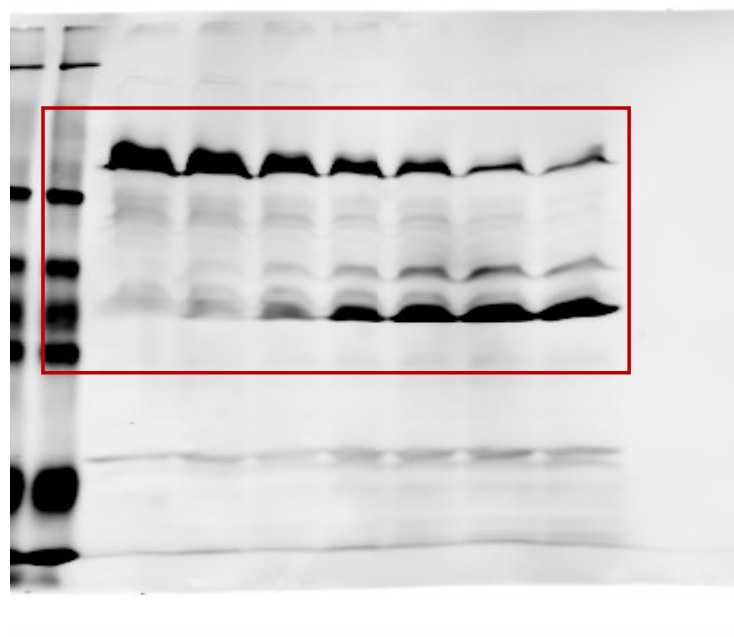

Supplement: S3 File — (ZIP) [file ppat.1012209.s004.zip › S3 file/raw data-fig9E.pdf]

Image for Figure 4C

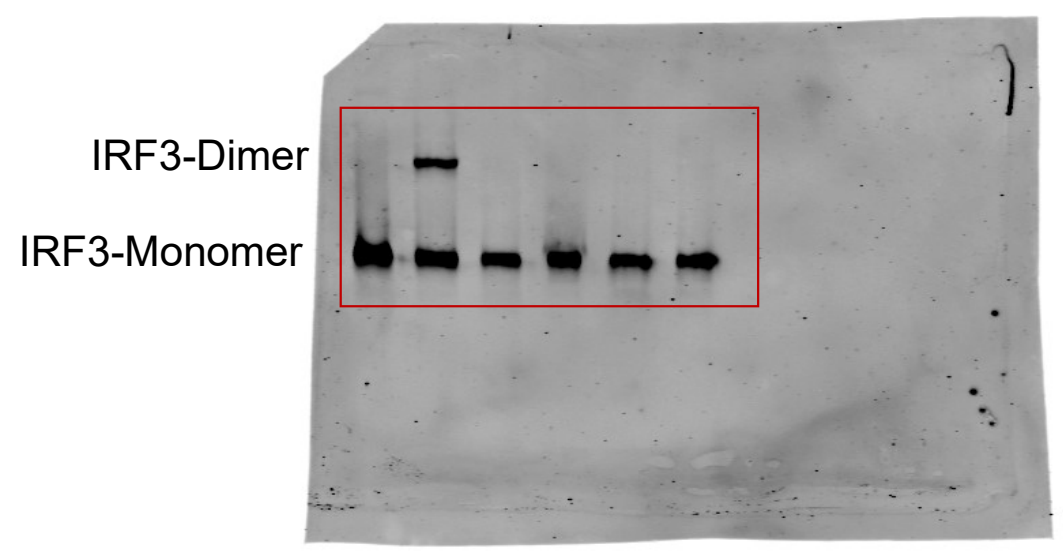

Supplement: S3 File — (ZIP) [file ppat.1012209.s004.zip › S3 file/raw data-fig4C.pdf]

All images for Figure 7D

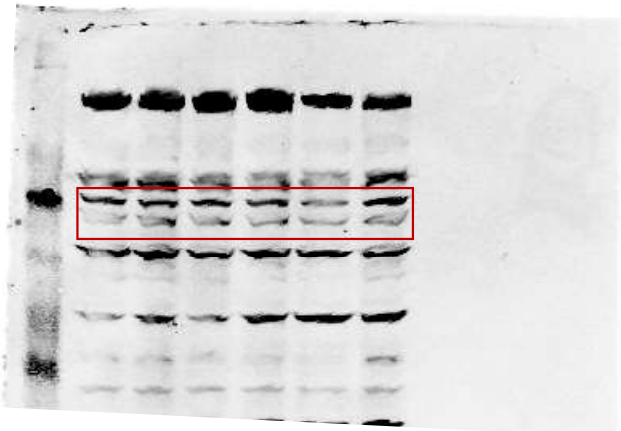

MAVS

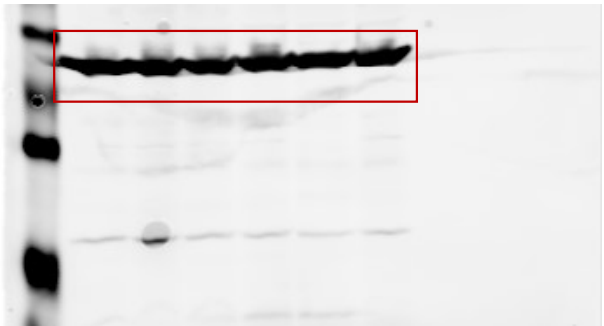

Actin

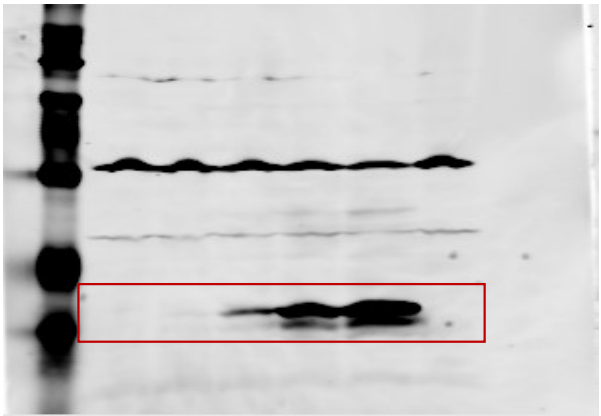

2A

Supplement: S3 File — (ZIP) [file ppat.1012209.s004.zip › S3 file/raw data-fig7D.pdf]

New 7E-2A110

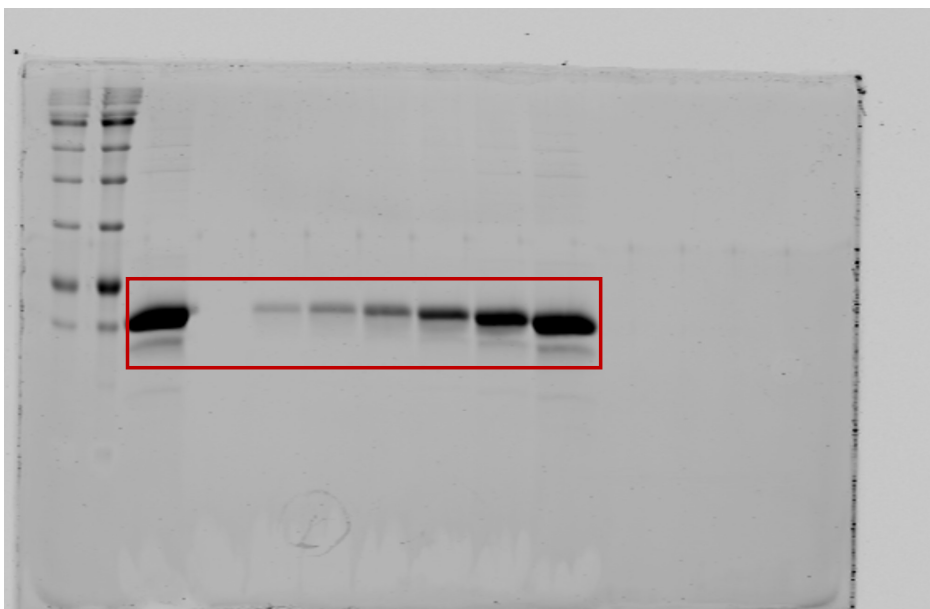

Supplement: S3 File — (ZIP) [file ppat.1012209.s004.zip › S3 file/new 7E-2A110.pdf]

# All images for Figure 4B

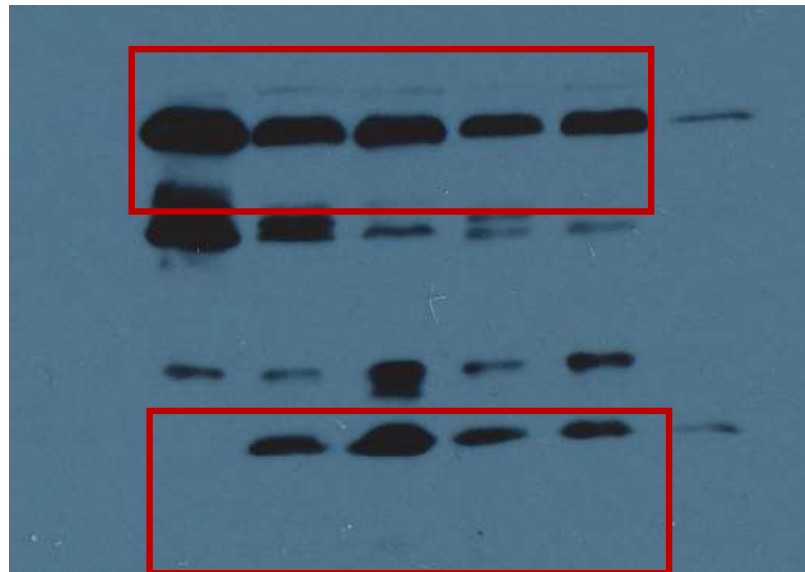

MAVS

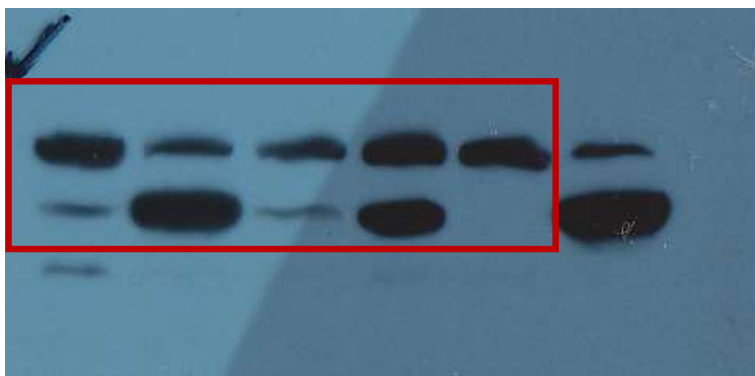

PARP

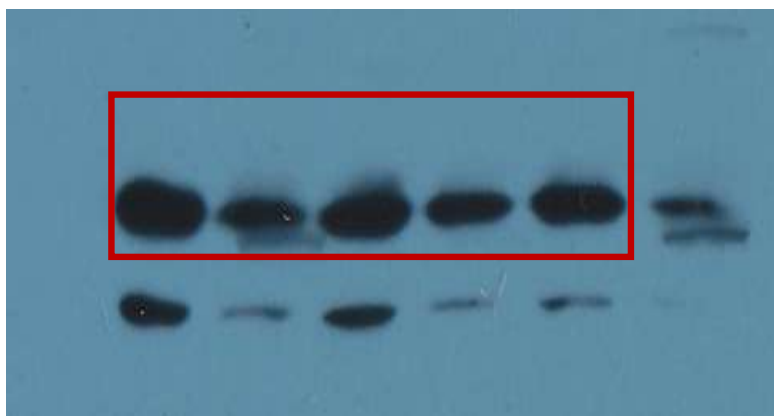

Pro-caspase-3

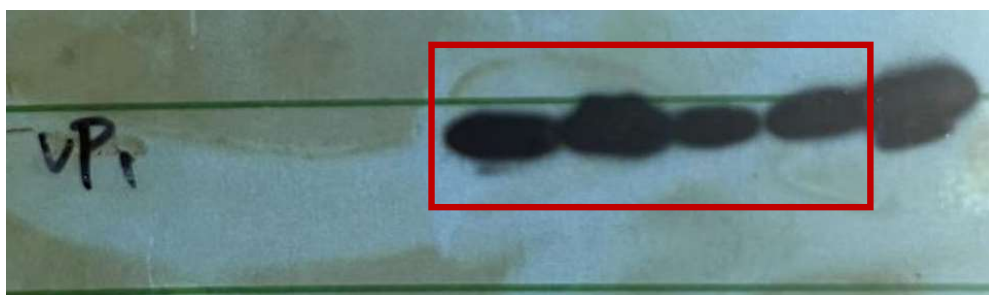

VP1

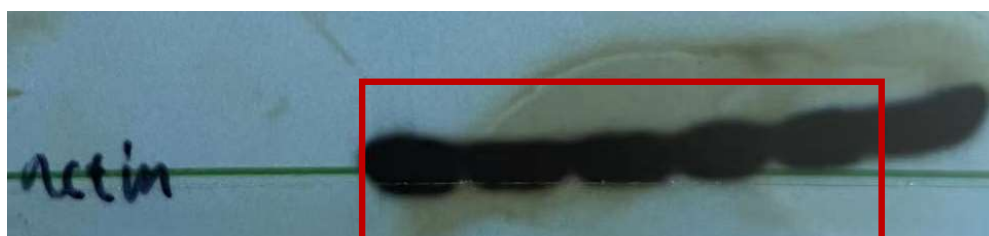

Actin

Supplement: S3 File — (ZIP) [file ppat.1012209.s004.zip › S3 file/raw data-fig4B.pdf]

Image for Figure 9D

m-MAVS-251

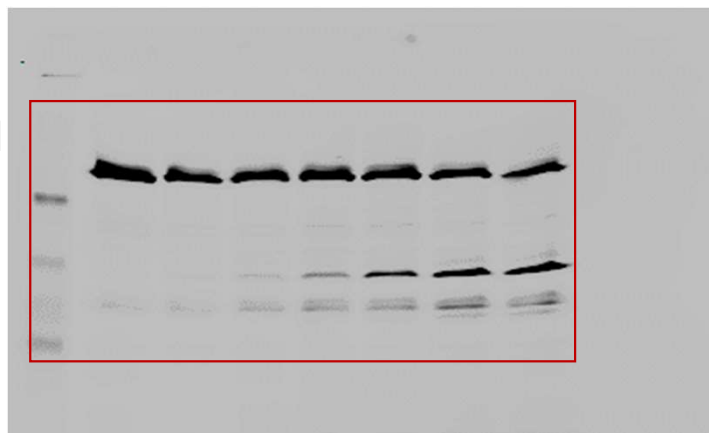

Supplement: S3 File — (ZIP) [file ppat.1012209.s004.zip › S3 file/raw data-fig9D.pdf]

Image for Figure 9C

WT-MAVS

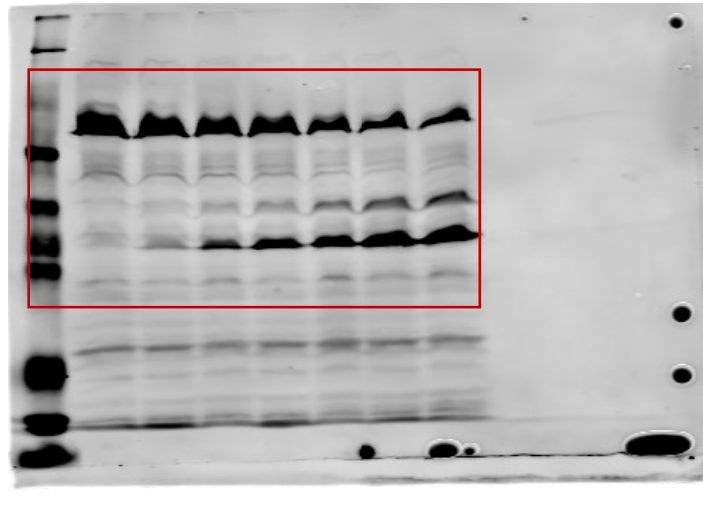

Supplement: S3 File — (ZIP) [file ppat.1012209.s004.zip › S3 file/raw data-fig9C.pdf]

All images for figure 7C

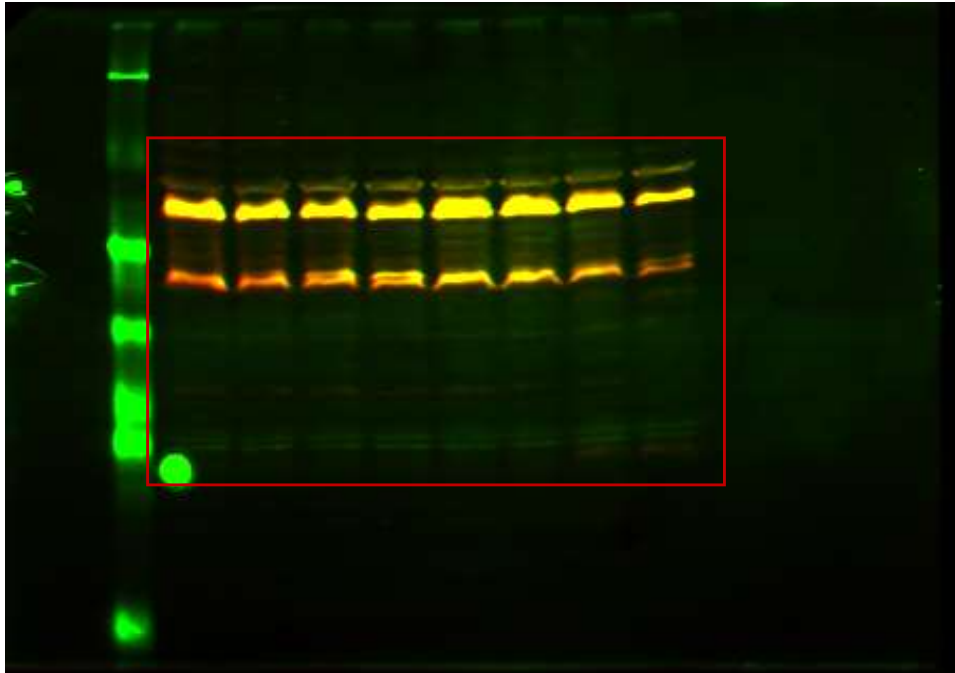

MAVS

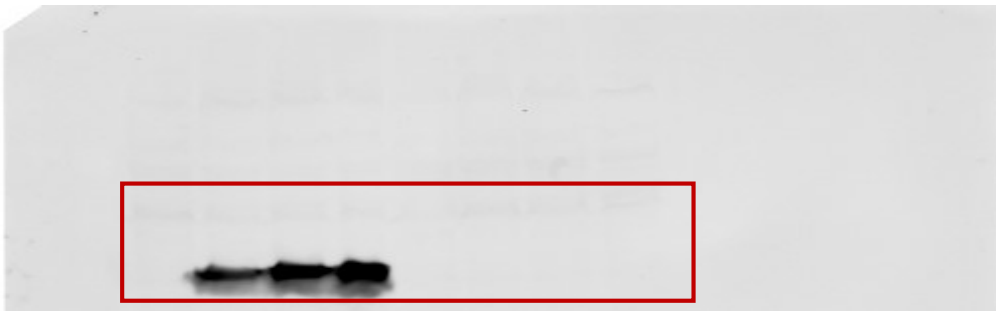

3C

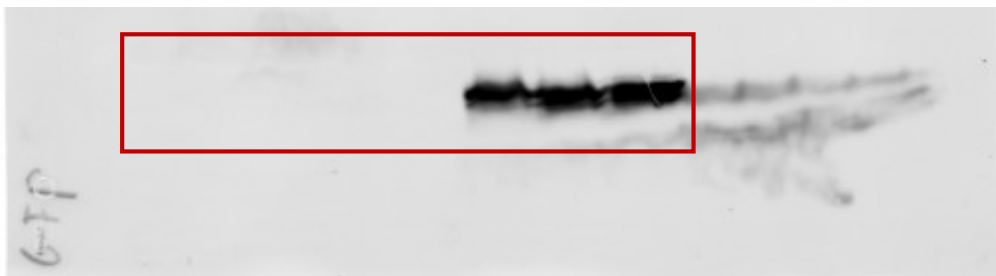

3ABC

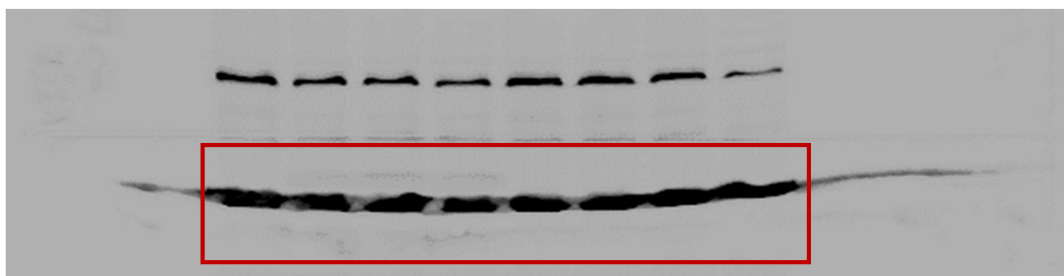

Actin

Supplement: S3 File — (ZIP) [file ppat.1012209.s004.zip › S3 file/raw data-fig7C.pdf]

Repeated Figure 2B

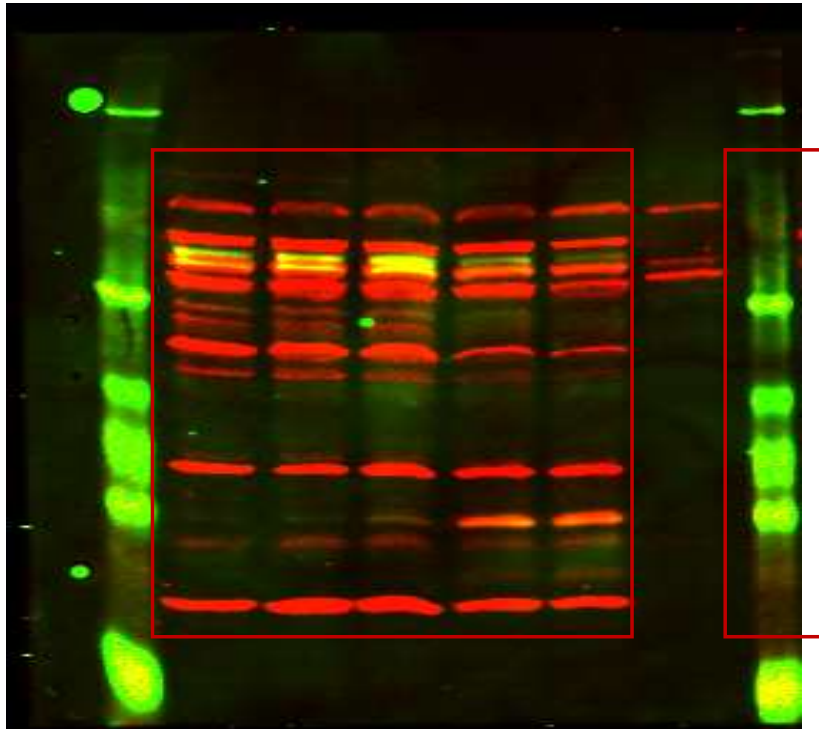

Supplement: S3 File — (ZIP) [file ppat.1012209.s004.zip › S3 file/repeated fig2B.pdf]

All images for Figure 2A

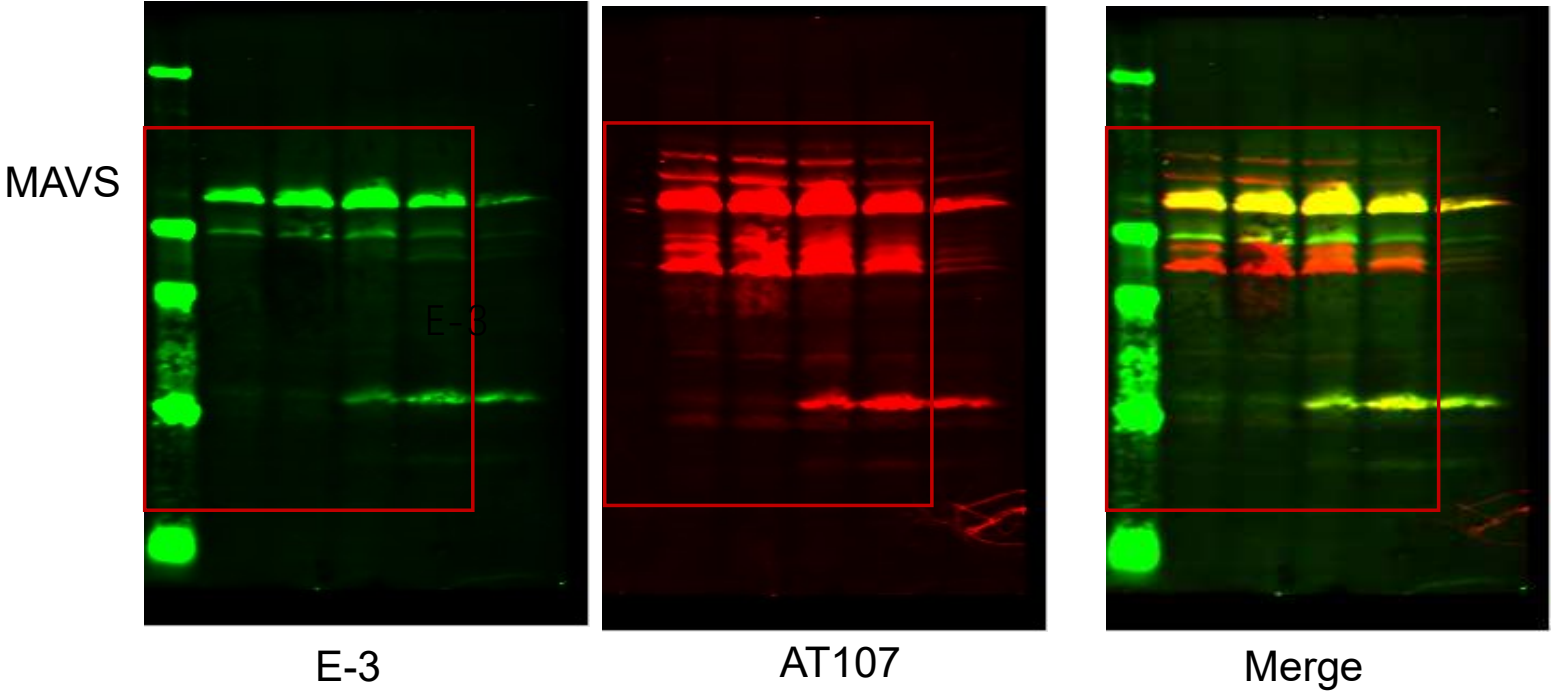

Supplement: S3 File — (ZIP) [file ppat.1012209.s004.zip › S3 file/raw data-fig2A.pdf]

# All images for Figure 7A

Anti-MAVS  
(E-3)

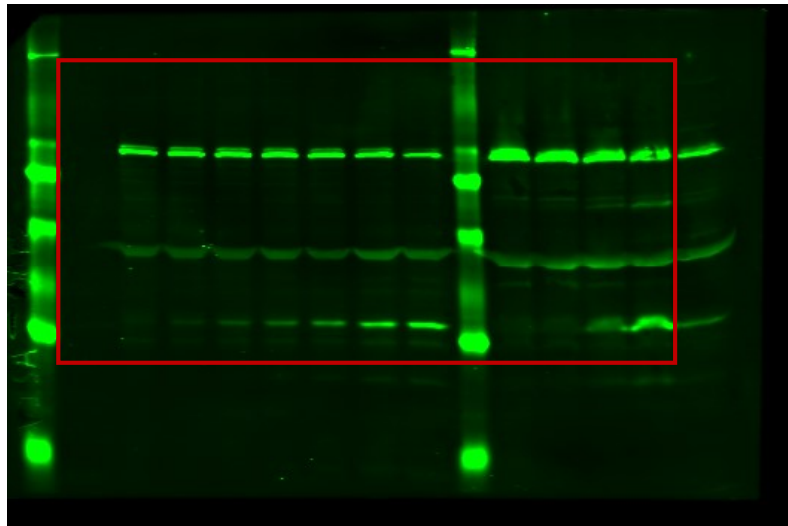

Anti-MAVS  
(AT107)

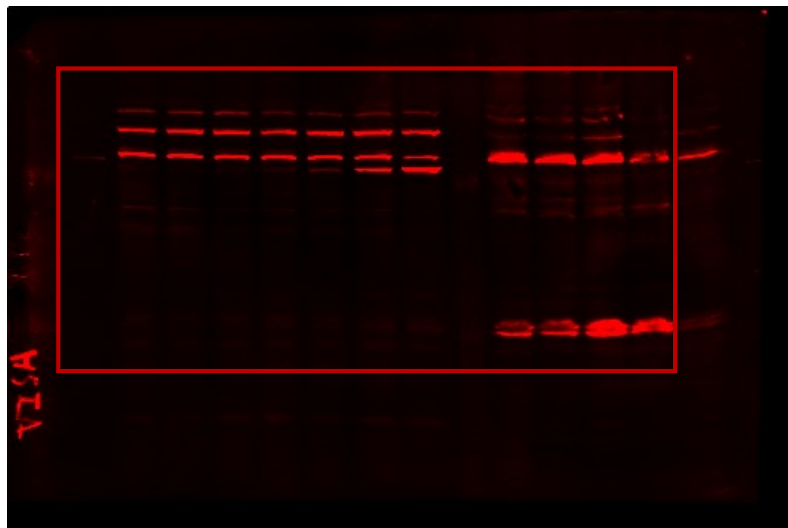

Merge

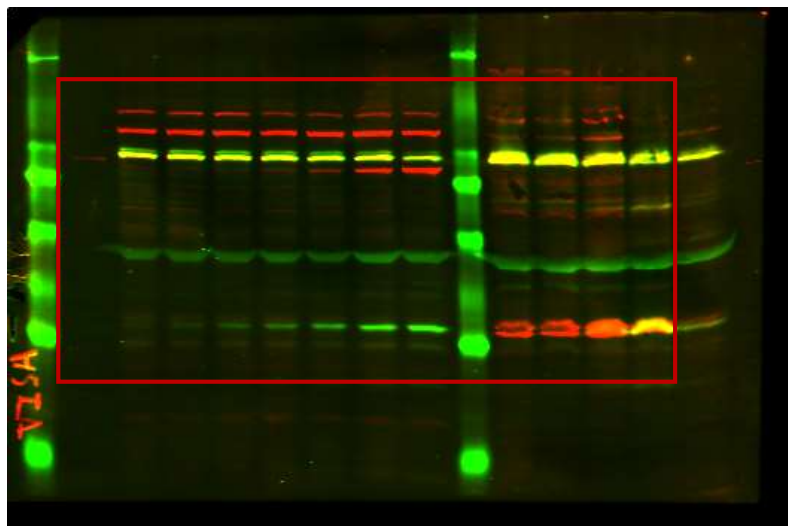

Supplement: S3 File — (ZIP) [file ppat.1012209.s004.zip › S3 file/raw data-fig7A.pdf]

# All images for Figure 7B

Anti-MAVS  
(E-3)

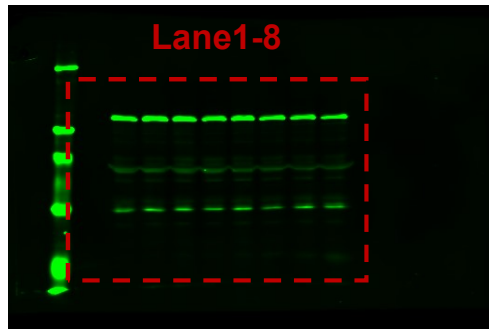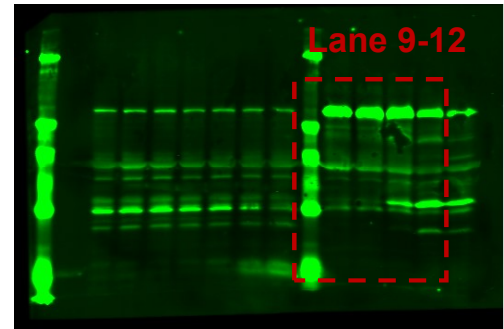

Anti-MAVS  
(AT107)

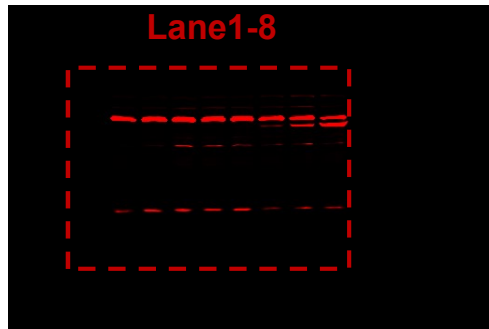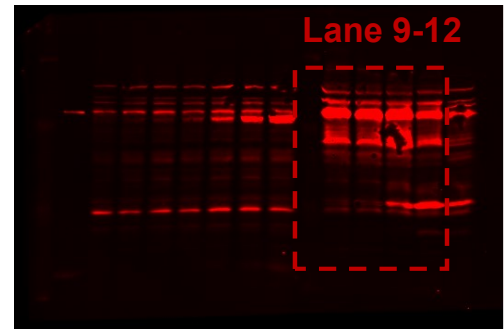

Merge

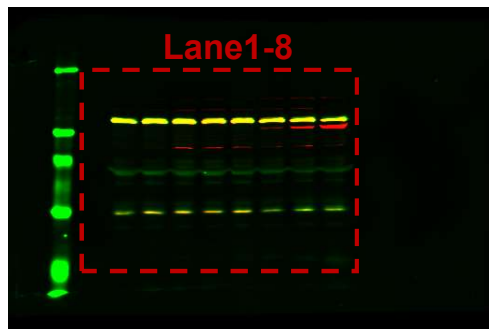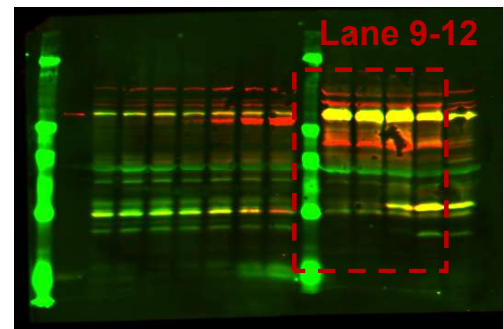

Supplement: S3 File — (ZIP) [file ppat.1012209.s004.zip › S3 file/raw data-fig7B.pdf]
