# Supplementary material for: Correction: Enterovirus 71 Protease 2Apro Targets MAVS to Inhibit Anti-Viral Type I Interferon Responses
Source: PLoS Pathog. 2024 May 6;20(5):e1012209. doi: 10.1371/journal.ppat.1012209 (PMC11073730; doi:10.1371/journal.ppat.1012209)
Supplement: S2 File — (ZIP) [file ppat.1012209.s003.zip › S2 file/raw data-fig2B.pdf]

All Images for Figure 2B

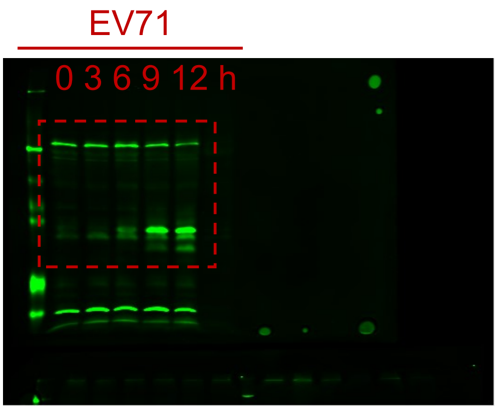

Raw Data for Figure 2B E-3 panel

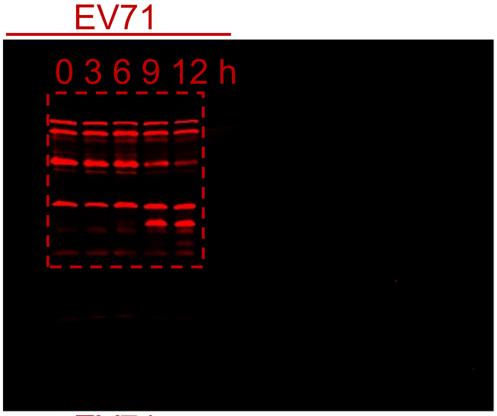

Raw Data for Figure 2B AT107 panel

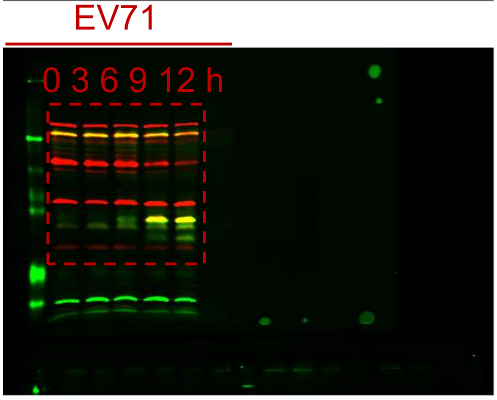

Raw Data for Figure 2B Merge panel
